# Tailoring a 3D Covalent Organic Framework Toward Facile Functionalization

**DOI:** 10.1002/smll.202511087

**Published:** 2025-12-08

**Authors:** Silas O. Frimpong, Maxima Pacheco, Haomiao Xie, Andrea N. Zeppuhar, Omar K. Farha, Mercedes K. Taylor

**Affiliations:** ^1^ Department of Chemistry and Biochemistry University of Maryland College Park MD 20742 USA; ^2^ Department of Chemistry and International Institute for Nanotechnology Northwestern University Evanston IL 60208 USA

**Keywords:** covalent organic frameworks, COF‐300, linkage functionalization, microcrystal electron diffraction (microED), post‐synthetic modification

## Abstract

Three‐dimensional covalent organic frameworks (3D COFs) are notably crystalline and stable, but their architectures and monomer structures make them difficult to functionalize. Here, a new strategy is presented to render COF‐300 and other imine‐linked frameworks amenable to facile functionalization in the last step of synthesis. By reducing the imine linkages to secondary amines and appending them with acetyl halide groups, the linkages are converted to electrophiles that can be readily reacted with nucleophilic guests. This route yields eight new COF‐300 derivatives, bearing chloroacetyl, bromoacetyl, azide, cyano, amino, hydroxyl, methoxy, or thiomethyl groups appended to the inter‐monomer linkages. The new materials are characterized through solid‐state NMR, infrared spectroscopy, and powder X‐ray diffraction, among other techniques, finding that the reported linkage transformations proceed to complete conversion while retaining the crystallinity of the materials. Microcrystal electron diffraction (microED) data are used to solve the evacuated structure of the amine‐linked framework COF‐300‐AR for the first time, providing conclusive evidence of this framework's guest‐induced phase change, along with the structure of the new framework COF‐300‐NH_2_. Finally, COF‐300‐NH_2_ is shown to have significantly improved adsorption capacity for CO_2_ and perfluoroalkyl substances (PFAS), highlighting the benefits of this synthetic strategy for the generation of customized adsorbents.

## Graphical abstract

Nucleophilic groups are installed onto the linkages of the well‐known covalent organic framework, COF‐300, which allows for the synthesis of eight new COF‐300 derivatives. The structure of the amine‐appended framework COF‐300‐NH_2_ is solved using microED, and this material exhibits significantly improved adsorption capacity for CO_2_ and perfluoroalkyl substances.

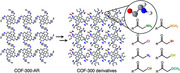

## 1 Introduction

The organic materials known as covalent organic frameworks (COFs) are crystalline^[^ ^1^, ^2^, ^3^, ^4^, ^5^, ^6^ ^]^ and porous,^[^ ^7^, ^8^, ^9^, ^10^ ^]^ making them attractive platforms for custom functionalization.^[^ ^11^, ^12^, ^13^, ^14^, ^15^ ^]^ The framework termed COF‐300^[^ ^2^ ^]^ is a blue‐ribbon example of these materials: The 3D connectivity of the monomers leads to outstanding crystallinity^[^ ^16^ ^]^ and a stable architecture, particularly when the inter‐monomer imine linkages are reduced to secondary amines.^[^ ^17^ ^]^ However, in spite of the publication of dozens of high‐impact papers on COF‐300, this framework has proved poorly amenable to functionalization.^[^ ^18^ ^]^ While COF‐300 performs well in certain separations^[^ ^19^, ^20^ ^]^ and gas storage applications,^[^ ^21^ ^]^ the inability to install desired functional groups onto the pore walls prevents chemists from developing this material toward industrial uses.

**Figure 1:**
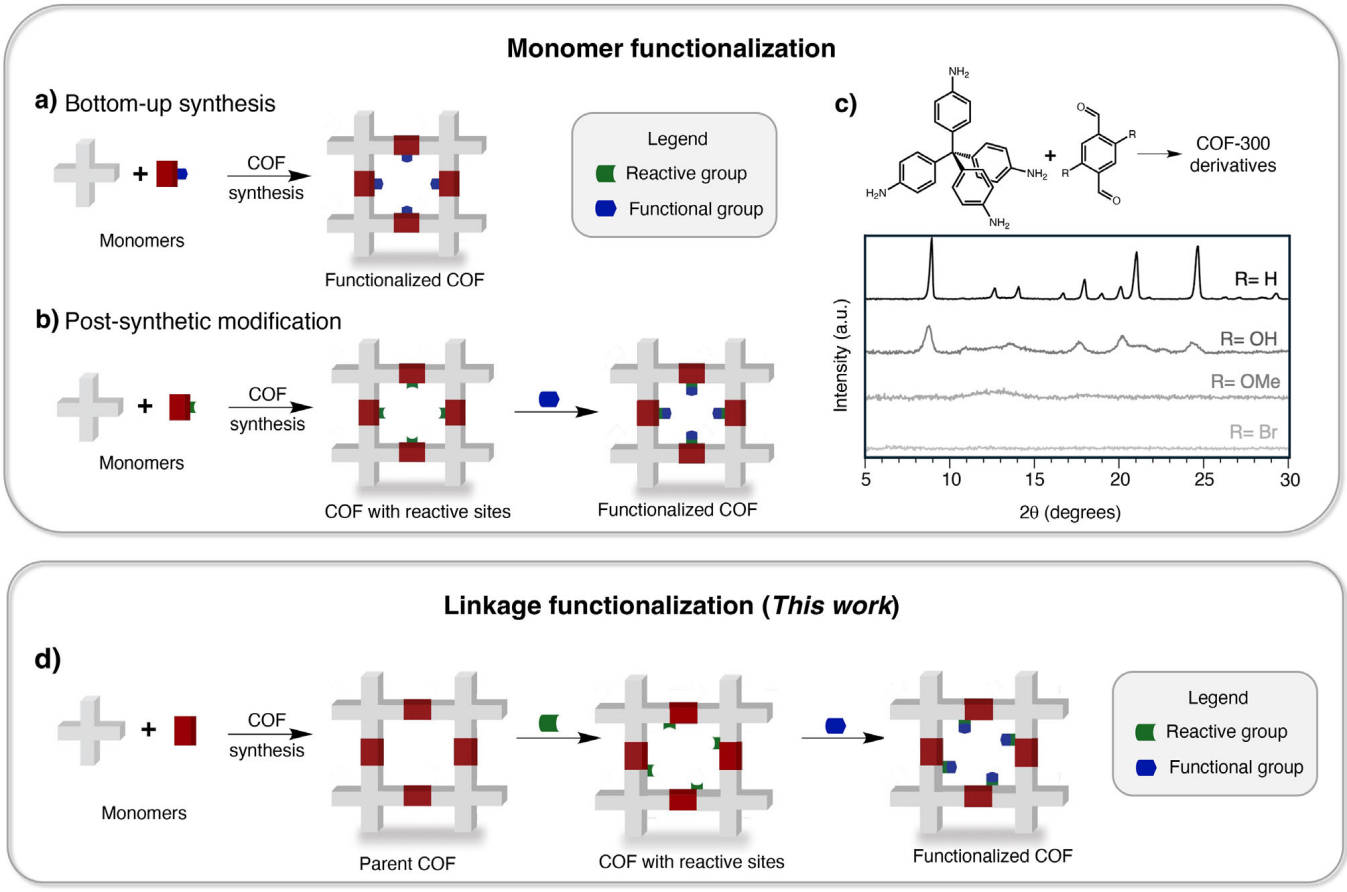
COF‐300 is poorly amenable to monomer functionalization, either through a) bottom‐up synthesis or b) post‐synthetic modification of monomers, as illustrated by c) powder X‐ray diffraction (PXRD) data for a series of synthesis attempts. d) The linkage functionalization approach reported here sidesteps these problems.

Functionality is typically installed into COF pores by introducing functional groups onto the aryl rings of one of the starting monomers (**Figure** **1**a,b). In the so‐called “bottom‐up” approach, the desired functional group is appended to the monomer prior to COF synthesis; this strategy requires that the functional group not interfere with framework formation and often adds several steps to monomer synthesis.^[^ ^22^, ^23^, ^24^, ^25^, ^26^ ^]^ A more convenient approach termed post‐synthetic functionalization installs the desired group after the COF has already formed, although this route still requires a reactive handle to be present on one of the monomers.^[^ ^27^, ^28^, ^29^, ^30^, ^31^, ^32^, ^33^ ^]^

Unfortunately, COF‐300 tolerates neither of these strategies. As shown in Figure 1c, we found that the presence of even small, simple groups on the terephthaldehyde monomer severely limits the crystallinity of COF‐300, as others have also shown.^[^ ^34^, ^35^ ^]^ The addition of such groups to the tetrakis(4‐aminophenyl)methane monomer, which already requires a 4‐step synthesis in its unfunctionalized form,^[^ ^36^, ^37^ ^]^ would be synthetically impractical and is likely to be equally disruptive to framework crystallinity. Instead, we recently showed that post‐synthetic functionalization of the linkages between monomers is a viable approach to generate a library of COF‐300 derivatives.^[^ ^38^ ^]^ By first reducing the imine linkages to yield an amine‐linked framework termed COF‐300‐AR and then converting the amine linkages to quaternary ammonium groups, we were able to transform an unimpressive adsorbent for perfluoroalkyl substances (PFAS) into an outstanding one. More recently, Wang and coworkers extended this strategy to include the addition of an aldehyde (–CHO) group to the secondary amine linkages of COF‐300‐AR.^[^ ^39^ ^]^ But the aniline‐like nature of the amine linkages in COF‐300‐AR makes them comparatively weak nucleophiles, limiting the range of functional groups that can be added and leading to long reaction times, as we showed in our prior work.^[^ ^38^ ^]^

**Scheme 1:**
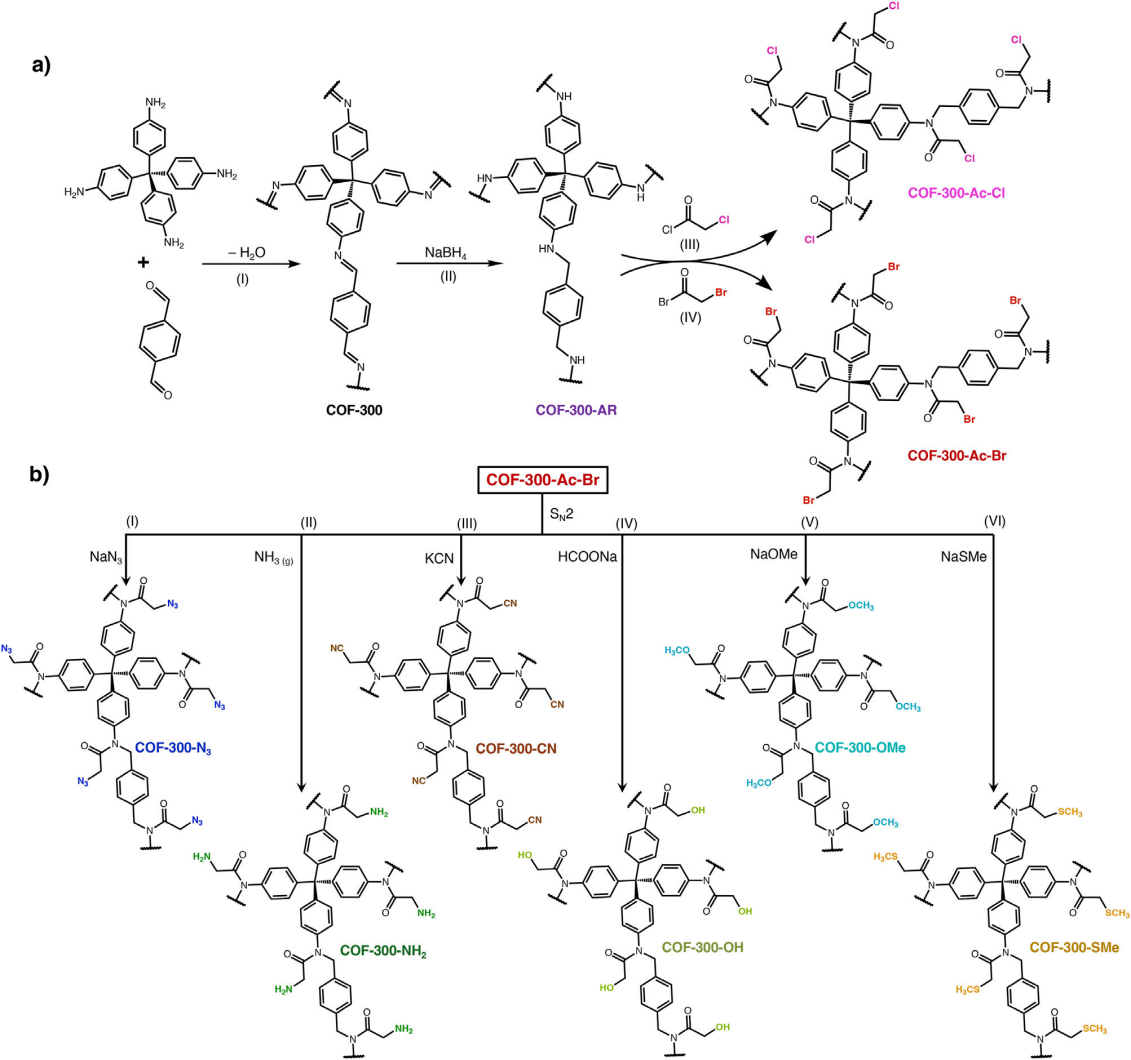
a) Route to COF‐300 derivatives with electrophilic groups appended to the linkages; (I) 1,4‐dioxane, 6 m aqueous acetic acid, 120 °C, 72 h. (II) 1,4‐benzenedicarboxylic acid (BDC), anhydrous methanol(MeOH), room temperature (rt), 24 h. (III) triethylamine(Et_3_N), anhydrous dichloromethane (DCM), rt, 72 h. (IV) Et_3_N, anhydrous DCM, 40 °C, 120 h. b) S_N_2 reactions of COF‐300‐Ac‐Br with diverse nucleophiles yield a library of functionalized COF‐300 derivatives; (I) anhydrous *N*,*N*‐dimethylformamide (DMF), 65 °C, 24 h. (II) anhydrous MeOH, 0 °C, 6 h. (III) anhydrous DMF, 65 °C for 72 h. (IV) 2:1 ethanol: water mixture, 70 °C, 24 h (V) MeOH, 65 °C, 72 h (VI) anhydrous DMF, 75 °C, 72.

Here, we have overcome this nucleophilicity problem by installing electrophiles on the linkages of COF‐300, thus reversing the S_N_2 chemistry at work. We first reduced the imine linkages of COF‐300 through reaction with NaBH_4_, yielding COF‐300‐AR, and then reacted the amine linkages of COF‐300‐AR with chloroacetyl chloride or bromoacetyl bromide (**Scheme** **1**a). The products, COF‐300‐Ac‐Cl and COF‐300‐Ac‐Br, contain alkyl halide groups dangling from the linkages, poised to react with nucleophilic guests that enter the pores. We then found that in a single step, COF‐300‐Ac‐Br could react with carbon‐, nitrogen‐, oxygen‐, and sulfur‐based nucleophiles, generating a family of derivatives with diverse functionality (Scheme 1b). A range of spectroscopic evidence shows that these reactions go to completion, converting the entirety of the framework linkages to the desired group. Powder X‐ray diffraction (PXRD) studies show that this suite of linkage transformations leaves the framework structure intact, an encouraging sign for the use of this strategy to create designer adsorbents with precisely controlled pore architectures. Additionally, microcrystal electron diffraction (microED) data yields crystal structures for the NH_2_‐functionalized sample, COF‐300‐NH_2_, along with the COF‐300‐AR intermediate. Excitingly, the COF‐300‐AR structure adopts a collapsed conformation under vacuum, providing conclusive evidence of the structural phase change this material undergoes upon evacuation and supporting previous speculation about the flexibility of this framework. Finally, PFAS and CO_2_ adsorption measurements show COF‐300‐NH_2_ to perform significantly better than the COF‐300‐AR precursor, supporting the industrial applicability of this linkage functionalization approach.

## 2 Results and Discussion

**Figure 2:**
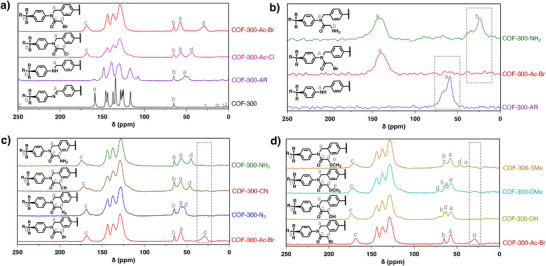
a) ^13^C cross‐polarization magic angle spinning solid‐state NMR (ssNMR) spectra for COF‐300 (black), COF‐300‐AR (purple), COF‐300‐Ac‐Cl (pink), and COF‐300‐Ac‐Br (red). b) ^15^N ssNMR spectra for COF‐300‐AR (purple), COF‐300‐Ac‐Br (red), and COF‐300‐NH_2_ (green) c) ^13^C ssNMR spectra for COF‐300‐Ac‐Br (red), COF‐300‐N_3_ (blue), COF‐300‐CN (brown), and COF‐300‐NH_2_ (green). d) 13C ssNMR for COF‐300‐Ac‐Br (red), COF‐300‐OH (yellow), COF‐300‐OMe (turquoise), and COF‐300‐SMe (orange).

The synthesis of COF‐300 was carried out by reacting tetrakis(4‐aminophenyl)methane (TAPM) with terephthaldehyde (TPA) in a solution of 1,4‐dioxane and 6 m aqueous acetic acid.^[^ ^2^ ^]^ The mixture was degassed, sealed, and heated at 120 °C for 72 h. The formation of crystalline COF‐300 was confirmed using powder X‐ray diffraction (PXRD) (Figure S13, Supporting Information) and solid‐state ^13^C cross‐polarization magic‐angle spinning NMR (^13^C ssNMR) spectroscopy (**Figure** **2**a, black). The ^13^C ssNMR shows an imine peak at 159 ppm and a quaternary carbon from TAPM in the framework at 65 ppm. Aromatic carbon peaks are observed between 100 and 150 ppm.

Treating the imine‐linked framework COF‐300 with NaBH_4_ and 1,4‐benzenedicarboxylic acid (BDC) in anhydrous methanol at room temperature for 24 h yielded the amine‐linked framework COF‐300‐AR.^[^ ^17^ ^]^ The successful conversion of COF‐300 to COF‐300‐AR was confirmed using ^13^C ssNMR spectroscopy, which shows the disappearance of an imine carbon at 159 ppm and the appearance of an amine carbon at 52 ppm (Figure 2a; Figure S11, Supporting Information, purple).

**Figure 3:**
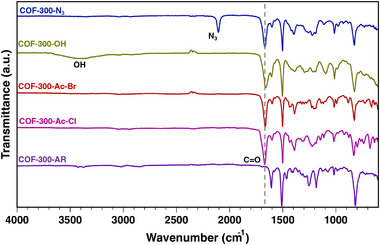
Fourier‐transform infrared (FTIR) spectra for the COF‐300 derivatives with diagnostic FTIR signals: COF‐300‐AR (purple), COF‐300‐Ac‐Cl (pink), COF‐300‐Ac‐Br (red), COF‐300‐OH (yellow), and COF‐300‐N_3_ (blue).

To convert the amine linkages of COF‐300‐AR into electrophilic groups, we first targeted the installation of an alkyl chloride through reaction with chloroacetyl chloride. Combining COF‐300‐AR with chloroacetyl chloride and triethylamine in anhydrous dichloromethane (DCM) at room temperature for 72 h yielded the chloroacetylated product, COF‐300‐Ac‐Cl. The successful synthesis of COF‐300‐Ac‐Cl was confirmed with ^13^C ssNMR and Fourier‐transform infrared spectroscopy (FTIR). The appearance of new peaks was observed at 43 and 168 ppm in the ssNMR spectrum, corresponding to the carbon of the methylene chloride (CH_2_‐Cl) and the carbonyl carbon from the acetyl group, respectively (Figure 2a, pink). Additionally, an intense carbonyl stretch at 1700 cm^−1^ in the FTIR spectrum further confirms successful acetylation (**Figure** **3**, pink).

Preliminary efforts to react COF‐300‐Ac‐Cl with nucleophilic guests showed incomplete conversion, so we hypothesized that replacing the chloride with a better leaving group would improve reactivity. To this end, we reacted COF‐300‐AR with bromoacetyl bromide and triethylamine in anhydrous DCM at 40 °C for 120 h to generate COF‐300‐Ac‐Br. Upon completion, ^13^C ssNMR showed the appearance of new peaks at 29 and 168 ppm, corresponding to the carbon of the methylene bromide (CH_2_‐Br) and the carbonyl carbon (C═O) from the acetyl group, respectively (Figure 2a, red). Further evidence of bromoacetylation was provided by FTIR, which shows the appearance of a strong carbonyl stretch from the acetyl group at 1700 cm^−1^ for COF‐300‐Ac‐Br (Figure 3, red). To determine the extent of conversion of COF‐300‐AR to COF‐300‐Ac‐Br, we collected solid‐state ^15^N cross‐polarization magic‐angle spinning NMR (^15^N ssNMR) spectra. The disappearance of the secondary amine nitrogen peak at 60 ppm and the emergence of an amide nitrogen peak at 140 ppm in ^15^N ssNMR indicate the complete conversion of COF‐300‐AR to COF‐300‐Ac‐Br (Figure 2b, purple and red, respectively). Energy dispersive X‐ray spectroscopy (EDX) of COF‐300‐Ac‐Br also shows a uniform distribution of bromine in the framework (Figure S17, Supporting Information).

With COF‐300‐Ac‐Br in hand, we synthesized a library of functionalized COFs, as detailed below. Chemical characterization of the functionalized derivatives was accomplished with ssNMR, along with FTIR where appropriate, while structural characterization was achieved through PXRD, scanning electron microscopy (SEM), gas adsorption, and microED. We note that the functionalized COF‐300 derivatives discussed below were not characterized with EDX due to the error associated with lighter atoms, such as nitrogen and oxygen, in this technique.

COF‐300‐N_3_ was synthesized by reacting COF‐300‐Ac‐Br with sodium azide (NaN_3_) in anhydrous *N*,*N*‐dimethylformamide (DMF) at 65 °C for 24 h. The carbon of the alkyl bromide group on COF‐300‐Ac‐Br provides a diagnostic ssNMR peak for this transformation (Figure 2c, red). The peak corresponding to this carbon appears at 29 ppm for COF‐300‐Ac‐Br but shifts to 54 ppm for COF‐300‐N_3_, with no residual peak intensity at 29 ppm, indicating complete conversion of the alkyl bromide group to an alkyl azide (Figure 2c, blue). Additionally, an intense azide stretch at 2180 cm^−1^ in the FTIR spectrum further confirms the successful synthesis of COF‐300‐N_3_ (Figure 3, blue).

The synthesis of a cyano‐appended framework, COF‐300‐CN, was carried out by reacting COF‐300‐Ac‐Br with potassium cyanide in anhydrous DMF at 65 °C for 72 h. Again, the shifting of the alkyl bromide peak from 29 to 46 ppm in the ^13^C ssNMR spectrum of the product indicates complete conversion of the bromide to a cyano group (Figure 2c, brown). Next, a framework bearing primary amines, COF‐300‐NH_2_, was synthesized by bubbling excess ammonia gas (NH_3(g)_) through a methanolic suspension of COF‐300‐Ac‐Br 0 °C for 6 h. We note that the NH_3(g)_ was conveniently generated in situ, as shown in Figure S44 (Supporting Information). The ^13^C ssNMR spectrum of the product shows the alkyl bromide carbon (C‐Br) peak at 29 ppm to shift to 45 ppm, again suggesting full conversion (Figure 2c, green). Additionally, ^15^N ssNMR spectroscopy of the product shows the appearance of a primary amine peak at 24 ppm (Figure 2b, green), unambiguously confirming the installation of –NH_2_ groups throughout COF‐300‐NH_2_.

A hydroxyl‐containing framework, COF‐300‐OH, was synthesized by reacting COF‐300‐Ac‐Br with sodium formate in a 2:1 ethanol: water mixture at 70 °C for 24 h, using a catalytic amount of sodium hydroxide. The shifting of the alkyl bromide carbon (C‐Br) from 29 to 62 ppm in the ^13^C ssNMR spectrum indicates complete conversion (Figure 2d, yellow). A broad band indicating an O─H stretch is observed ≈3500 cm^−1^ in the FTIR spectrum, further confirming the successful synthesis of COF‐300‐OH (Figure 3, yellow). Another oxygen‐containing framework, COF‐300‐OMe, was synthesized by reacting COF‐300‐Ac‐Br with sodium methoxide in methanol at 65 °C for 72 h. The ^13^C ssNMR spectrum of the product shows the alkyl bromide carbon (C‐Br) peak to shift from 29 to 71 ppm, along with the appearance of a peak at 62 ppm corresponding to the methoxy carbon (OCH_3_), which together support the complete conversion of COF‐300‐Ac‐Br to COF‐300‐OMe (Figure 2d, turquoise).

The last COF in the library, COF‐300‐SMe, was synthesized by reacting COF‐300‐Ac‐Br with sodium methanethiolate in anhydrous DMF at 75 °C for 72 h. The product was characterized with ^13^C ssNMR, which showed the shifting of the alkyl bromide carbon (C‐Br) peak from 29 to 46 ppm and the appearance of a new peak at 37 ppm, corresponding to the terminal methyl group appended to the sulfur atom (Figure 2d, orange). Thus, the ^13^C ssNMR results indicate complete conversion of the alkyl bromides to thioethers.

**Figure 4:**
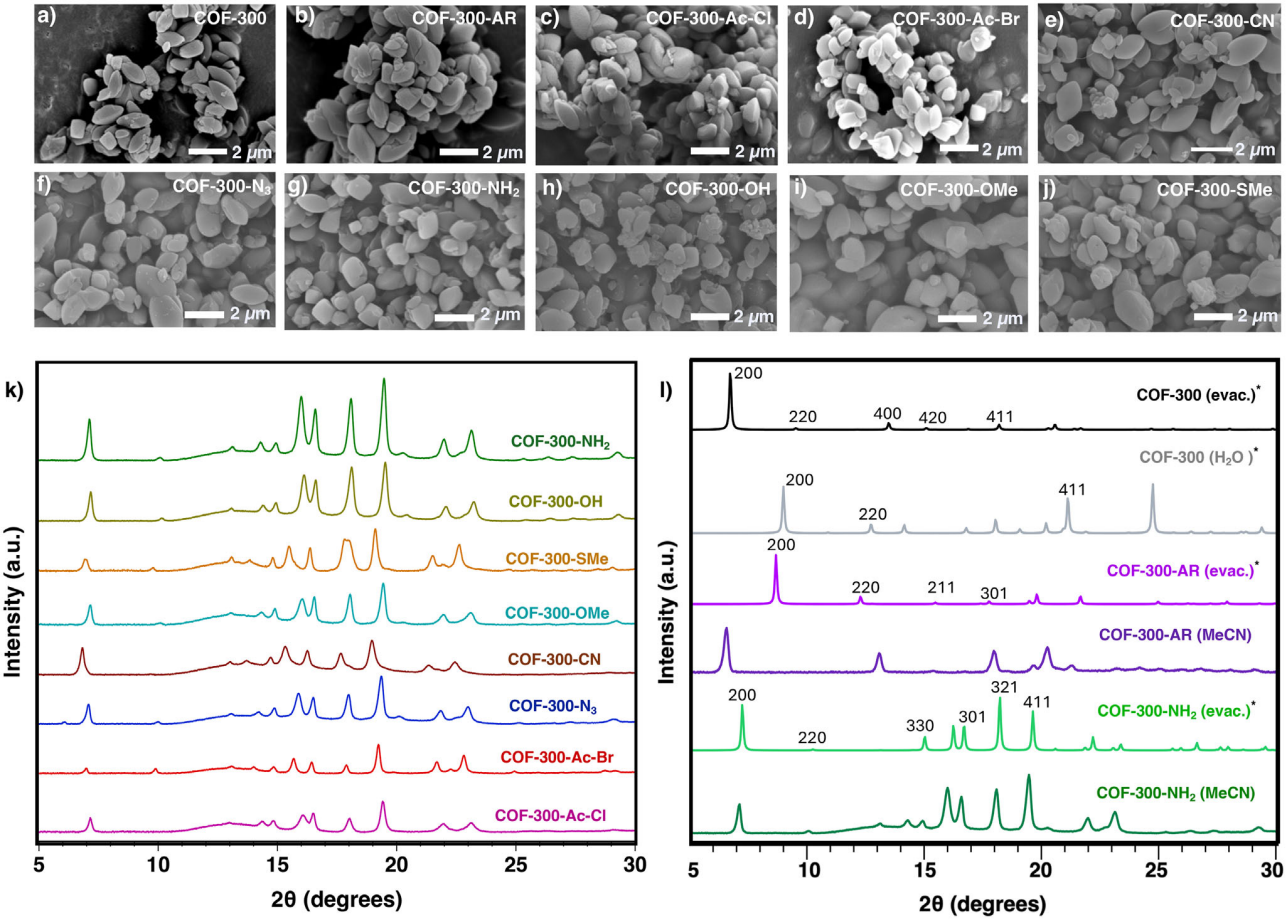
a–j). Scanning electron microscopy (SEM) images for the series of reported materials. k) Powder X‐ray diffraction (PXRD) patterns for the functionalized COF‐300 derivatives. l) PXRD patterns comparing evacuated and solvated phases for COF‐300, COF‐300‐AR, and COF‐300‐NH_2_. The PXRD patterns labeled with an asterisk (^*^) were simulated from the respective.cif files and are labelled with each peak's respective Miller indices, while the other PXRD patterns were collected on bulk powder samples. The.cif files for COF‐300 (evac.) and COF‐300 (H_2_O) are given in ref.,^[^ ^16^ ^]^ and those for COF‐300‐AR (evac.) and COF‐300‐NH_2_ (evac.) are provided in this work.

To assess the structural integrity of the newly functionalized COFs, SEM images and PXRD data were collected for each material. The SEM images show the same elliptical particle shape, characteristic of COF‐300, across the entire family of materials (**Figure** **4**a–j). The unchanging morphology throughout the series of linkage transformations bodes well for the maintenance of the framework structure. The PXRD data for the eight new COF‐300 derivatives is shown in Figure 4k. As clearly seen from the numerous sharp peaks in all the PXRD patterns, the COFs remain crystalline after the linkage transformations. While subtle shifts in peak positions are evident from framework to framework, the notable similarity of the PXRD patterns to each other and to that of COF‐300^[^ ^2^, ^16^ ^]^ (Figure 4l, black) indicates that the framework structure remains unchanged throughout the linkage functionalization reactions. Thermogravimetric analysis (Figures S18–S27, Supporting Information) of the functionalized COFs shows that they remained thermally stable up to ≈250 °C.

To better establish the crystal structures of the new materials, we undertook to solve the structure of COF‐300‐NH_2_ as a representative framework. Although the crystallite sizes are on the order of ≈1 um for these materials, making them too small for single‐crystal X‐ray diffraction,^[^ ^40^, ^41^, ^42^ ^]^ this size range is ideally suited for microcrystal electron diffraction (microED, also termed 3D ED).^[^ ^43^, ^44^, ^45^, ^46^, ^47^ ^]^ We obtained microED data for COF‐300‐AR and COF‐300‐NH_2_ that proved sufficient to readily solve the crystal structures of these frameworks. We emphasize the utility of the microED technique for this class of materials; in our standard COF‐300 synthesis, which we are able to run on the ≈1 g scale, the particle size of the products is sufficient to allow structure solution through microED. This represents a major advantage over the rigors necessary to produce COF crystals suitable for single‐crystal X‐ray diffraction.

**Figure 5:**
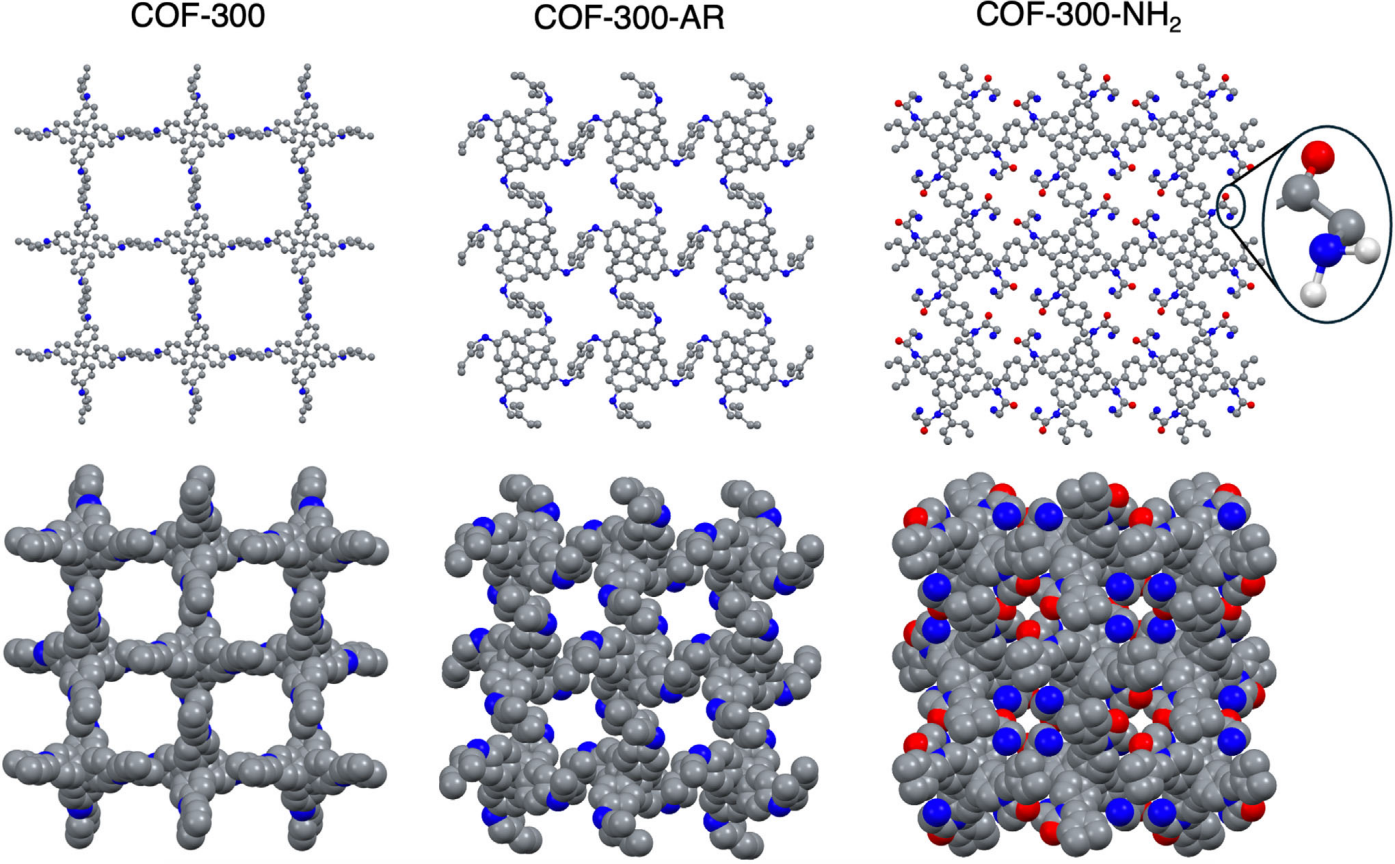
Microcrystal electron diffraction (microED) structures of COF‐300‐AR (center) and COF‐300‐NH_2_ (right) under vacuum, along with the single‐crystal X‐ray diffraction (SCXRD) structure of COF‐300 under vacuum (ref.[16]; left) for comparison. Upper: ball and stick models. Lower: space‐filling models.

The structures obtained through microED for COF‐300‐AR and COF‐300‐NH_2_ are shown in **Figure** **5**, along with the structure of COF‐300^[^ ^16^ ^]^ for comparison. The datasets for COF‐300‐AR and COF‐300‐NH_2_ were collected on activated samples under dynamic vacuum. The parent material, COF‐300, is known to undergo a guest‐induced phase change: When the pores are evacuated, COF‐300 adopts the expanded structure shown in Figure 5, while the adsorption of water triggers a structural contraction in which the pore walls pull inward to engage in H‐bonding with the adsorbed water.^[^ ^16^, ^48^, ^49^, ^50^ ^]^This phase change is evident in the simulated PXRD patterns for evacuated and hydrated COF‐300 (Figure 4l, black and gray, respectively). The first peak shifts from 6.7° to 8.6°, providing a diagnostic PXRD signal to differentiate between the expanded and contracted phases of COF‐300.

Likewise, our prior PXRD studies had suggested a possible phase change between air‐dried and solvated samples of COF‐300‐AR. However, various spectroscopic studies,^[^ ^51^, ^52^ ^]^ including our own,^[^ ^53^ ^]^ indicated that COF‐300 undergoes more significant structural changes in response to changing temperature than does COF‐300‐AR, suggesting that COF‐300 is more “flexible” than COF‐300‐AR. There has been some degree of consternation in the literature about the apparently greater flexibility of COF‐300, as the C═N linkages in COF‐300 should prevent free rotation about the *π* bond while the C─N linkages in COF‐300‐AR should allow for greater rotation and flexibility. A further source of puzzlement has been the loss of N_2_‐accessible porosity upon the reduction of COF‐300 to COF‐300‐AR, documented by us^[^ ^38^ ^]^ and others;^[^ ^54^ ^]^ we found the BET surface area of COF‐300‐AR to be only 13 m^2^ g^−1^, less than 1% of the BET surface area of COF‐300 (1383 m^2^ g^−1^). The crystal structure of evacuated COF‐300‐AR, shown in Figure 5 can finally settle these questions. Upon removal of guest molecules from the pores, COF‐300‐AR collapses inward to maximize stabilizing intra‐framework interactions, while the overall framework connectivity is maintained. It is interesting to note that this collapse upon evacuation is not possible for COF‐300, perhaps because of its less‐flexible C═N linkages. These results explain the spectroscopic observations^[^ ^53^ ^]^ that evacuated COF‐300 undergoes a greater structural change upon cryogenic cooling than does evacuated COF‐300‐AR: Since evacuated COF‐300‐AR is already collapsed at room temperature, little room remains for a further structural contraction upon cryogenic cooling.

Thus, COF‐300‐AR shows opposite phase‐change behavior to COF‐300. The parent framework COF‐300 maintains its expanded structure under vacuum but contracts when a strongly‐binding solvent enters the pores. In contrast, COF‐300‐AR adopts an expanded phase when the pores are solvated, as we have shown through PXRD studies (Figure 4l, purple) and as seen in a recently published single‐crystal structure of solvated COF‐300‐AR,^[^ ^39^ ^]^ while the evacuated structure of COF‐300‐AR reported here shows the pores to be significantly contracted. Specifically, evacuated COF‐300‐AR crystallizes in the space group *I*4_1_/a with unit‐cell parameters of *a* = *b* = 20.352(5) Å and *c* = 7.3547(17) Å and a unit cell volume of 3046.4(16) Å^3^. This unit cell volume is dramatically smaller than that of solvated COF‐300‐AR (5429.7 Å^3^),^[^ ^39^ ^]^ highlighting the extent of the solvent‐induced phase change in this framework.

Evacuated COF‐300‐NH_2_ crystallizes in the same space group (*I*4_1_/a) with unit cell parameters of *a* = *b* = 24.370(13) Å and *c* = 7.002(17) Å and a larger unit cell volume of 4159(5) Å^3^ compared to evacuated COF‐300‐AR. The alkyl amine groups extend into the pores, forming cross‐pore H‐bonding interactions between carbonyl H‐bond acceptors and –NH_2_ H‐bond donors with O⋅⋅⋅H distances of 2.49 Å. These stabilizing intra‐framework interactions, along with the steric bulk of the amino‐carbonyl functional group, occlude much of the pore space. The space‐filling model of COF‐300‐NH_2_ emphasizes this crowded pore environment (Figure 5, lower right). Unlike for COF‐300‐AR, the addition of solvent does not cause a significant change in PXRD peak position (Figure 4l, green), suggesting a higher barrier for the expansive phase change for COF‐300‐NH_2_ than for COF‐300‐AR. However, aqueous adsorption experiments and CO_2_ adsorption isotherms show that COF‐300‐NH_2_ retains a high adsorption capacity, as discussed below.

**Figure 6:**
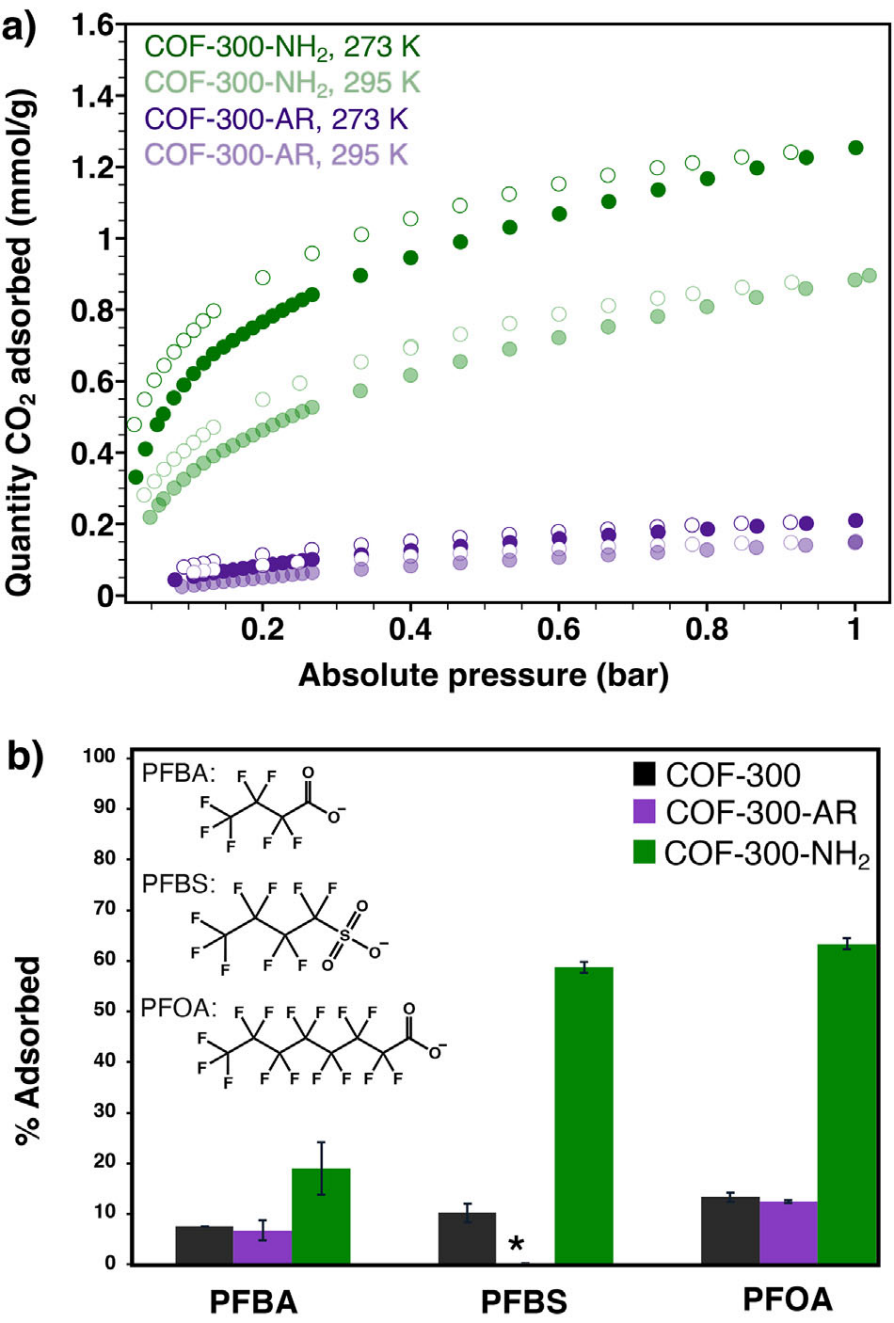
a) CO_2_ sorption isotherms for COF‐300‐NH_2_ at 273 K (dark green) and 295 K (light green) and for COF‐300‐AR at 273 K (dark purple) and 295 K (light purple). Filled circles are adsorption, and open circles are desorption. b) Perfluoroalkyl substance (PFAS) adsorption measurements for COF‐300 (black), COF‐300‐AR (purple), and COF‐300‐NH_2_ (green). ^*^PFBS adsorption for COF‐300‐AR was within an error of 0%.

Like COF‐300‐AR, COF‐300‐Ac‐Br and COF‐300‐NH_2_ show minimal BET surface areas (9 and 15 m^2^ g^−1^, respectively; Figures S29 and S30, Supporting Information). We hypothesize that upon evacuation, the flexible C–N linkages in these materials allow the structures to contract and form stabilizing intra‐framework interactions, as illustrated by the evacuated crystal structures of COF‐300‐AR and COF‐300‐NH_2_ (Figure 5). Given this trend, BET surface areas were not measured for the six other COF‐300 derivatives reported here. However, to determine whether the low N_2_ adsorption capacity of COF‐300‐NH_2_ indicates non‐porosity to other adsorbates, we collected CO_2_ sorption isotherms at 273 and 295 K (**Figure** **6**a). The results show a considerable increase in CO_2_ capacity for COF‐300‐NH_2_ compared to COF‐300‐AR. At 273 K, COF‐300‐NH_2_ reaches a CO_2_ capacity of 1.3 mmol g^−1^ at 1 bar, compared to only 0.2 mmol g^−1^ for COF‐300‐AR. While improved CO_2_ adsorption is unsurprising for a framework bristling with primary amines, the impressive CO_2_ adsorption results for COF‐300‐NH_2_ emphasize that this framework is far from non‐porous. Instead, the microED, PXRD, BET, and CO_2_ adsorption results together suggest that while N_2_ gas interacts too weakly with COF‐300‐NH_2_ to overcome the intra‐framework interactions stabilizing the collapsed phase, the framework does exhibit significant porosity under conditions such as CO_2_ adsorption at 273 K.

We found further evidence of porosity in COF‐300‐NH_2_ from PFAS adsorption studies. Because the alkyl amine groups of COF‐300‐NH_2_ should be protonated in neutral water, forming –NH_3_^+^ cations, we hypothesized that this framework could serve as an ion adsorbent for anionic PFAS molecules.^[^ ^55^, ^56^ ^]^ We selected three widespread PFAS contaminants for our study: perfluorooctanoic acid (PFOA), perfluorobutanoic acid (PFBA), and perfluorobutanesulfonic acid (PFBS).^[^ ^57^, ^58^, ^59^ ^]^ When submerged in 100 ppb solutions of the respective PFAS, COF‐300‐NH_2_ adsorbed 63% of PFOA, 59% of PFBS, and 19% of PFBA, representing a significant improvement in PFAS adsorption capacity over the precursor materials COF‐300 and COF‐300‐AR (Figure 6b). Thus, the adsorption performance of COF‐300‐NH_2_ for PFAS and CO_2_ guests illustrates the power of the synthetic strategy offered here. In a post‐synthetic linkage transformation that preserves the overall framework architecture of COF‐300, chemists can install functional groups tailored to desired applications.

## 3 Conclusion

We have demonstrated a synthetic approach that allows the recalcitrant framework COF‐300 to undergo facile and wide‐ranging functionalization. There is long‐standing recognition of the benefits of functionalization for framework materials and for COF‐300 in particular. For example, Lu and coworkers published a theoretical study in which they modeled COF‐300 derivatives bearing –NH_2_, –OH, –NO_2_, and –SO_3_ groups and found that such functionalization should enhance gas separation performance.^[^ ^60^ ^]^ Meanwhile, synthetic chemists (ourselves included) have spent years trying and failing to synthesize such COF‐300 derivatives through monomer functionalization. Here, we have overcome this synthetic bottleneck by addressing the linkages, rather than the monomers, as sites for functionalization. By appending electrophilic groups to the COF‐300 linkages, we were able to produce N_3_‐, CN‐, NH_2_‐, OH‐, OCH_3_‐, and SCH_3_‐functionalized derivatives through facile S_N_2 reactions.

Our demonstration of improved CO_2_ and PFAS adsorption for COF‐300‐NH_2_ is simply an example of the possible benefits of these diverse functional groups. We hypothesize that the sulfur‐enriched material, COF‐300‐SMe, may show improved adsorption of heavy metal ions, and that the azide‐appended material, COF‐300‐N_3_, may prove amenable to azide‐alkyne click chemistry, among many possible uses of this family of materials. Further, PXRD and microED studies clearly illustrate the adsorbate‐dependent phase change that occurs for COF‐300‐AR but not for COF‐300‐NH_2_. This insight opens the door to the modulation of phase‐change behavior through linkage functionalization, which can offer major advantages in adsorptive separations. In summary, the late‐stage diversification of COF‐300 demonstrated here can allow chemists to tailor this hallmark COF to a broad range of real‐world applications.

## Conflict of Interest

The authors declare no conflict of interest.

## Supporting information



Supporting Information

Supplemental Video 1

Supplemental Video 2

## Data Availability

The data that support the findings of this study are available from the corresponding author upon reasonable request.
