# Supplementary material for: Tailoring a 3D Covalent Organic Framework Toward Facile Functionalization
Source: Small. 2025 Dec 8;22(3):e11087. doi: 10.1002/smll.202511087 (PMC12802530; doi:10.1002/smll.202511087)
Supplement: Supplementary file 1 — Supporting Information [file SMLL-22-e11087-s003.pdf]

NANO · MICRO
small

Supporting Information

for *Small*, DOI 10.1002/smll.202511087

# Tailoring a 3D Covalent Organic Framework Toward Facile Functionalization

*Silas O. Frimpong, Maxima Pacheco, Haomiao Xie, Andrea N. Zeppuhar, Omar K. Farha and Mercedes K. Taylor**

# Supporting Information

# Tailoring a Three-Dimensional Covalent Organic Framework towards Facile Functionalization

*Silas O. Frimpong,[a] Maxima Pacheco,[a] Haomiao Xie,[b] Andrea Zeppuhar,[a] Omar K. Farha, [b] and Mercedes K. Taylor*[a]*

Table of Contents

Materials ........................................ S2
NMR Techniques ........................................ S2
Powder X-Ray Diffraction (PXRD) ........................................ S2
Microcrystal Electron Diffraction (microED) ........................................ S2
Gas Sorption Measurements ........................................ S3
Scanning Electron Microscopy (SEM) Imaging ........................................ S3
Transmission Electron Microscopy (TEM) Imaging ........................................ S4
Energy Dispersive X-Ray Spectroscopy (EDX) ........................................ S4
Thermogravimetric Analysis (TGA) ........................................ S4
Fourier Transform Infrared (FTIR) Spectroscopy ........................................ S4
Mass Spectrometry ........................................ S4
Perfluoroalkyl Substance (PFAS) Adsorption Experiments ........................................ S4
Monomer Synthesis ........................................ S6
Model Compounds Synthesis ........................................ S8
Covalent Organic Frameworks (COFs) Synthesis ........................................ S14
Supplementary Figures ........................................ S24
ORTEP-style illustrations and A- and/or B-level Alerts Justifications ........................................ S53
Supplementary Tables ........................................ S57
References ........................................ S60

**General Methods:**

**Materials:** Anhydrous tetrahydrofuran (THF) and anhydrous *N*,*N*-dimethylformamide (DMF) were purified using an Inert Pure Solv solvent purification system. All other chemicals and solvents were obtained from Sigma-Aldrich, ThermoFisher, and TCI and were used without further purification. Nylon filter paper with pore size of 0.2 μm was obtained from Cole-Parmer.

**Nuclear Magnetic Resonance (NMR) Spectroscopy**: Solution-state $^{1}H$ and $^{13}C$ NMR spectra were collected on a Bruker 400 MHz instrument. Chemical shifts are reported in parts per million (ppm) and are referenced to the solvent peaks $CDCl_3$ or DMSO-$d_6$. High-resolution solid-state nuclear magnetic resonance (ssNMR) spectra were collected on a Bruker Avance NEO 500 MHz spectrometer with a double resonance HX probe. Cross-polarization (CP) with magic-angle spinning (MAS) was used to acquire the $^{13}C$ and $^{15}N$ spectra. Powder samples were packed in a 3.2 mm regular wall zirconia MAS rotor with an SP1 drive cap. The magic angle spinning rate was 16.5 kHz. Proton-carbon and proton-nitrogen matched cross polarization ramp was set to 50 kHz with a 2 ms contact time. Proton dipolar decoupling was achieved by applying continuous wave small phase incremental alternation with 64 steps (SPINAL-64) on the $^{1}H$ channel during acquisition. The 90-degree pulse length was 2.5 μs for $^{1}H$ and the recycle delay was 5 s. The $^{13}C$ and $^{15}N$ chemical shifts are given relative to tetramethylsilane as zero ppm, using the methylene carbon of adamantane assigned to 38.2 and 28.7 ppm as references. Each solid-state $^{13}C$ spectrum was collected with 2k scans, while $^{15}N$ was collected with 105k scans.

**Powder X-ray Diffraction (PXRD):** PXRD patterns of covalent organic framework (COF) samples were obtained using a Bruker D8 Advance diffractometer. The powder was placed on a sample holder and exposed to X-rays with a Cu Kα source ($\lambda = 1.5406$ Å, 40 kV, 40 mA). The PXRD patterns for the COFs were recorded from 5° to 50° (2θ) with a step of 0.02° under ambient air conditions. Brucker's DIFFRAC.EVA V5.2 was used to export background subtracted powder pattern for analysis.

**Microcrystal electron diffraction (microED):** MicroED data for the COFs were collected at 298 K under vacuum using a MicroED setup (XtaLAB Synergy-ED, Rigaku), equipped with a Rigaku HyPix-ED direct electron detector and operated via the Rigaku Oxford Diffraction CrysAlisPro

software suite. The system features a 200 kV electron gun with a lanthanum hexaboride (LaB6) emitter to optimize electron optics. Structure determination was carried out by intrinsic phasing using SHELXL (2019/3), followed by full-matrix least-squares refinement with SHELXL (2019/3) within the Olex2 interface. Disordered, non-coordinated solvent molecules were removed using the solvent mask function in Olex2. Refinement details are provided in Supplementary Table S1, and crystallographic information files (cifs) have been deposited in the Cambridge Crystallographic Data Centre (CCDC) under the deposition numbers listed in Supplementary Table S1.

**Gas Sorption Measurements:** Adsorption isotherms for $N_2$ and $CO_2$ were obtained using a Micromeritics ASAP 2020 Plus adsorption analyzer, adapted from literature procedures.[1] An oven-dried analysis tube with a glass filler rod was evacuated to a stable pressure of 9 μbar on the analyzer, and the mass of the evacuated tube and rod was recorded. About 120 mg of the sample was weighed in air and transferred to the analysis tube, which was then activated at 90 °C under an inert atmosphere overnight on a Schlenk line. The sample with the rod was further activated on the adsorption analyzer at 90 °C under vacuum overnight. After activation, the tube's mass was measured again to determine the precise mass of the activated sample. The analysis tube with the rod and sample was then connected to the instrument for surface area and gas adsorption measurements. A liquid nitrogen bath (77 K), an ice bath (273 K), and a water bath (295 K) were used for $N_2$ adsorption at 77 K, $CO_2$ adsorption at 273 K, and $N_2$ and $CO_2$ adsorption at 295 K, respectively. The Brunauer-Emmett-Teller (BET) method was used to determine the specific surface area ($m^2/g$). The isosteric heat of $CO_2$ adsorption ($Q_{st}$) was obtained using the Clausius-Clapeyron equation below with two isotherms at 273 and 295 K. [1]

$$Q_{st} = -R \frac{\ln(p_2/p_1)}{(1/T_2 - 1/T_1)}$$

**Scanning Electron Microscopy (SEM) Imaging:** SEM images were taken using a Hitachi SU-70 FEG instrument. Dry powder samples were placed on carbon tape attached to a sample holder. The samples were then coated with gold/palladium for 10 minutes to prevent charging during image collection.

**Transmission Electron Microscopy (TEM) Imaging:** Bright field transmission electron microscopy images were collected with JEOL JEM 2100 $LaB_6$ TEM operated at 200 kv in conventional bright-field mode. In a 2 mL vial, COF samples were dispersed in ethanol and shaken for about 20 minutes to obtain fine colloidal particles. After sitting for about 2 minutes, two drops of the suspension was drop-cast onto the grid and allow to air dry. Using a single tube holder, prepared sample was inserted into the instrument for data collection. Images were collected with Gatan camera.

**Energy-dispersive X-ray (EDX) Spectroscopy:** EDS measurements were performed using an EDAX Element EDS system integrated into a Tescan XEIA FEG scanning electron microscope. The focused ion beam (FIB) technique was carried out with a Xe plasma FIB column in combination with the instrument's Schottky field emission cathode.

**Thermogravimetric Analysis (TGA):** Thermogravimetric analysis (TGA) was performed on a Discovery TGA-55000. Samples (5-10 mg) were heated in either a platinum or platinum HT pan from room temperature to 800 °C at a rate of 5 °C/min under nitrogen flow (25 mL/min).

**Fourier Transform Infrared (FTIR) Spectroscopy:** Infrared (IR) spectra were collected using a Thermo Nicolet Nexus 670 FTIR with an ATR probe. Data were acquired from powdered samples under ambient conditions. The surrounding air was used as the blank.

**Mass Spectrometry**: MS (DART) Mass was performed using a JEOL AccuTOF electrospray instrument (ESI). Quantification of PFAS adsorption was measured using a Bruker Maxis-II Q-TOF mass spectrometer equipped with an ESI ion source coupled with a Waters Acquity I-Class Plus LC system.

**Perfluoroalkyl Substance (PFAS) Adsorption Experiments:** COF samples were activated under vacuum at 120 °C for 24 hours. COF samples (10 mg) were measured into tared 15 mL centrifuge tubes. A 100 ppb PFAS solution (10 g) was added to achieve a concentration of 1 mg COF per 1 gram of PFAS solution. The adsorption mixtures were then shaken at 80 rpm for 18 hours, after which the mixture was passed through a 0.2 µm PES syringe filter and analyzed using LC-MS.

Control experiments were performed in which an identical PFAS solution was shaken for 18 hours in the absence of COF and was then passed through a 0.2 μm PES syringe filter and analyzed via LC-MS. The PFAS concentration measured in these COF-free control experiments provided the starting PFAS concentration for the COF-containing experiments. Batch adsorption experiments were performed in triplicate.

Time-dependent adsorption experiments for PFBS were carried out in the same way while removing aliquots at the following time intervals: 1, 3, 5, 10, 15, 20, 30, 60, 120, 180, 240, 360, 1260 and 1440 mins. The formula for quantifying PFBS adsorbed is given below,

$$q_t\ (\mu\text{mol g}^{-1}) = \frac{m_o(1\text{-}A_t/A_o)}{m_{\text{COF}}} \times \frac{1000}{MW}$$

where $q_t$ is adsorption capacity at time $t$, $m_o$ is initial mass of PFBS added to the solution, $m_{\text{COF}}$ is mass of adsorbent, $A_t$ is signal intensity proportional to PFBS concentration at time $t$, $A_o$ is signal intensity proportional to initial PFBS concentration before adsorption, $MW$ is molecular weight of PFBS. The experimental results from the PFBS kinetics was fitted with pseudo-first-order and pseudo-second-order model with the following formulae,

| Pseudo 1st Order | Pseudo 2nd Order |
|---|---|
| $\ln(q_e - q_t) = \ln(q_e) - k_1 t$ | $\frac{t}{q_t} = \frac{1}{k_2 q_e^2} + \frac{t}{q_e}$ |

where $q_e$ is equilibrium uptake, $q_t$ is uptake at time = t and $k$ is the rate of adsorption. The parameters for each rate model are given in Table S5.

Cycling studies for PFBS adsorption onto COF-300-$NH_2$ were performed according to a similar procedure, as follows: The COF samples (10 mg) were measured into tared 15 mL centrifuge tubes. A 100 ppb PFBS solution (10 g) was added to achieve a concentration of 1 mg COF per 1 gram of PFAS solution. The adsorption mixtures were shaken at 80 rpm for 24 hours, after which a portion of the mixture was passed through a 0.2 μm PES syringe filter and analyzed using LC-MS. Control experiments were performed in which an identical PFAS solution was shaken for 24 hours in the absence of COF and was then passed through a 0.2 μm PES syringe filter and analyzed via LC-MS. The PFAS concentration measured in these COF-free control experiments provided the starting PFAS concentration for the COF-containing experiments. After completion of the first PFBS adsorption experiment, the COF-300-$NH_2$ sample was recovered via filtration, regenerated through overnight soaking in ethanol, and activated at 120 °C.[2] The process

was then repeated twice more for the same COF-300-$NH_2$ sample, resulting in a total of three adsorption/regeneration cycles.

## Monomer Synthesis

### Synthesis of tetraphenylmethane

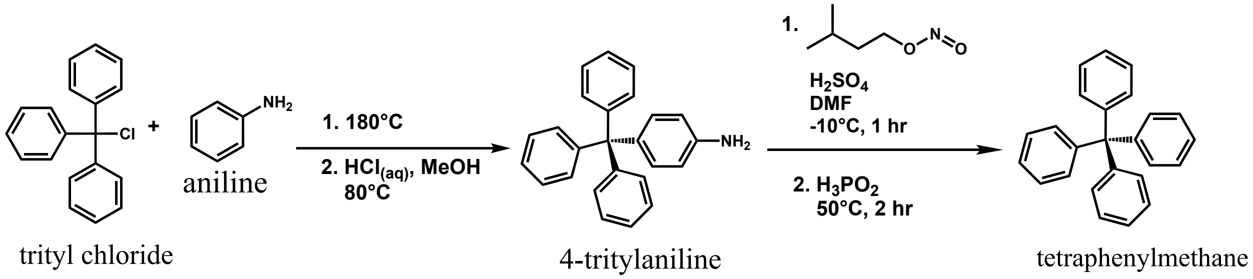


The synthesis of tetraphenylmethane was adapted from a known procedure.[3,4] Trityl chloride (10.00 g, 35.90 mmol) and aniline (9.4 mL, 104.1 mmol) were added to a 500 mL round-bottom flask (RBF) equipped with a stir bar. The mixture was refluxed at 180 °C with vigorous stirring until a violet solid formed. The heating was continued for an additional 10 minutes, after which the reaction was cooled to room temperature (r.t.). The solids were crushed into smaller pieces and suspended in a 1:1 mixture of methanol and 2 M aqueous HCl (50 mL each). The violet suspension was further refluxed at 80 °C for 30 minutes. It was then cooled to r.t. and vacuum-filtered, followed by washing with water, to obtain 4-tritylaniline as a light purple residue. The solid was dried under vacuum at 70 °C overnight. In a 250 mL RBF, the dried 4-tritylaniline was suspended in 105.6 mL of DMF and stirred. The mixture was cooled to -10 °C using an acetone/ice bath. Concentrated $H_2SO_4$ (18 M in water, 11.4 mL, 204.5 mmol) was added slowly, followed by isopentyl nitrite (8.6 mL, 64.6 mmol). After stirring at -10 °C for an hour, phosphinic acid (50% v/v, 15.4 mL, 140.6 mmol) was added dropwise. A brown cloudy mixture formed, which was warmed to room temperature and further refluxed at 50 °C under stirring for 2 hours. After bringing the reaction to room temperature, it was vacuum filtered and washed with ~50 mL DMF (twice), ~100 mL water, and ~50 mL ethanol. The product was dried under vacuum, yielding tetraphenylmethane as a brown solid. Yield: 9.90 g (86%). $^1H$ NMR (400 MHz, $CDCl_3$) $\delta$ = 7.27-7.18 (m, 20H).

**Synthesis of tetrakis(4-nitrophenyl) methane**

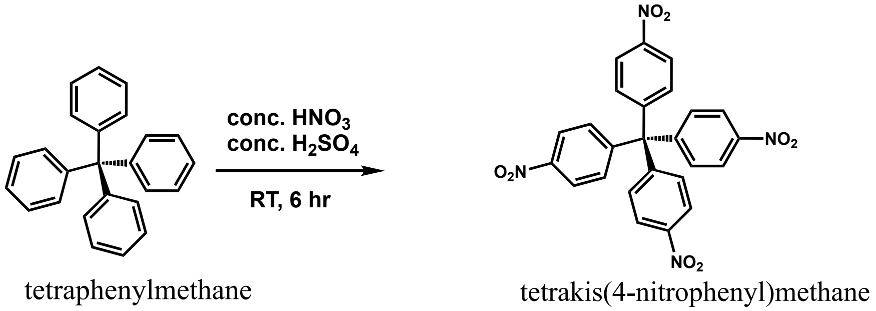


The synthesis of tetrakis(4-nitrophenyl)methane was adapted from a previous procedure.[3–5] Tetraphenylmethane (9.9 g, 30.9 mmol) was transferred to a 250 mL round-bottom flask in an ice bath equipped with a stir bar. In a separate 250 mL Erlenmeyer flask, also cooled to 0 °C, a mixture of 15.7 M $HNO_3$ (41.1 mL, 624.2 mmol) and 18 M $H_2SO_4$ (19.9 mL, 358.4 mmol) was prepared. The acid mixture was carefully added dropwise to the tetraphenylmethane at 0 °C. The reaction mixture was then brought to room temperature and stirred for 6 hours. Afterwards, it was placed in an ice bath and diluted with approximately 35 mL of deionized water. Using a fine porous fritted funnel, the mixture was vacuum filtered and washed extensively with water. The resulting solid was recrystallized in ~300 mL THF, yielding 6.2 g (40%) of tetrakis(4-nitrophenyl)methane. $^1$H NMR (400 MHz, $CDCl_3$) δ = 7.43-7.41 (d, *J* = 8.8 Hz, 8H); 8.24-8.23 (d, *J* = 9 Hz, 8H).

**Synthesis of tetrakis(4-aminophenyl)methane (TAPM)**

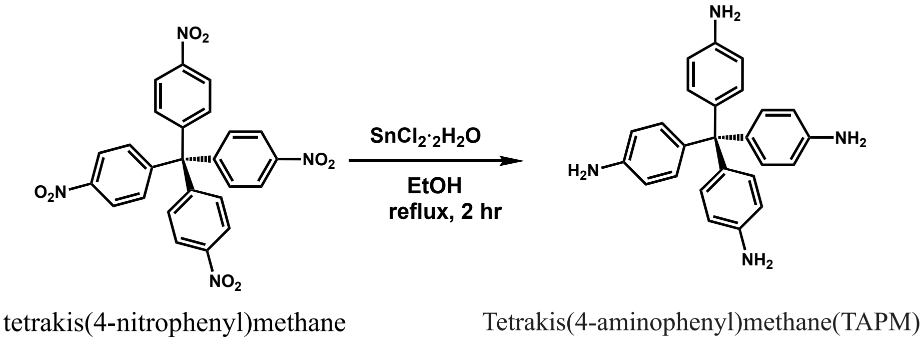


The synthesis of tetrakis(4-aminophenyl)methane was adapted from a reported procedure.[5–7] Tetrakis(4-nitrophenyl)methane (6.2 g, 12.4 mmol) was dissolved in 618 mL of ethanol in a 1 L round-bottom flask. Tin chloride dihydrate (55.8 g, 247.2 mmol) was added to the reaction

mixture, which was refluxed at 90 °C for 2 hours. After bringing the mixture to room temperature, ethanol was removed under reduced pressure. The mixture was then diluted with approximately 200 mL of water and cooled to 0 °C. Subsequently, 10% aqueous sodium hydroxide (w/w) was added until the pH reached approximately 10. The mixture was returned to room temperature and allowed to stir overnight to hydrolyze all tin salts. The resulting mixture was vacuum filtered and washed with ~200 mL water. The solids were heated to 80 °C in about 300 mL of 1,4-dioxane to selectively dissolve tetrakis(4-aminophenyl)methane. The hot mixture was vacuum filtered to remove solids, which were then washed with ~80 mL hot 1,4-dioxane to ensure complete collection of TAPM in the filtrate. The 1,4-dioxane was then removed under reduced pressure. Water (~100 mL) was added to the remaining mixture, producing a brownish product that was vacuum filtered. The solids were washed with a large volume of water (~500) mL, followed by an ethanol (~40 mL) wash, to finally obtain tetrakis(4-aminophenyl)methane as a beige solid. Yield: 3.7 g (80%). 1H NMR (400 MHz, DMSO-$d_6$) δ = 4.85 (s, 8H), 6.39-6.37 (d, $J$ = 8.4 Hz, 8H), 6.67–6.66 (d, $J$ = 8.7 Hz, 8H).

## Synthesis of model compounds

### ***N*-benzyl-2-chloro-*N*-phenylacetamide**

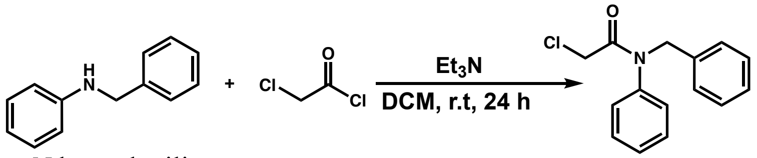


*N*-benzylaniline *N*-benzyl-2-chloro-*N*-phenylacetamide

In an oven-dried 100 mL, 2-neck round-bottom flask under flowing argon, *N*-benzylaniline (1.000 g, 5.460 mmol), anhydrous dichloromethane (36.4 mL, 0.15 M), and triethylamine (0.8 mL, 5.460 mmol) were combined. The resulting mixture was stirred and cooled at 0 °C for 15 minutes. Chloroacetyl chloride (0.6 mL, 8.190 mmol) was then added dropwise. The reaction mixture was warmed to room temperature (r.t) and stirred for 2 hours. The product was extracted by diluting with ~20 mL dichloromethane and washing with water (~20 mL), aqueous sodium bicarbonate (~20 mL), and brine (~20 mL) in a separatory funnel. The organic layer was dried over anhydrous

magnesium sulfate, and the solvent was removed under reduced pressure to yield *N*-benzyl-2-chloro-*N*-phenylacetamide (1.020 g, 72%). 1H-NMR (400 MHz, $CDCl_3$, δ): δ ppm: 3.86 (s, 2H), 4.91 (s, 2H), 7.01-7.04 (m, 2H), 7.19-7.22 (m, 2H), 7.25-7.30 (m, 3H), 7.35-7.38 (m, 3H).

**_N_-benzyl-2-bromo-_N_-phenylacetamide**

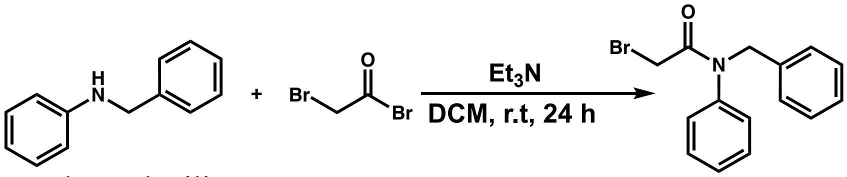

*N*-benzylaniline *N*-benzyl-2-bromo-*N*-phenylacetamide

In an oven-dried 100 mL two-neck round-bottom flask under flowing argon, *N*-benzylaniline (2.000 g, 10.91 mmol), anhydrous dichloromethane (73 mL, 0.15 M), and triethylamine (1.4 mL, 10.19 mmol) were combined. The mixture was stirred and cooled to 0 °C for 15 minutes. Bromoacetyl bromide (1.2 mL, 14.19 mmol) was then added dropwise. The reaction mixture was brought to room temperature and stirred for 24 hours. The product was extracted by diluting with 40 mL dichloromethane and washing with water (40 mL), aqueous sodium bicarbonate (40 mL), and brine (40 mL) in a separatory funnel. The organic layer was dried over anhydrous magnesium sulfate, and the solvent was removed under reduced pressure to yield *N*-benzyl-2-bromo-*N*-phenylacetamide as a viscous brown liquid (2.300 g, 70%). $^1$H-NMR (400 MHz, $CDCl_3$, δ): δ ppm: 3.65 (s, 2H), 4.89 (s, 2H), 7.05 (m, 2H), 7.18 (m, 2H), 7.26 (m, 3H), 7.34 (m, 3H).

**2-azido-_N_-benzyl-_N_-phenylacetamide**

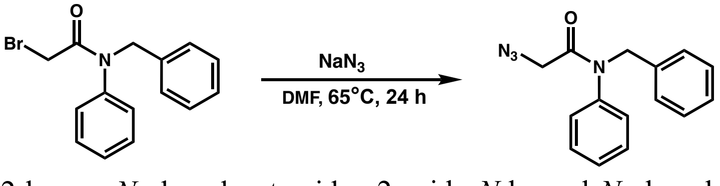

*N*-benzyl-2-bromo-*N*-phenylacetamide 2-azido-*N*-benzyl-*N*-phenylacetamide

In an oven-dried 100 mL, 2-neck round-bottom flask under flowing argon, *N*-benzyl-2-bromo-*N*-phenylacetamide (310 mg, 1.440 mmol), anhydrous DMF (20 mL, 0.072 M), and sodium azide (187 mg, 2.880 mmol) were combined at room temperature. The reaction mixture was then

refluxed and stirred at 65 °C for 24 hours. The product was extracted by diluting with 20 mL dichloromethane and washing sequentially with water (20 mL), aqueous sodium bicarbonate (20 mL), and brine (20 mL) in a separatory funnel. The organic layer was dried over anhydrous magnesium sulfate, and the solvent was removed under reduced pressure to yield 2-azido-*N*-benzyl-*N*-phenylacetamide as a brown viscous liquid (336 mg, 96%). The $^1$H-NMR spectrum (400 MHz, $CDCl_3$, δ): δ ppm: 3.59 (s, 2H), 4.90 (s, 2H), 6.96 (m, 2H), 7.19 (m, 2H), 7.26 (m, 3H), 7.35 (m, 3H). The $^{13}$C-NMR spectrum (400 MHz, CDCl3, δ): 51.02, 53.56, 53.56,127.85, 128.35, 128.64, 128.92, 129.18, 130.09, 136.73, 140.47, 167.49.

**N-benzyl-2-cyano-N-phenylacetamide**

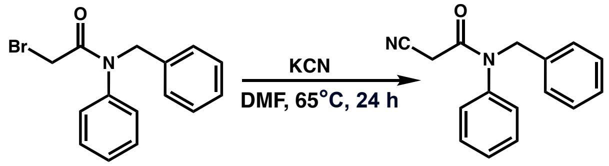


*N*-benzyl-2-bromo-*N*-phenylacetamide *N*-benzyl-2-cyano-*N*-phenylacetamide

In an oven-dried 100 mL 2-neck round-bottom flask under flowing argon, *N*-benzyl-2-bromo-*N*-phenylacetamide (200 mg, 0.770 mmol), anhydrous DMF (30 mL, 0.025 M), and potassium cyanide (100 mg, 1.540 mmol) were combined at room temperature. The reaction mixture was then refluxed and stirred at 65 °C for 24 hours. The product was extracted by diluting it with 20 mL of dichloromethane and washing with water (20 mL), aqueous sodium bicarbonate (20 mL), and brine (20 mL) in a separatory funnel. The organic layer was dried over anhydrous magnesium sulfate, and the solvent was removed under reduced pressure to yield *N*-benzyl-2-cyano-*N*-phenylacetamide as a pale-brown viscous liquid (180 mg, 93%). $^1$H-NMR (400 MHz, $CDCl_3$, δ): 3.21 (s, 2H), 4.89 (s, 2H), 6.99 (m, 2H), 7.18 (m, 2H), 7.28 (m, 3H), 7.39 (m, 3H). $^{13}$C-NMR (400 MHz, CDCl3, δ): 25.95, 54.05, 54.05,128.05, 128.34, 128.70, 129.22, 129.33, 130.37, 131.18, 131.18,132.48, 136.21, 140.71.

**2-amino-*N*-benzyl-*N*-phenylacetamide**

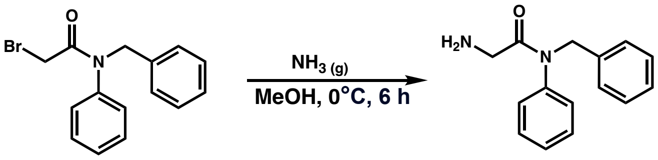

*N*-benzyl-2-bromo-*N*-phenylacetamide 2-amino-*N*-benzyl-*N*-phenylacetamide

In a 100 mL round-bottom flask, *N*-benzyl-2-bromo-*N*-phenylacetamide (166 mg, 0.600 mmol) and anhydrous THF (10 mL) were combined at room temperature. The reaction mixture was cooled to 0 °C. Excess ammonia gas was bubbled through the reaction mixture at 0 °C for 6 hours. The ammonia gas was generated in situ from reacting ammonium hydroxide (28-30%, 300 mL), which was released dropwise onto sodium hydroxide pellets (20 g) in another flask. The setup is shown in Figure S44. The reaction mixture was brought back to room temperature, and the mixture was diluted with ethyl acetate and washed with water (20 mL) and brine (20 mL) in a separatory funnel. The organic layer was dried over anhydrous magnesium sulfate, and the solvent was removed under reduced pressure to yield 2-amino-*N*-benzyl-*N*-phenylacetamide as a pale-yellow viscous liquid (111 mg, 77%). MS (DART), *m/z* calculated for $C_{15}H_{16}N_2O$: 241.13 $[M+H]^+$; found: 241.12 $[M+H]^+$.

***N*-benzyl-2-hydroxy-*N*-phenylacetamide**

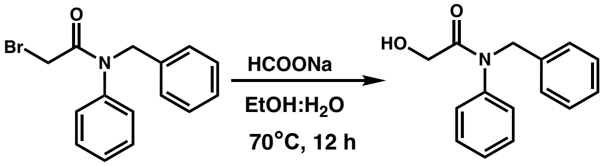

*N*-benzyl-2-bromo-*N*-phenylacetamide *N*-benzyl-2-hydroxy-*N*-phenylacetamide

In a 100 mL round-bottom flask, *N*-benzyl-2-bromo-*N*-phenylacetamide (70 mg, 0.230 mmol), a 2:1 ethanol/water mixture (v/v; 15 mL), and sodium formate (109 mg, 1.600 mmol) were combined at room temperature. The reaction mixture was then refluxed and stirred at 70 °C for 12 hours. Upon completion, the ethanol was removed under vacuum. The crude mixture was taken up in ~20 mL ethyl acetate and washed with water (20 mL), aqueous sodium bicarbonate (20 mL), and brine (20 mL) in a separatory funnel. The organic layer was dried over anhydrous magnesium

sulfate, and the solvent was removed under reduced pressure to yield *N*-benzyl-2-hydroxy-*N*-phenylacetamide as a yellow viscous liquid (50 mg, 90%). $^{1}$H-NMR (400 MHz, $CDCl_3$, δ): δ ppm: 3.79 (s, 2H), 4.91 (s, 2H), 6.96 (m, 2H), 7.19 (m, 2H), 7.27 (m, 3H), 7.35 (m, 3H). $^{13}$C-NMR (400 MHz, CDCl3, δ): 53.77, 60.74, 127.90, 128.44, 128.69, 129.05, 129.08, 130.01, 139.28, 144.02, 160.14.

### *N*-benzyl-2-methoxy-*N*-phenylacetamide

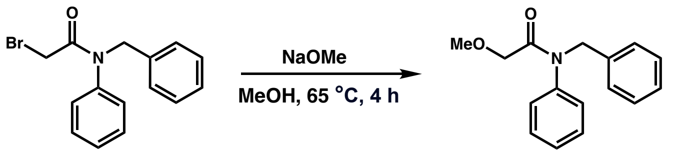


*N*-benzyl-2-bromo-*N*-phenylacetamide *N*-benzyl-2-methoxy-*N*-phenylacetamide

In a 100 mL RBF, *N*-benzyl-2-bromo-*N*-phenylacetamide (98 mg, 0.320 mmol), methanol (15 mL, 0.021 M), and sodium methoxide (82 mg, 1.200 mmol) were combined at room temperature. The reaction mixture was then refluxed and stirred at 65 °C for 4 hours. Afterward, the mixture was cooled to room temperature and diluted with ~30 mL ethyl acetate. The organic layer was then washed with water (20 mL), aqueous sodium bicarbonate (20 mL), and brine (20 mL) in a separatory funnel. The organic layer was dried over anhydrous magnesium sulfate, and the solvent was removed under reduced pressure to obtain *N*-benzyl-2-methoxy-*N*-phenylacetamide as a pale-yellow viscous liquid (74 mg, 91%). $^{1}$H-NMR (400 MHz, $CDCl_3$, δ): δ ppm: 3.36 (s, 3H), 3.77 (s, 2H), 4.89 (s, 2H), 6.96 (m, 2H), 7.20 (m, 2H), 7.25 (m, 3H), 7.33 (m, 3H). $^{13}$C-NMR (400 MHz, CDCl3, δ): 53.18, 59.39, 70.80, 127.64, 128.47, 128.54, 128.58, 129.17, 139.83, 136.73, 140.47, 167.49.

**N-benzyl-2-(methylthio)-N-phenylacetamide**

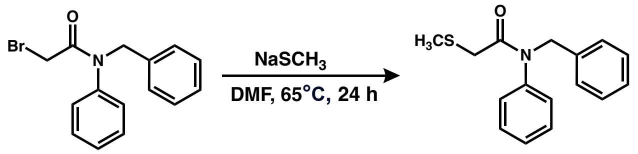


*N*-benzyl-2-bromo-*N*-phenylacetamide *N*-benzyl-2-(methylthio)-*N*-phenylacetamide

In a 100 mL round-bottom flask, *N*-benzyl-2-bromo-*N*-phenylacetamide (375 mg, 1.440 mmol), dimethylformamide, and distilled water (2:1, 15 mL) were combined at room temperature. The mixture was then refluxed and stirred at 65 °C for 24 hours. The reaction product was diluted with ~20 mL dichloromethane and washed with water (20 mL), aqueous sodium bicarbonate (20 mL), and brine (20 mL) in a separatory funnel. The organic layer was dried over anhydrous magnesium sulfate, and the solvent was removed under reduced pressure to obtain *N*-benzyl-2-(methylthio)-*N*-phenylacetamide as a brown viscous liquid (280 mg, 72%). $^{1}$H-NMR (400 MHz, $CDCl_3$, δ): 2.21(s, 3H), 3.03 (s, 2H), 4.89 (s, 2H), 7.04 (m, 2H), 7.21 (m, 2H), 7.26 (m, 3H), 7.32 (m, 3H). $^{13}$C-NMR (400 MHz, CDCl3, δ): 16.52, 35.72, 53.43, 53.43,127.56, 128.37, 128.53, 128.70, 128.92, 129.71, 137.53, 142.34, 169.10.

## Synthesis of Covalent Organic Frameworks

### Synthesis of COF-300

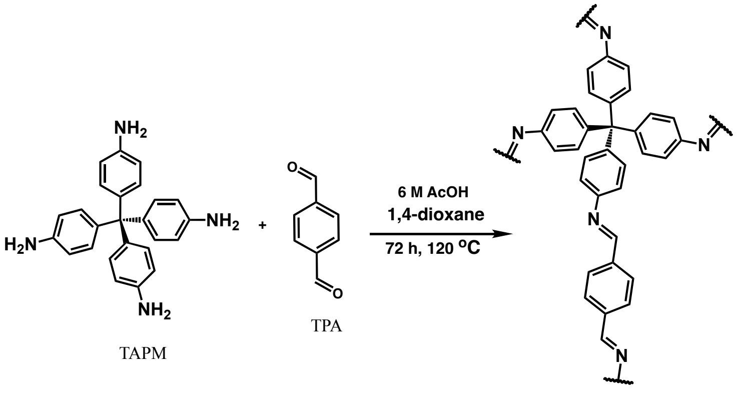


TAPM TPA COF-300

The synthesis of COF-300 was adapted from a literature procedure.[6] A 100 mL tubular solvent storage flask with a high-vacuum valve was charged with tetrakis(4-aminophenyl)methane (500 mg, 1.320 mmol), terephthaldehyde (353 mg, 2.630 mmol), 6 M aqueous acetic acid (1.3 mL, 7.880 mmol), and 1,4-dioxane (13.3 mL). The flask was flash-frozen in liquid nitrogen at 77 K and then evacuated to an internal pressure of 100 mTorr, after which it was capped to create a closed system. The reaction mixture was heated at 120 °C for 72 hours to produce a yellow solid. Upon completion, the reaction was cooled to room temperature and exposed to air. The solid was obtained through vacuum filtration and soaked in approximately 200 mL of 1,4-dioxane overnight. It was then vacuum filtered again and washed with acetone through Soxhlet extraction for 24 hours, resulting in a golden yellow COF-300 powder. Yield: 630 mg. The product was characterized using powder X-ray diffraction (Figure S13), solid-state $^{13}C$ cross-polarization magic angle spinning NMR (Figure S11) and scanning electron microscopy (Figure 4a of the main text).

**Synthesis of COF-300-AR**

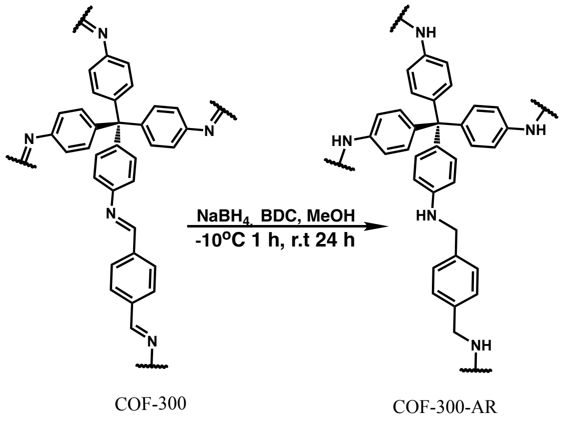


The synthesis of COF-300-AR was adapted from a literature procedure.[7] The starting material, COF-300, was activated under vacuum at 120 °C overnight. Activated COF-300 (536 mg, 3.87 mmol) was weighed in a glovebox and transferred to a Schlenk line, where it was kept under argon. COF-300 was then suspended in 278 mL of anhydrous methanol. Terephthalic acid (643 mg, 3.87 mmol) was added to the suspension and stirred at -10 °C for 5 minutes. Sodium borohydride (5.6 g, 147.06 mmol) was added in small portions over 10 minutes and stirred at -10 °C for 1 hour. The reaction mixture was warmed to room temperature and stirred for an additional 24 hours. The solids were isolated via vacuum filtration and washed three times with a large amount of water to remove unreacted sodium borohydride. The solid residue was suspended in 75 mL of ethanol overnight and vacuum filtered through nylon filter paper (0.2 μm pore size) to obtain COF-300-AR as a light-yellow powder. Yield: 487 mg. The product was characterized using powder X-ray diffraction (Figure 4l of the main text), microED (Figure 5 of the main text), solid-state $^{13}C$ cross-polarization magic-angle spinning NMR (Figure S11), $^{15}N$ cross-polarization magic-angle spinning NMR spectroscopy (Figure 2b of the main text) and scanning electron microscopy (Figure 4b of the main text).

## Synthesis of COF-300-Ac-Cl

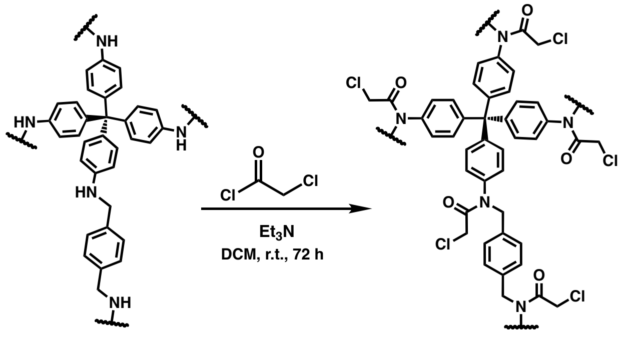


COF-300-AR COF-300-Ac-Cl

COF-300-AR was activated under vacuum at 120 °C overnight. The activated COF-300-AR (270 mg, 1.840 mmol) was weighed in a glovebox and transferred to a Schlenk line, where it was kept under argon. Anhydrous dichloromethane (12.5 mL, 0.150 M) and triethylamine (0.8 mL, 5.500 mmol) were added to the flask. The mixture was stirred and cooled to 0 °C for 15 minutes. Chloroacetyl chloride (1.3 mL, 18.36 mmol) was then added dropwise. The reaction mixture was warmed to room temperature (r.t.) and stirred for 72 hours. Afterwards, the mixture was diluted with dichloromethane (20 mL) and centrifuged to remove the solvent. The product was washed with methanol (50 mL) and water (50 mL) to remove unreacted chloroacetyl chloride. The solid product was soaked in methanol (100 mL), and the solvent was removed after 24 hours using nylon filter paper to obtain COF-300-Ac-Cl as a beige powder. Yield: 200 mg. The product was characterized using powder X-ray diffraction (Figure 4k of the main text), solid-state $^{13}C$ cross-polarization magic-angle spinning NMR (Figure 3a of the main text), scanning electron microscopy (Figure 4c of the main text), and Fourier transform infrared spectroscopy (Figure 3 of the main text).

**Synthesis of COF-300-Ac-Br**

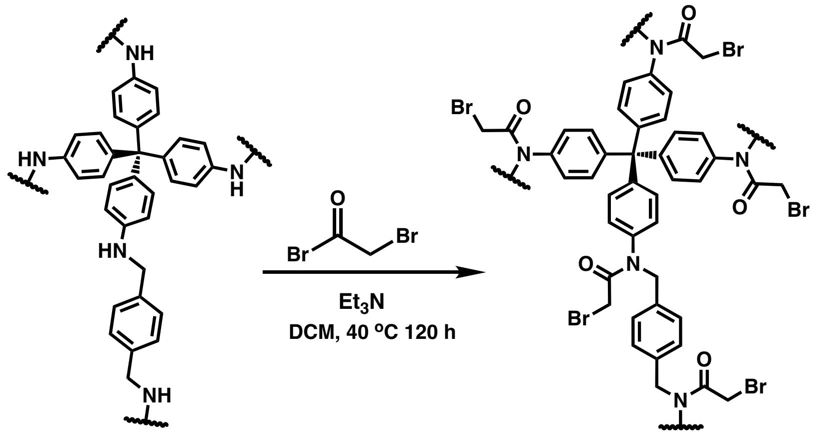


COF-300-AR

COF-300-Ac-Br

COF-300-AR was activated under vacuum at 120 °C overnight. The activated COF-300-AR (119 mg, 0.820 mmol) was weighed inside a glovebox and transferred to a Schlenk line, where it was kept under argon. Anhydrous dichloromethane (50 mL, 0.016 M) and triethylamine (0.9 mL, 6.600 mmol) were added to the flask. The mixture was stirred and cooled to 0 °C for 15 minutes. Bromoacetyl bromide (2.1 mL, 24.60 mmol) was then added dropwise. The reaction mixture was warmed to room temperature and stirred for 5 hours. It was then refluxed at 40 °C for 115 hours. Afterwards, the mixture was diluted with dichloromethane (50 mL) and centrifuged to remove the solvent. The product was washed with methanol (50 mL) and water (50 mL) to eliminate unreacted chloroacetyl chloride. The solid product was soaked in methanol (100 mL), and the solvent was removed after 24 hours using nylon filter paper to obtain COF-300-Ac-Br as a beige powder. Yield: 98 mg. The product was characterized by powder X-ray diffraction (Figure 4k of the main text), solid-state $^{13}C$ cross-polarization magic angle spinning NMR (Figure 2a of the main text), $^{15}N$ cross-polarization magic-angle spinning NMR spectroscopy (Figure 2b of the main text), scanning electron microscopy (Figure 4d of the main text), and Fourier transform infrared spectroscopy (Figure 3 of the main text).

## Synthesis of COF-300-$N_3$

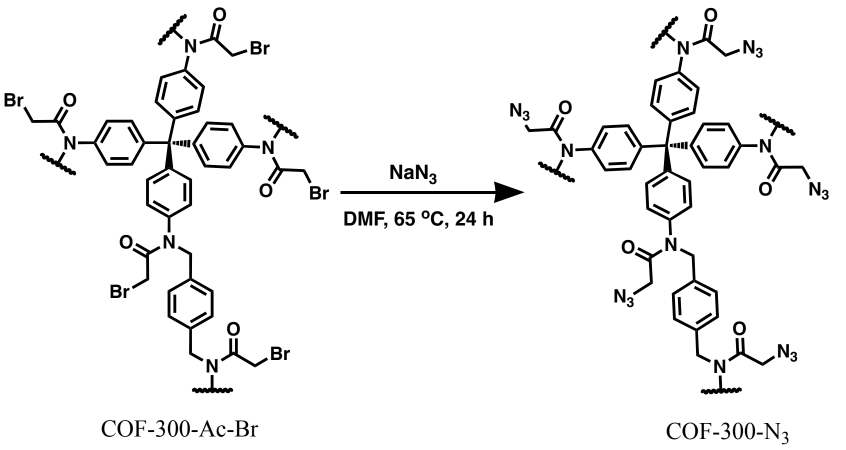

COF-300-Ac-Br was activated under vacuum at 90 °C overnight. Activated COF-300-Ac-Br (167 mg, 0.530 mmol) was weighed in a glovebox and transferred to a Schlenk line, where it was kept under argon. The sample was suspended in anhydrous DMF (50 mL, 0.011 M). Sodium azide* (706 mg, 10.62 mmol) was then added to the reaction mixture. The reaction was refluxed at 65 °C for 24 hours. Afterwards, the resulting mixture was diluted with 50 mL of DMF and centrifuged to remove the solvent. The product was washed with methanol (50 mL) and water (50 mL) twice. The solid product was soaked in methanol (100 mL), and the solvent was removed after 24 hours using nylon filter paper to obtain COF-300-$N_3$ as a beige powder. Yield: 84 mg. The product was characterized by powder X-ray diffraction (Figure 4k of the main text), solid-state $^{13}C$ cross-polarization magic angle spinning NMR (Figure 2c of the main text), scanning electron microscopy (Figure 4f of the main text), and Fourier transform infrared spectroscopy (Figure 3 of the main text).

*We note that sodium azide ($NaN_3$) is acutely toxic and requires rigorous adherence to safety procedures and use of personal protective equipment (PPE) to be handled safely.

**Synthesis of COF-300-CN**

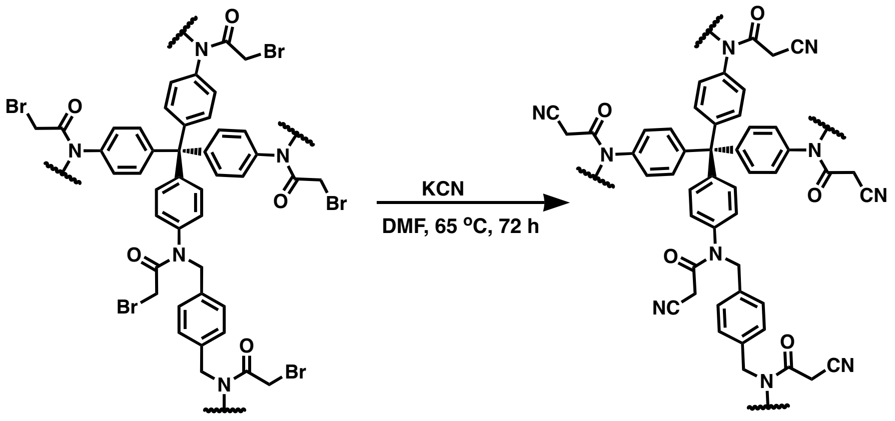

COF-300-Ac-Br COF-300-CN

COF-300-Ac-Br was activated under vacuum at 90 °C overnight. Activated COF-300-Ac-Br (120 mg, 0.520 mmol) was weighed in a glovebox and transferred to a Schlenk line, where it was kept under argon. The sample was suspended in anhydrous DMF (50 mL, 0.010 M). Potassium cyanide* (689 mg, 10.60 mmol) was then added to the reaction mixture. It was refluxed at 65 °C for 72 hours. Afterwards, the resulting mixture was diluted with 50 mL of DMF and centrifuged to remove the solvent. The product was washed twice with methanol (50 mL) and water (50 mL). The solid product was soaked in methanol, and the solvent was removed after 24 hours using nylon filter paper to obtain COF-300-CN. Yield: 83 mg. The product was characterized using powder X-ray diffraction (Figure 4k of the main text), $^{13}C$ cross-polarization magic angle spinning solid-state NMR ((Figure 2c of the main text), and scanning electron microscopy (Figure 4e of the main text).
*We note that potassium cyanide (KCN) is acutely toxic and requires rigorous adherence to safety procedures and use of personal protective equipment (PPE) to be handled safely.

**Synthesis of COF-300-$NH_2$**

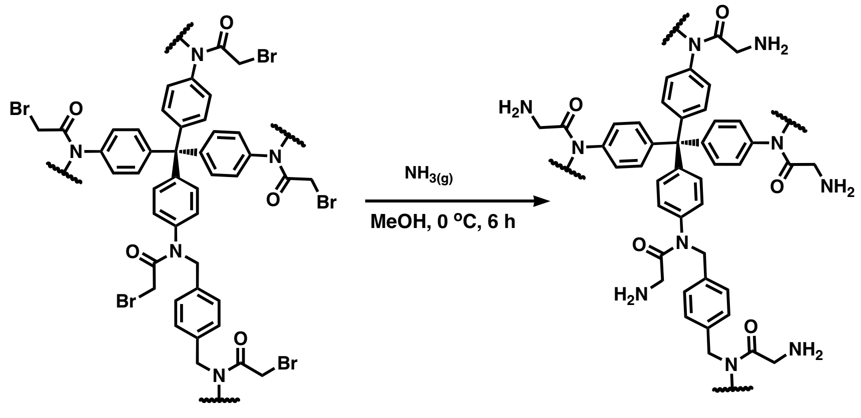


COF-300-Ac-Br COF-300-$NH_2$

COF-300-Ac-Br (200 mg, 0.69 mmol), activated under vacuum at 90 °C overnight, was weighed inside a glovebox and transferred to the reaction setup (Figure S44). The sample was suspended in methanol (50 mL). The reaction mixture was cooled to 0 °C. Excess ammonia gas was bubbled through the mixture at 0 °C for 6 hours. The ammonia was generated in situ by reacting ammonium hydroxide (28-30%, 300 mL) released dropwise onto sodium hydroxide pellets (20 g) in another flask. Afterward, the mixture was diluted with water (20 mL) and stirred for one hour. It was then centrifuged, and the supernatant was decanted. The obtained solid product was soaked in 100 mL of methanol, and the solvent was removed after 24 hours using nylon filter paper to yield COF-300-$NH_2$. Yield: 185 mg. The product was characterized with powder X-ray diffraction (Figure 4k of the main text), microED (Figure 5 of the main text $^{13}C$ cross-polarization magic-angle spinning solid-state NMR (Figure 2c of the main text), $^{15}N$ cross-polarization magic-angle spinning NMR spectroscopy (Figure 2b of the main text), and scanning electron microscopy (Figure 4g of the main text).

**Synthesis of COF-300-OH**

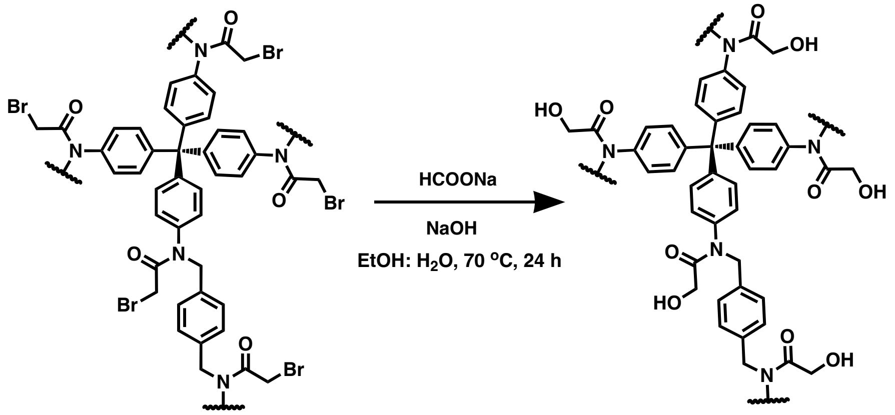


COF-300-Ac-Br COF-300-OH

COF-300-Ac-Br (200 mg, 0.880 mmol), activated under vacuum at 90 °C overnight, was weighed in a glovebox and transferred to a Schlenk line, where it was kept under argon. The sample was suspended in 20 mL of a 2:1 methanol/water mixture (v/v). Next, sodium formate (1.2 g, 17.60 mmol) and a catalytic amount of sodium hydroxide (18 mg, 0.440 mmol) were added to the reaction mixture. The mixture was then refluxed at 70 °C for 24 hours. Afterwards, the mixture was diluted with methanol (30 mL) and centrifuged. The supernatant was decanted, and the obtained solid product was washed twice with methanol (50 mL) and water (50 mL). The solid product was soaked in methanol, and the solvent was removed after 24 hours using nylon filter paper to obtain COF-300-OH. Yield: 173 mg. The product was characterized using powder X-ray diffraction (Figure 4k of the main text), $^{13}C$ cross-polarization magic angle spinning solid-state NMR (Figure 2d of the main text), scanning electron microscopy (Figure 4h of the main text), and Fourier transform infrared spectroscopy (Figure 3 of the main text).

## Synthesis of COF-300-OMe

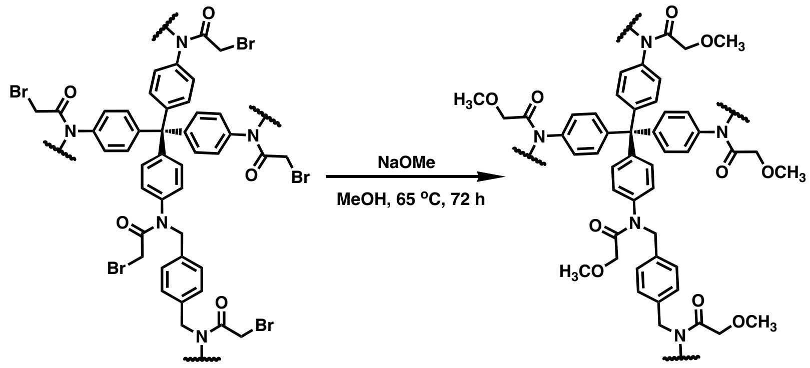


COF-300-Ac-Br COF-300-OMe

COF-300-Ac-Br (188 mg, 0.850 mmol), activated under vacuum at 90 °C overnight, was weighed in a glovebox and transferred to a Schlenk line, where it was kept under argon. The sample was suspended in methanol (50 mL, 0.017 M). Then, sodium methoxide (920 mg, 17.00 mmol) was added to the reaction mixture. The mixture was refluxed at 65 °C for 72 hours. Afterwards, the mixture was diluted with methanol (50 mL) and centrifuged to remove the solvent. The product was washed twice with methanol (50 mL) and water (50 mL) each. The solid product was soaked in 100 mL of methanol, and the solvent was removed after 24 hours using nylon filter paper to obtain COF-300-OMe. Yield: 189 mg. The product was characterized using powder X-ray diffraction (Figure 4k of the main text), $^{13}C$ cross-polarization magic angle spinning solid-state NMR (Figure 2d of the main text), and scanning electron microscopy (Figure 4i of the main text).

**Synthesis of COF-300-SMe**

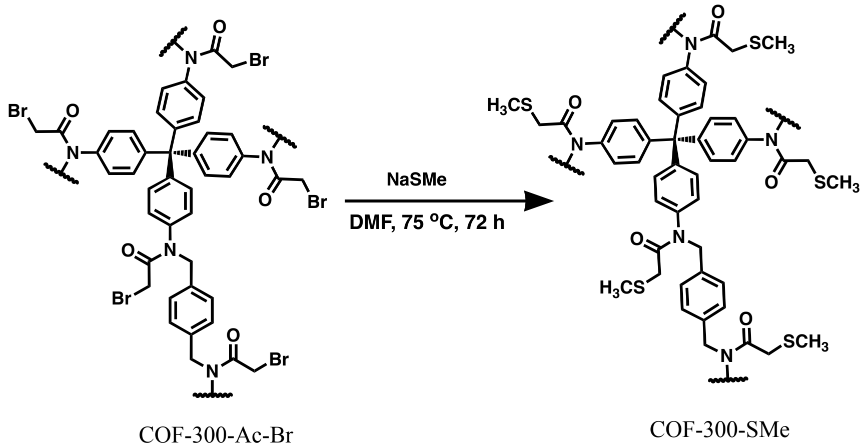


COF-300-Ac-Br (115 mg, 0.520 mmol) was activated under vacuum at 90 °C overnight and then weighed in a glovebox and transferred to a Schlenk line, where it was kept under argon. The sample was suspended in DMF (50 mL, 0.011 M). Then, sodium methanethiolate (734 mg, 10.47 mmol) was added to the reaction mixture. It was refluxed at 75 °C for 72 hours. Afterwards, the mixture was diluted with 50 mL DMF and centrifuged to remove the solvent. The product was washed with methanol (50 mL) and water (50 mL) twice. The solid product was soaked in 100 mL methanol, and the solvent was removed after 24 hours using nylon filter paper to obtain COF-300-SMe. Yield: 120 mg. The product was characterized using powder X-ray diffraction (Figure 4k of the main text), $^{13}C$ cross-polarization magic angle spinning solid-state NMR (Figure 2d of the main text), and scanning electron microscopy (Figure 4j of the main text).

## Supplementary Figures

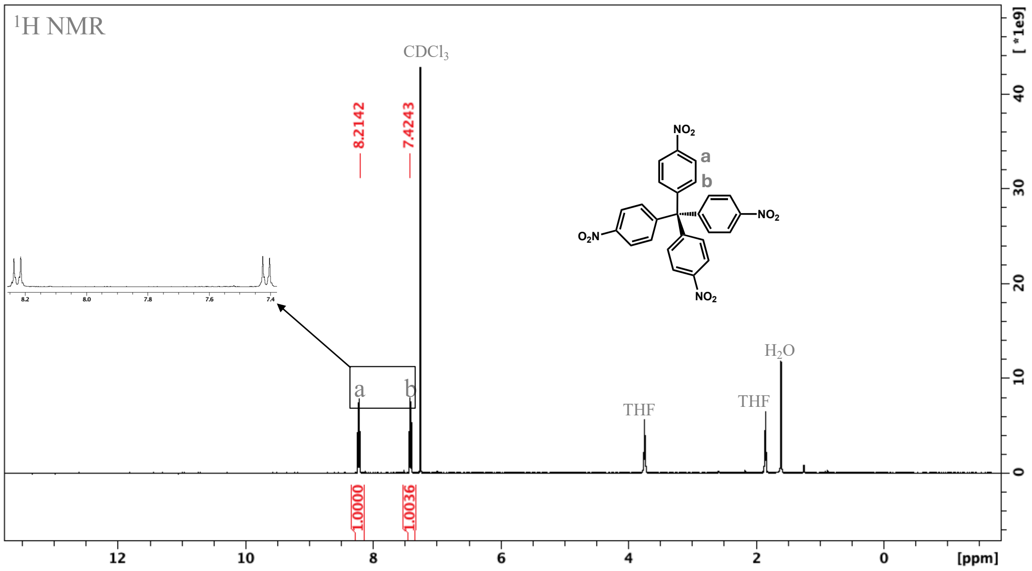


**Figure S1.** ¹H NMR spectrum (400 MHz, $CDCl_3$) of tetrakis(4-nitrophenyl)methane.

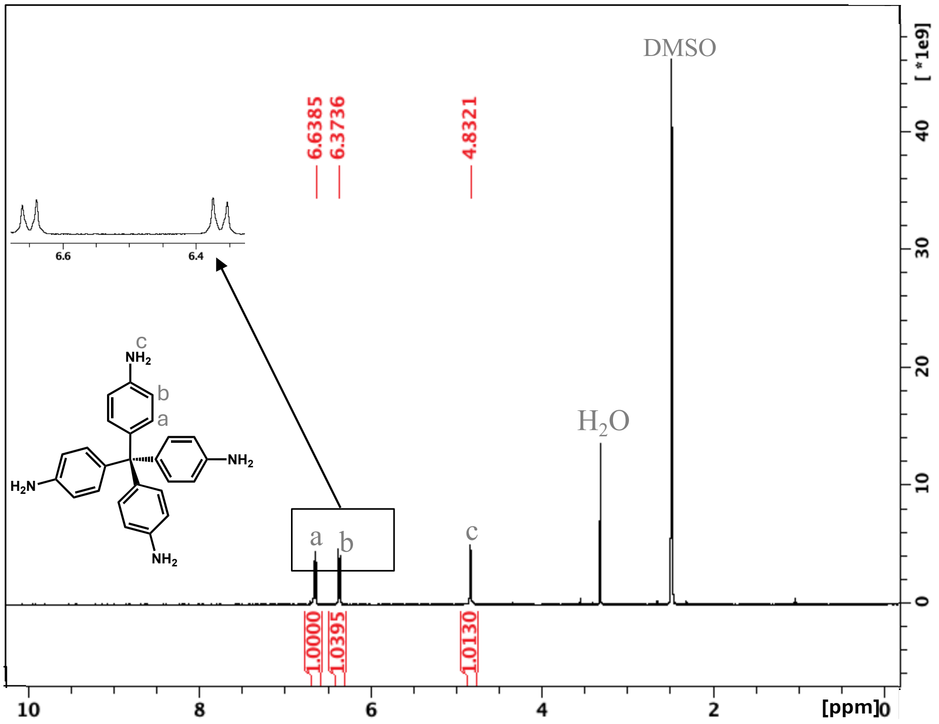


**Figure S2.** ¹H NMR spectrum (400 MHz, DMSO-$d_6$) of tetrakis(4-aminophenyl)methane.

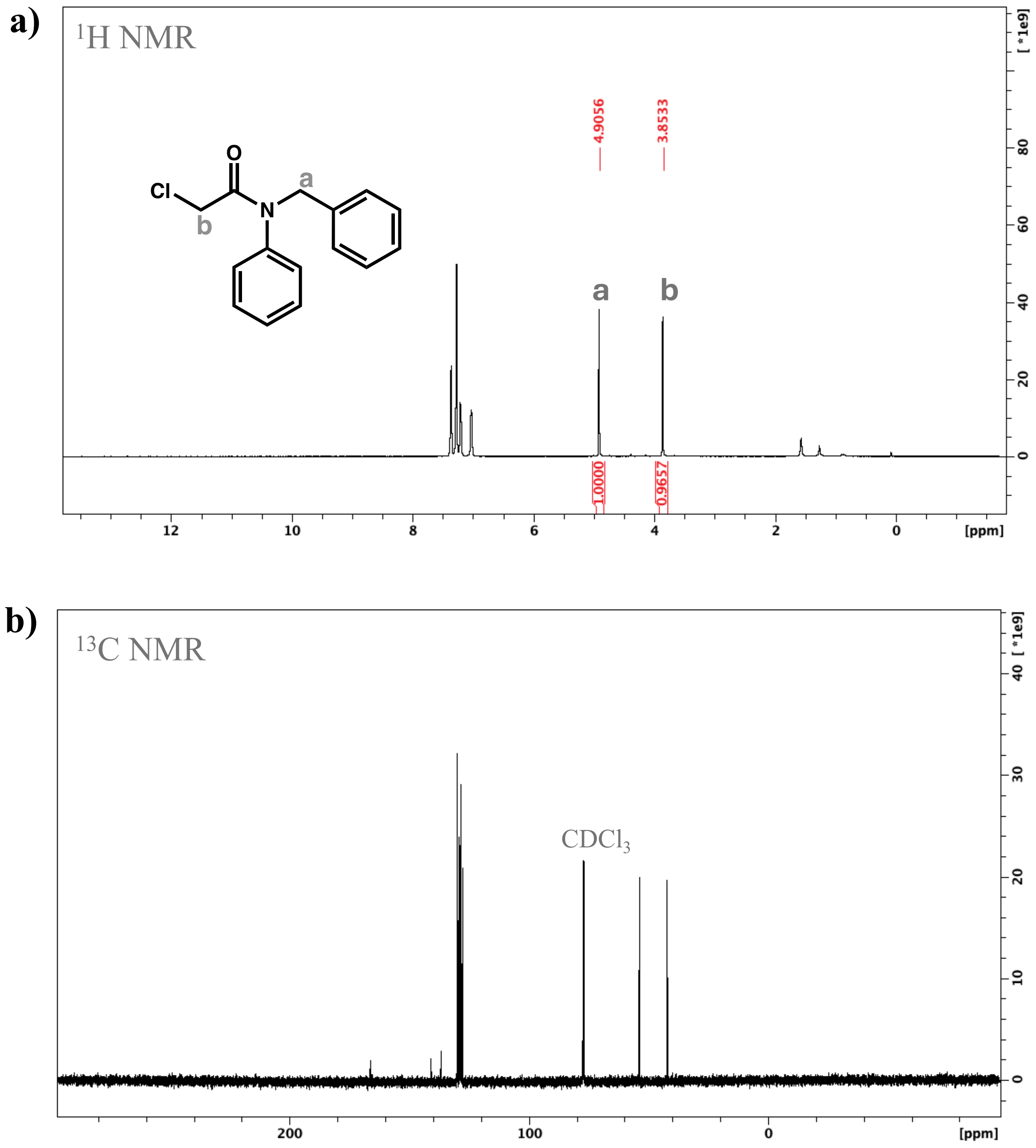


**Figure S3.** (a) $^{1}H$ NMR spectrum (400 MHz, $CDCl_3$) of the model compound of COF-300-Ac-Cl (*N*-benzyl-2-chloro-*N*-phenylacetamide). (b) $^{13}C$ NMR spectrum (400 MHz, $CDCl_3$) of *N*-benzyl-2-chloro-*N*-phenylacetamide.

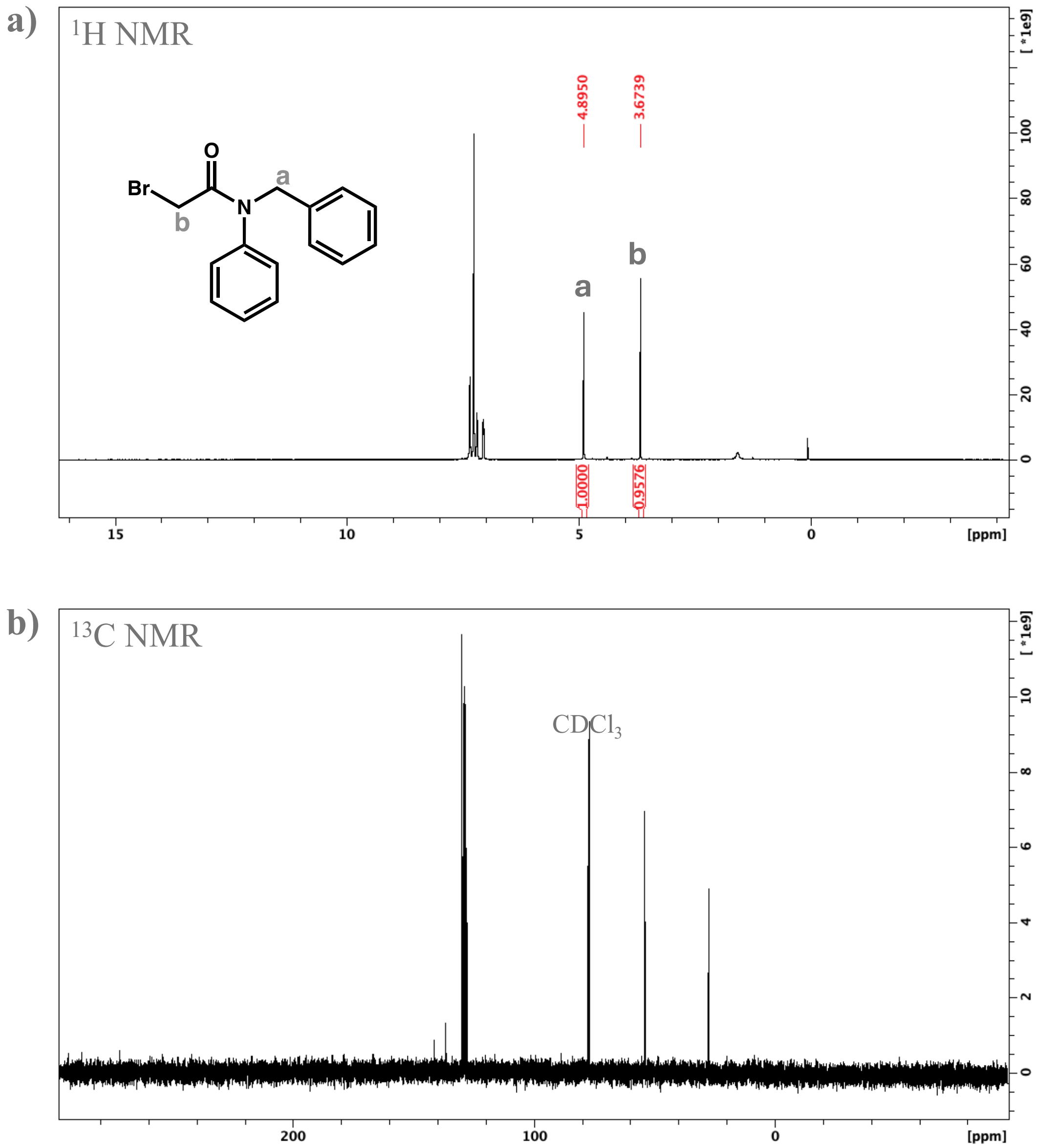


**Figure S4.** (a) $^1$H NMR spectrum (400 MHz, $CDCl_3$) of the model compound of COF-300-Ac-Br (*N*-benzyl-2-bromo-*N*-phenylacetamide). (b) $^{13}$C NMR spectrum (400 MHz, $CDCl_3$) of *N*-benzyl-2-bromo-*N*-phenylacetamide.

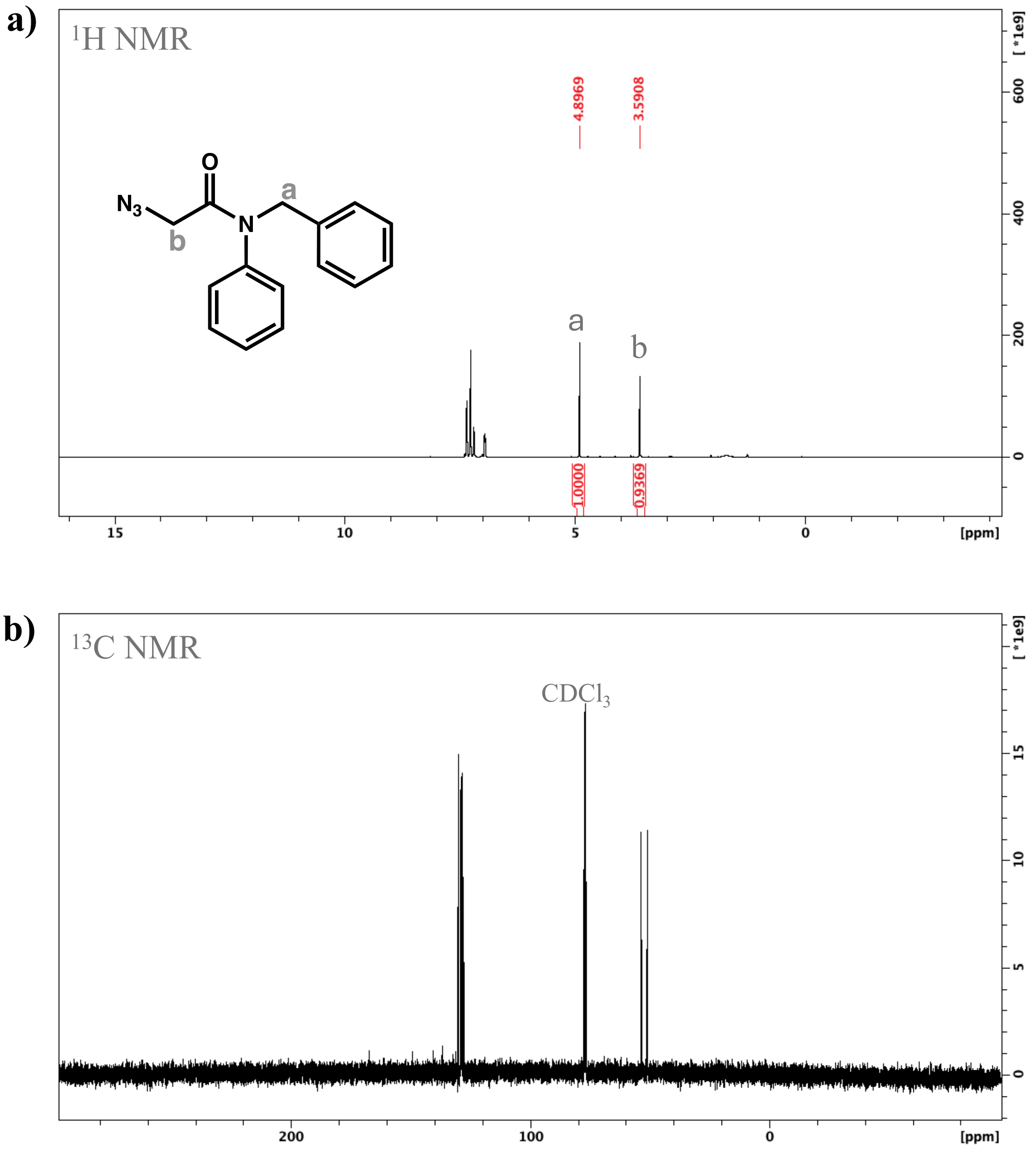


**Figure S5.** (a) $^{1}$H NMR spectrum (400 MHz, $CDCl_3$) of the model compound of COF-300-$N_3$ (2-azido-*N*-benzyl-*N*-phenylacetamide). (b) $^{13}$C NMR spectrum (400 MHz, $CDCl_3$) of 2-azido-*N*-benzyl-*N*-phenylacetamide.

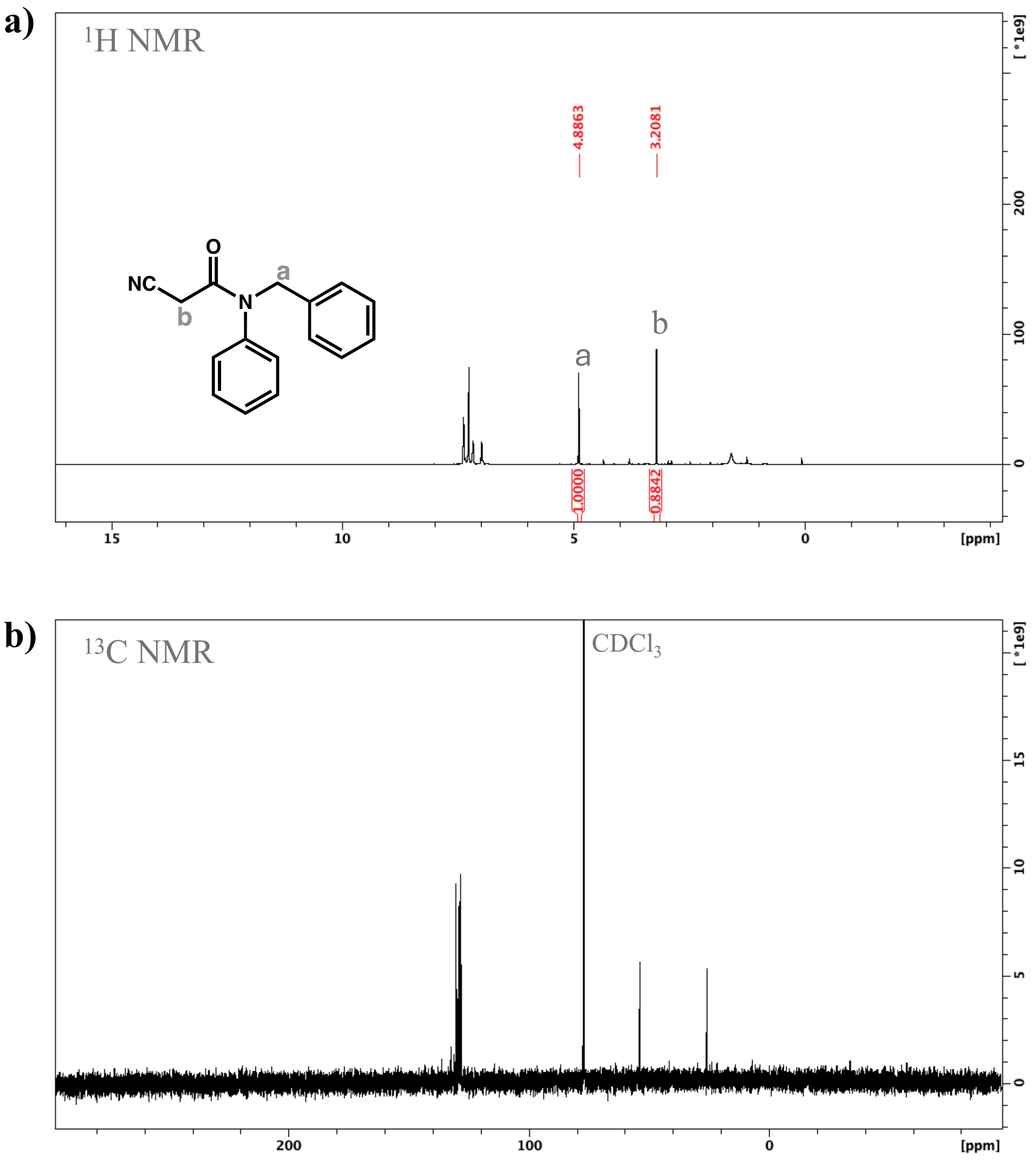


**Figure S6.** (a) $^{1}H$ NMR spectrum (400 MHz, $CDCl_3$) of the model compound of COF-300-CN (*N*-benzyl-2-cyano-*N*-phenylacetamide). (b) $^{13}C$ NMR spectrum (400 MHz, $CDCl_3$) of *N*-benzyl-2-cyano-*N*-phenylacetamide.

a)
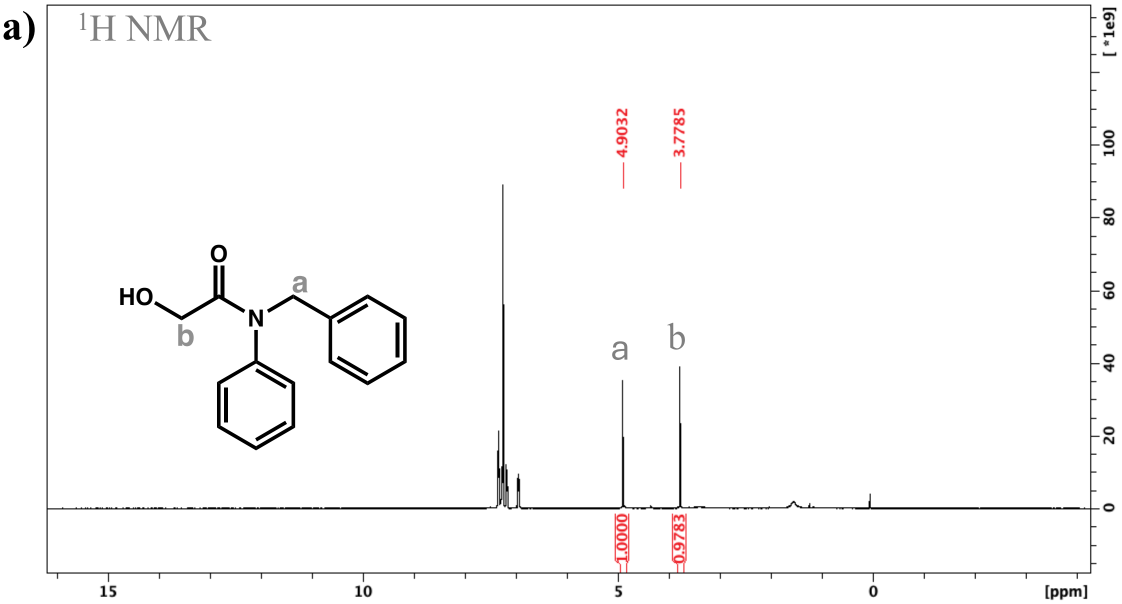


b)
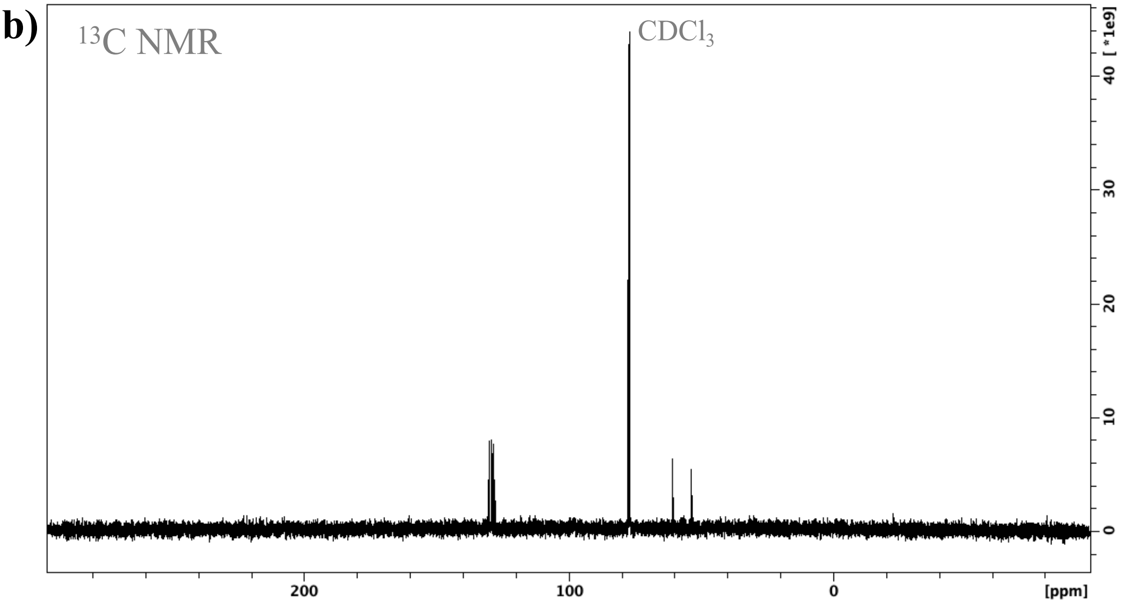


**Figure S7.** (a) $^1H$ NMR spectrum (400 MHz, $CDCl_3$) of the model compound of COF-300-OH (*N*-benzyl-2-hydroxy-*N*-phenylacetamide), (b) $^{13}C$ NMR spectrum (400 MHz, $CDCl_3$) of *N*-benzyl-2-hydroxy-*N*-phenylacetamide.

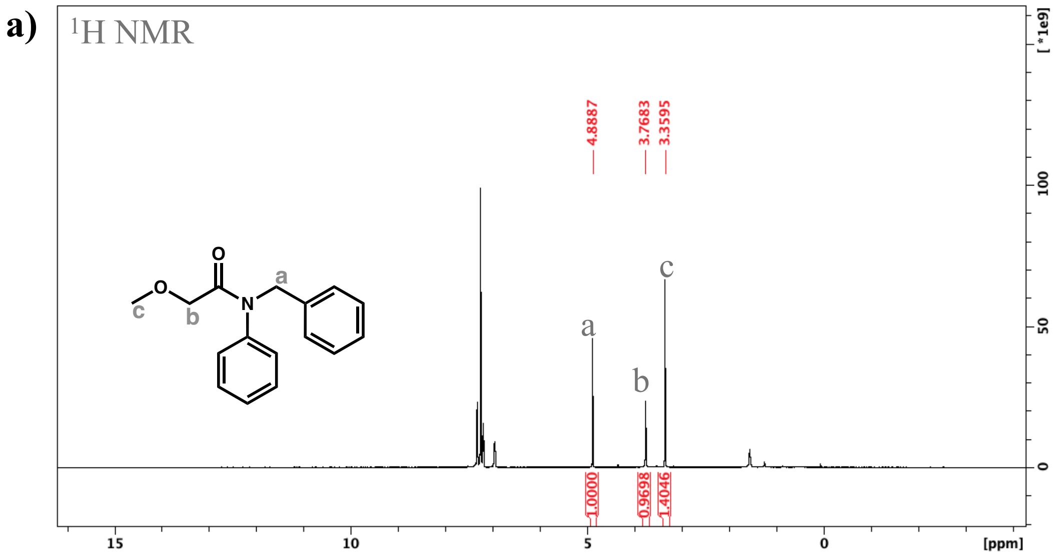


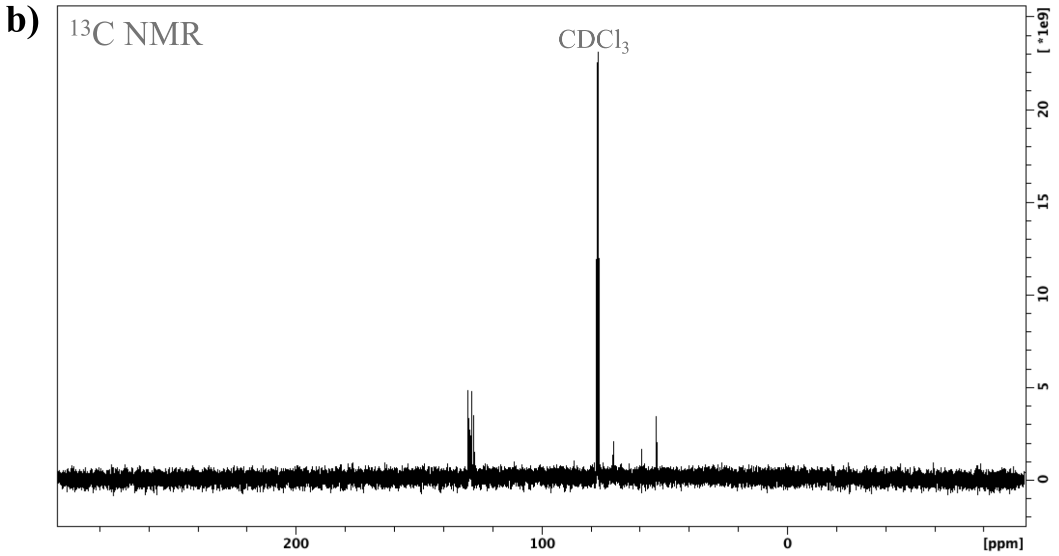


**Figure S8.** (a) $^{1}H$ NMR spectrum (400 MHz, $CDCl_3$) of the model compound of COF-300-OMe (*N*-benzyl-2-methoxy-*N*-phenylacetamide). (b) $^{13}C$ NMR spectrum (400 MHz, $CDCl_3$) of *N*-benzyl-2-methoxy-*N*-phenylacetamide.

a)

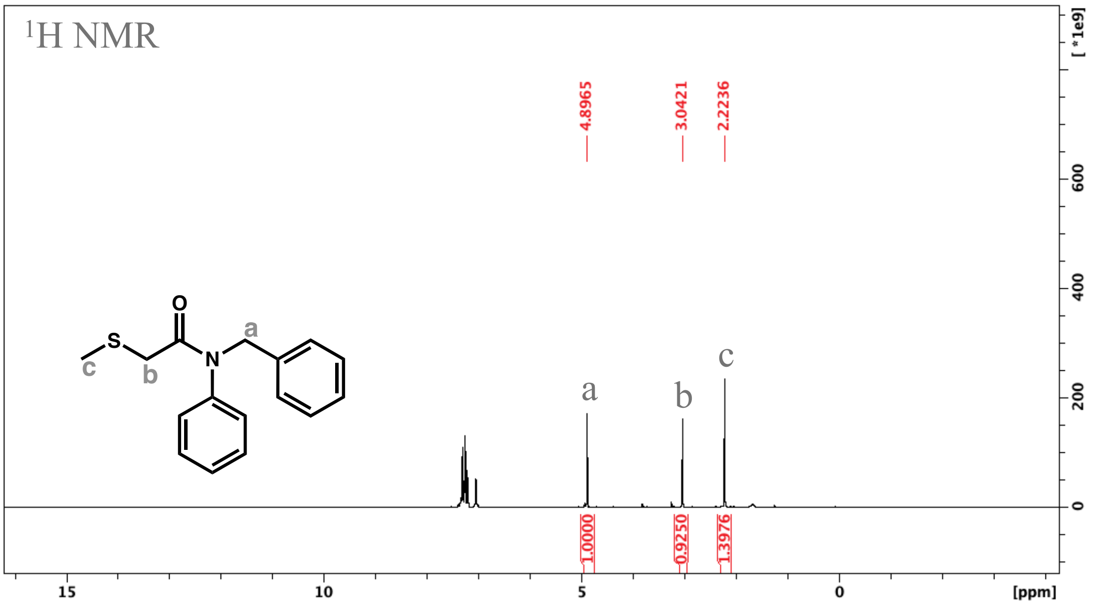


b)

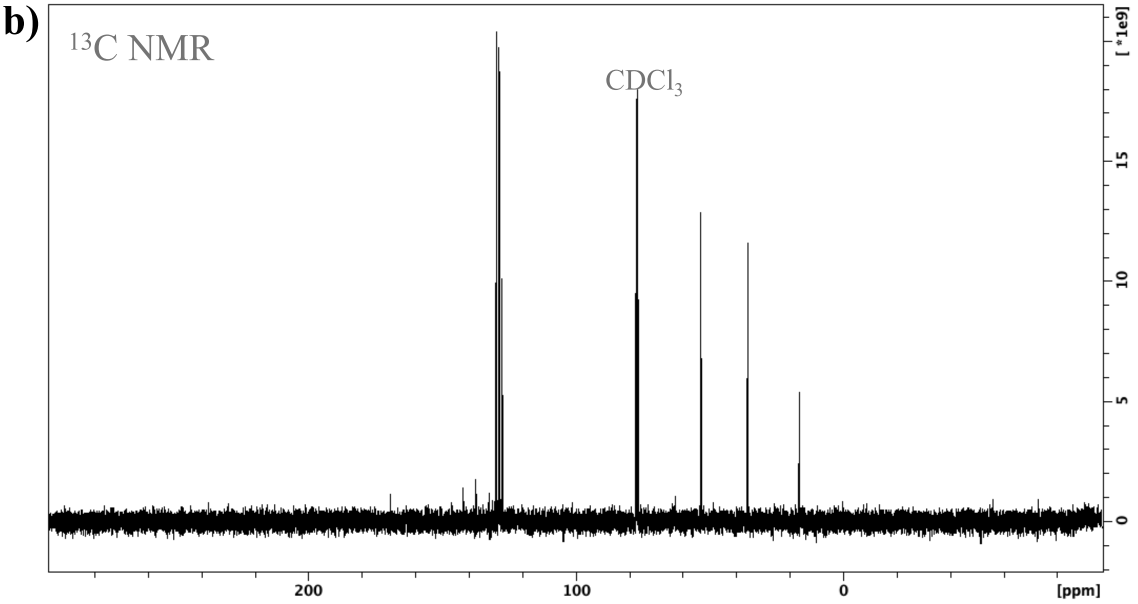


**Figure S9.** (a) $^{1}$H NMR spectrum (400 MHz, $CDCl_3$) of model compound of COF-300-SMe (*N*-benzyl-2-(methylthio)-*N*- phenylacetamide). (b) $^{13}$C NMR spectrum (400 MHz, $CDCl_3$) of *N*-benzyl-2-(methylthio)-*N*-phenylacetamide.

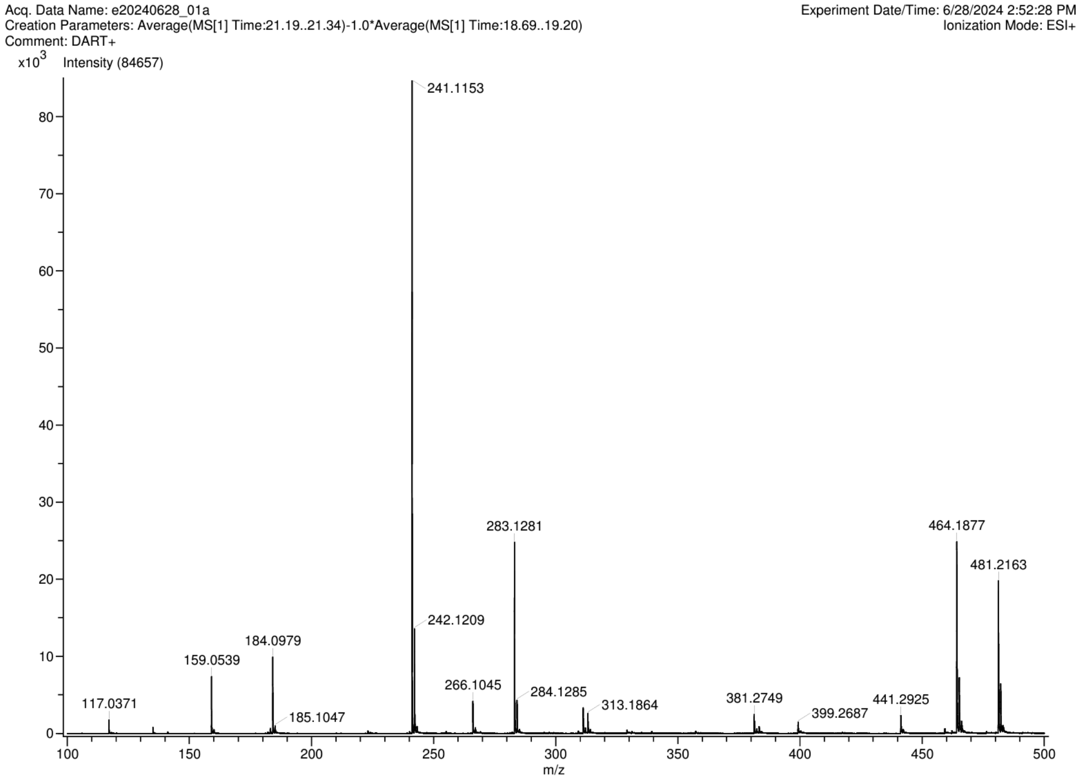


**Figure S10.** MS (DART) spectrum of the model compound of COF-300-$NH_2$ (2-amino-*N*-benzyl-*N*-phenylacetamide), $[M+H]^+$: 241.1153.

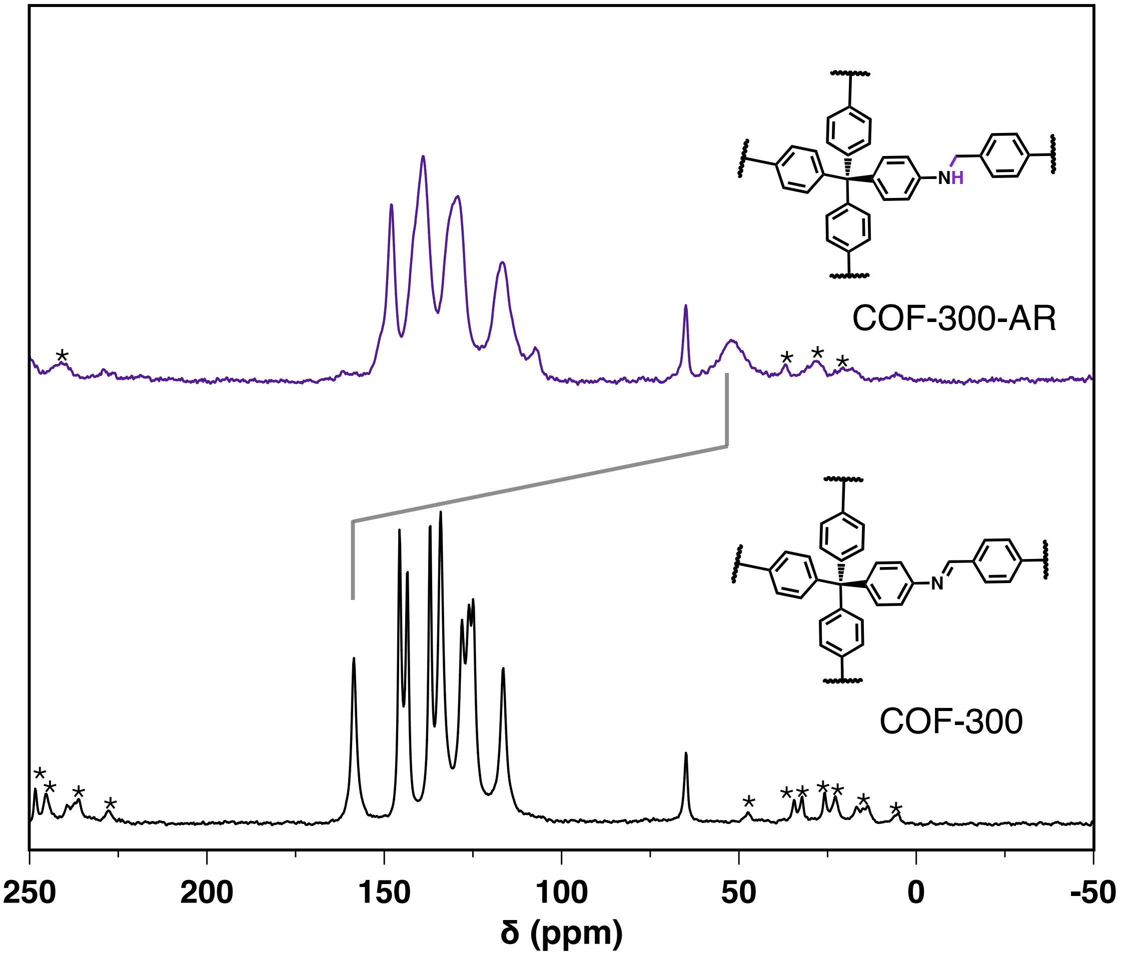


**Figure S11.** $^{13}C$ CP/MAS ssNMR spectra of COF-300 (black) and COF-300-AR (purple) showing the disappearance of imine carbon and the appearance of an amine carbon.

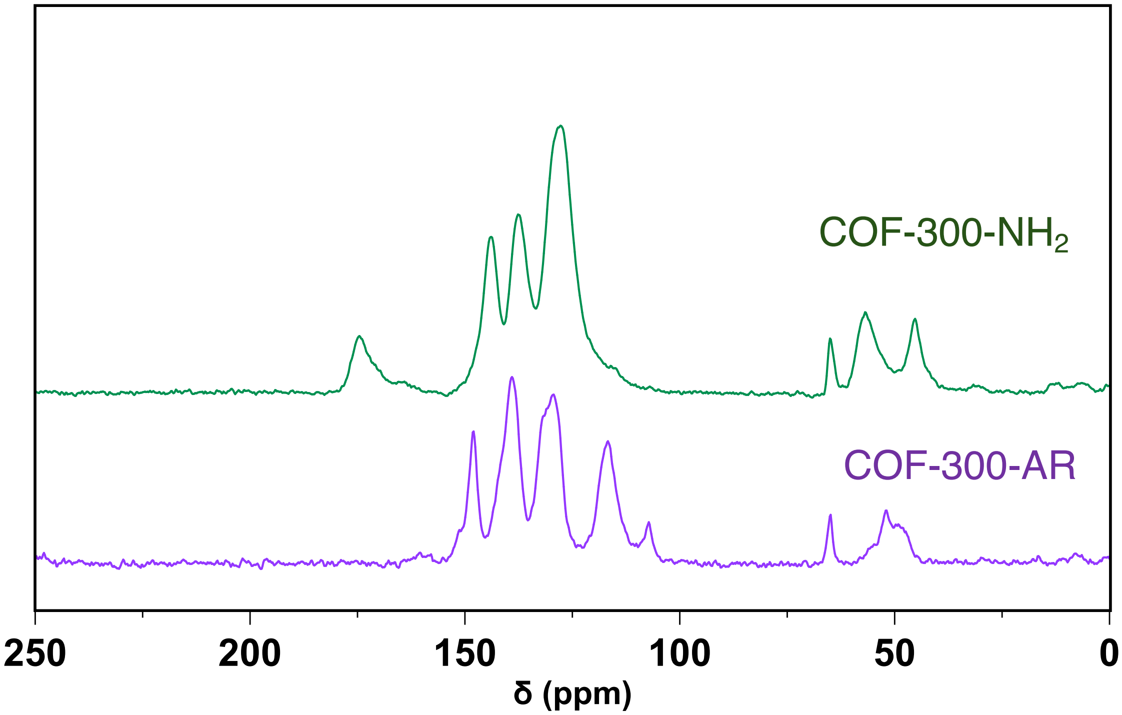


**Figure S12.** ssNMR spectra of COF-300-$NH_2$ and COF-300-AR after $CO_2$ desorption. Spectra remain unchanged after $CO_2$ adsorption-desorption experiment confirming physisorption $CO_2$ framework interaction.

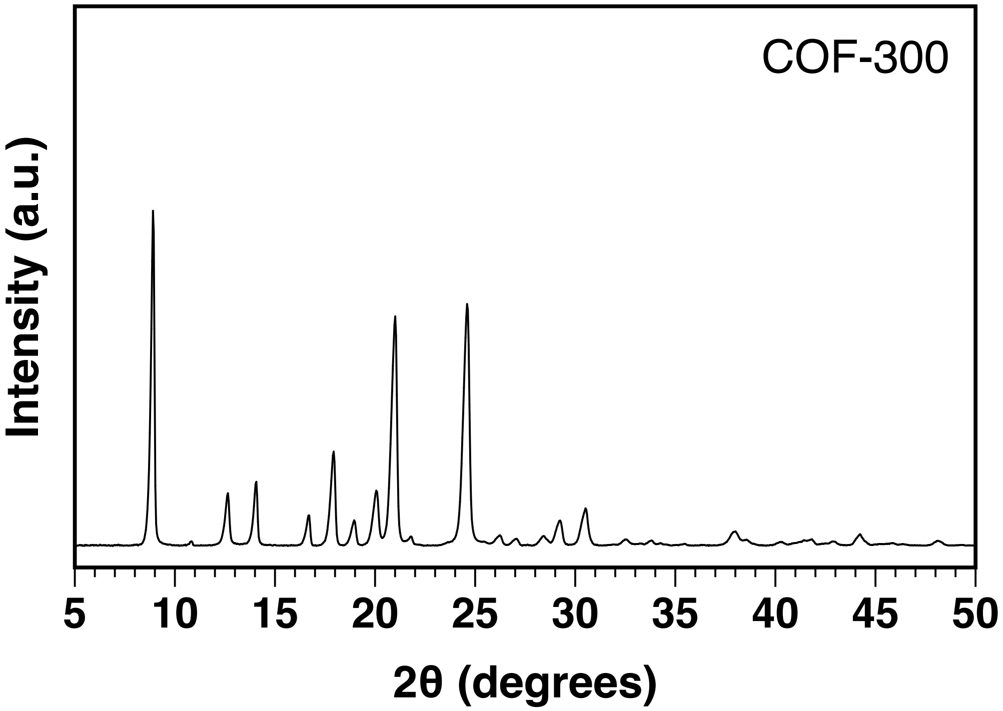


**Figure S13.** PXRD pattern of COF-300 at ambient conditions in air.

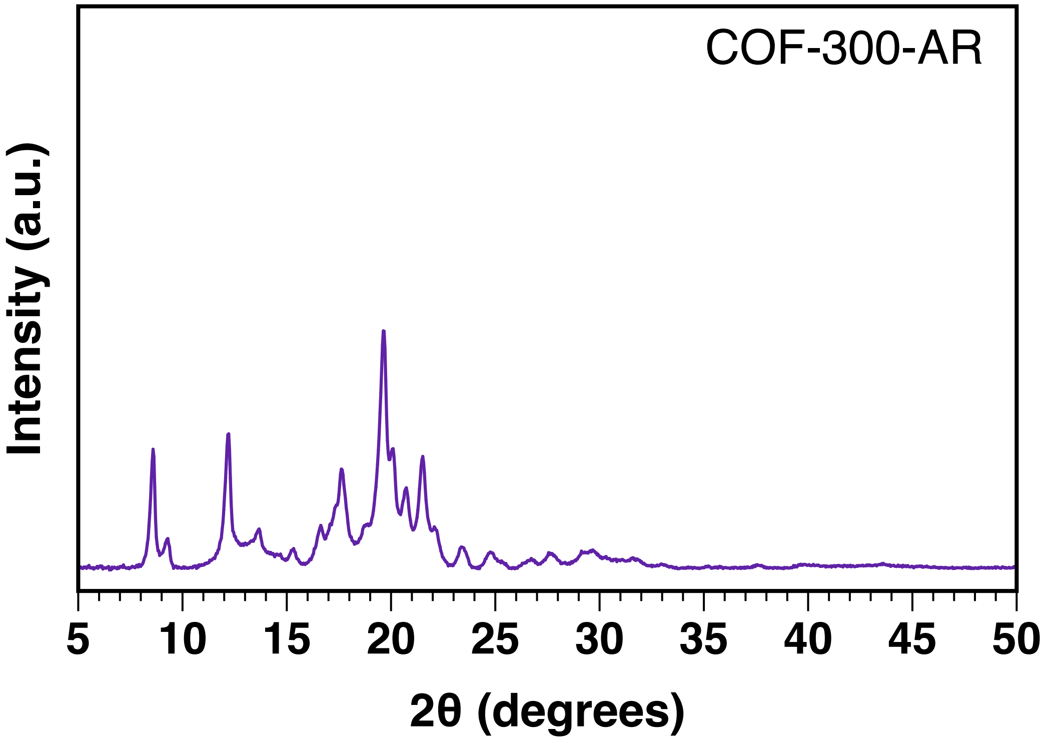


**Figure S14.** PXRD pattern of COF-300-AR at ambient conditions in air.

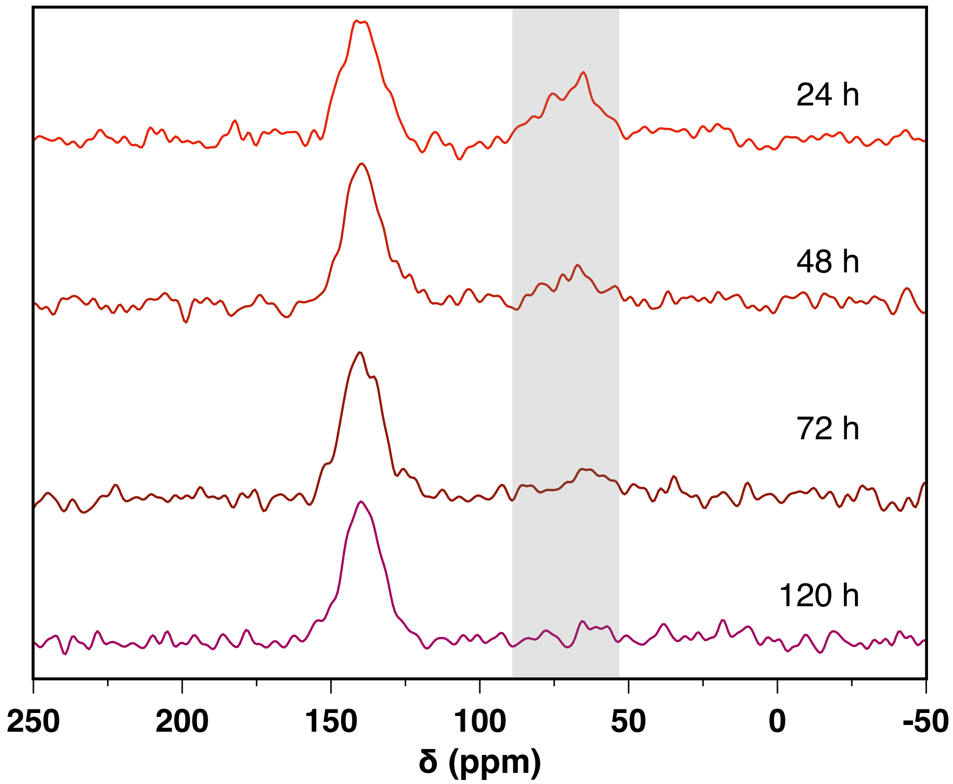


**Figure S15.** $^{15}N$ CP/MAS ssNMR spectra of COF-300-Ac-Br showing conversion at different reaction times.

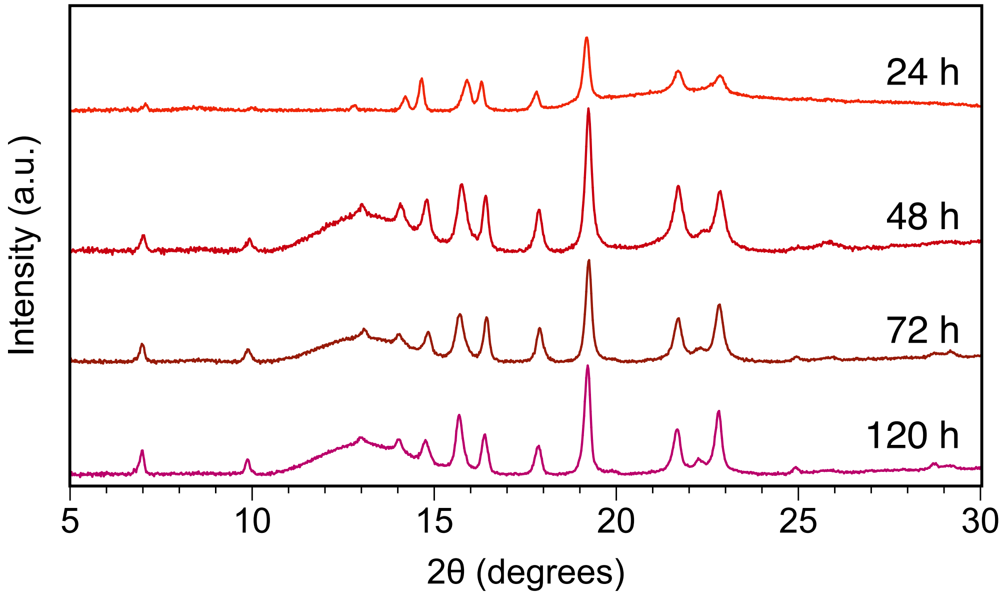


**Figure S16.** PXRD of COF-300-Ac-Br at different reaction times.

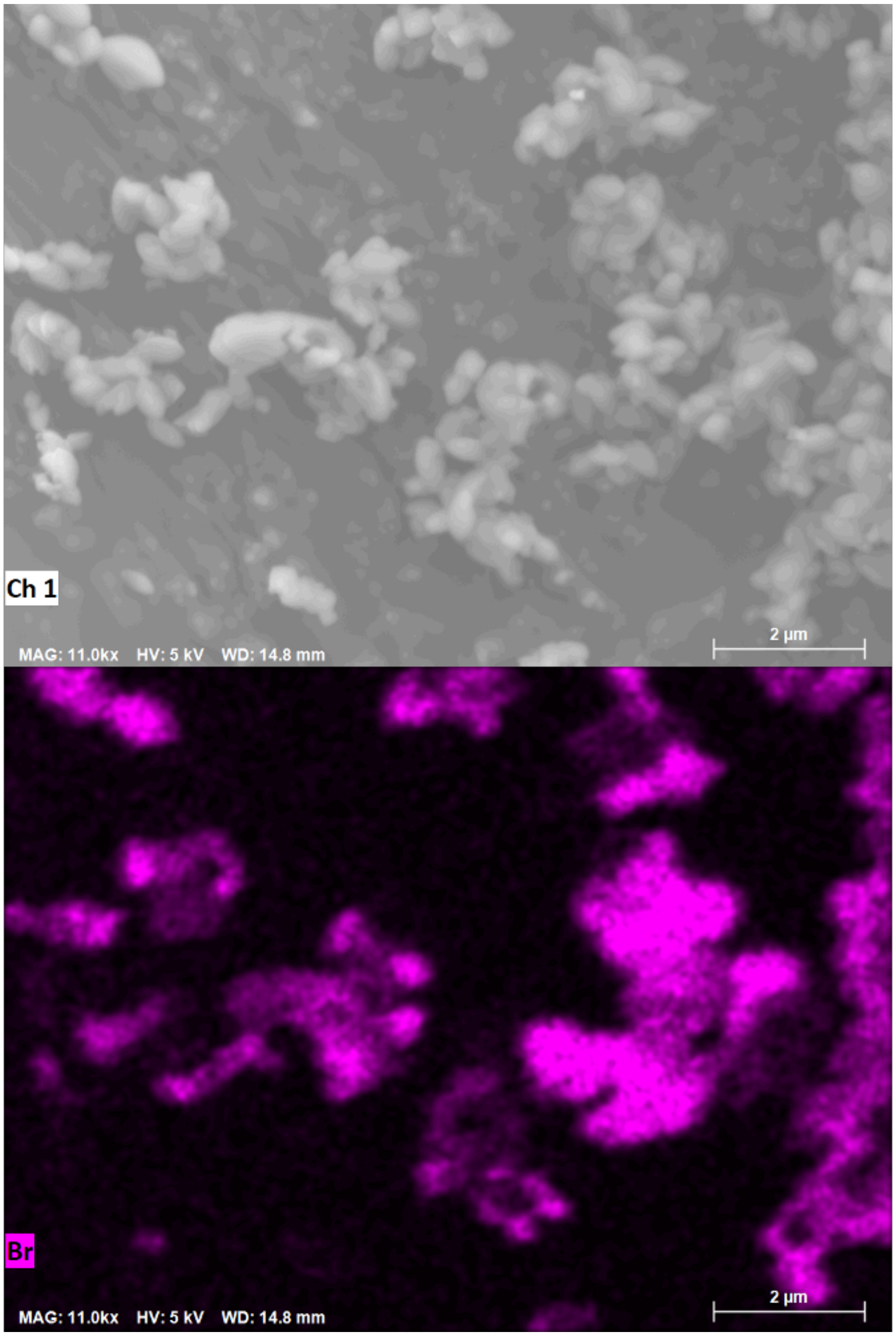


**Figure S17.** EDX analysis of COF-300-Ac-Br showing homogenous distribution of Br within the COF.

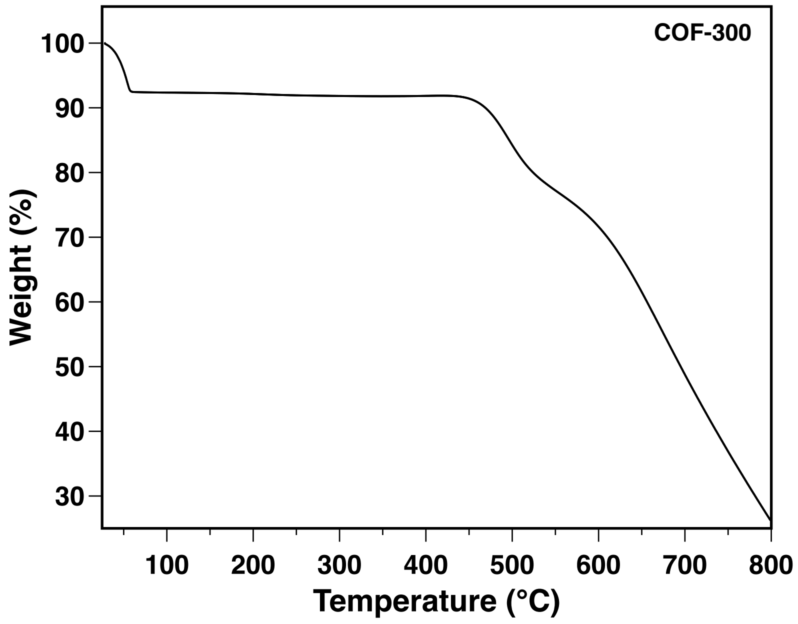


**Figure S18.** Thermogravimetric analysis (TGA) of COF-300.

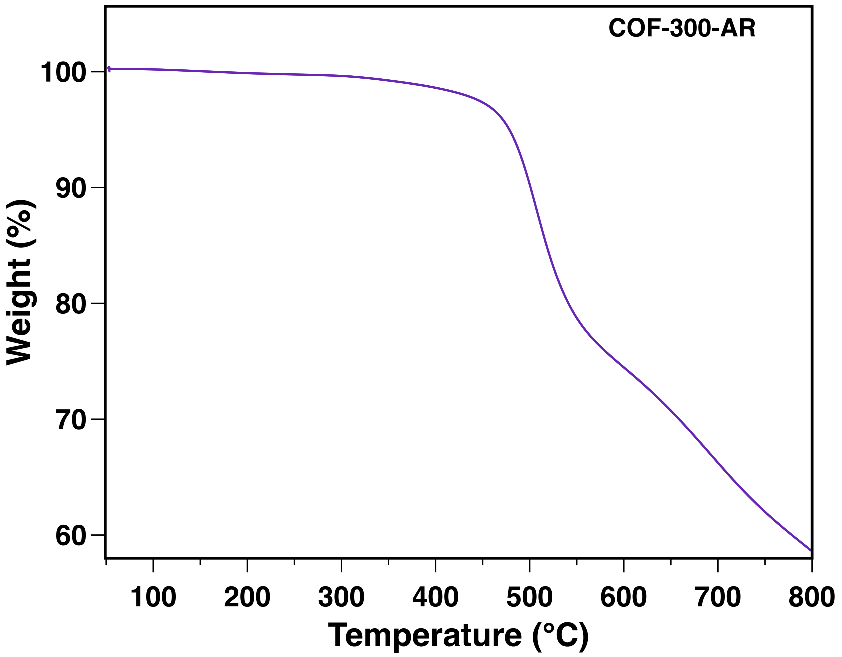


**Figure S19.** TGA of COF-300-AR.

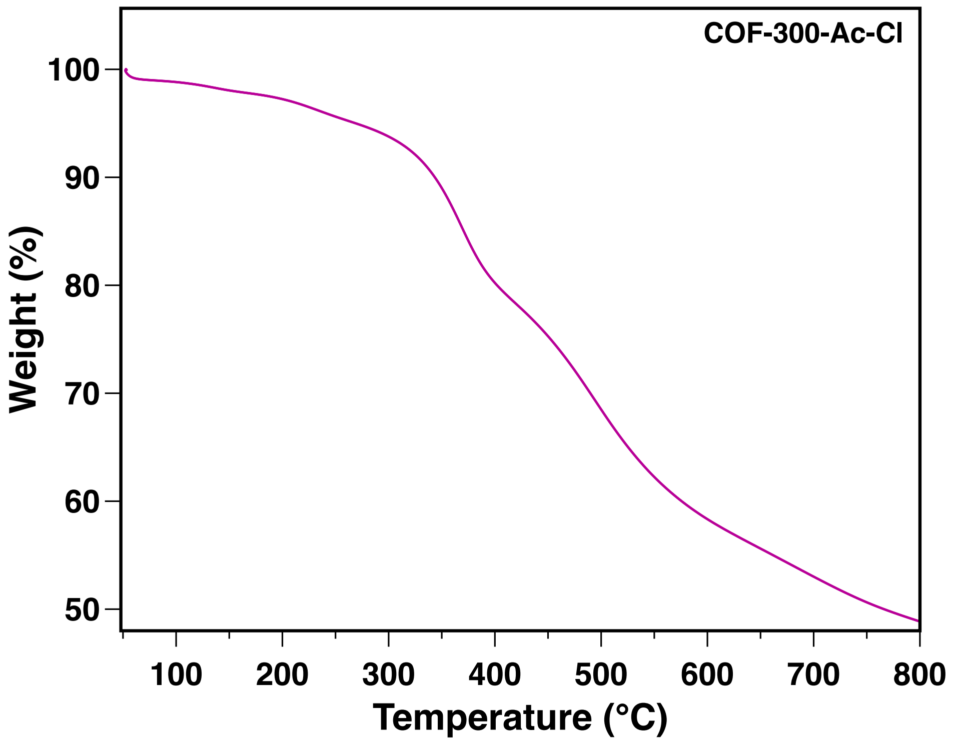


**Figure S20.** TGA of COF-300-Ac-Cl.

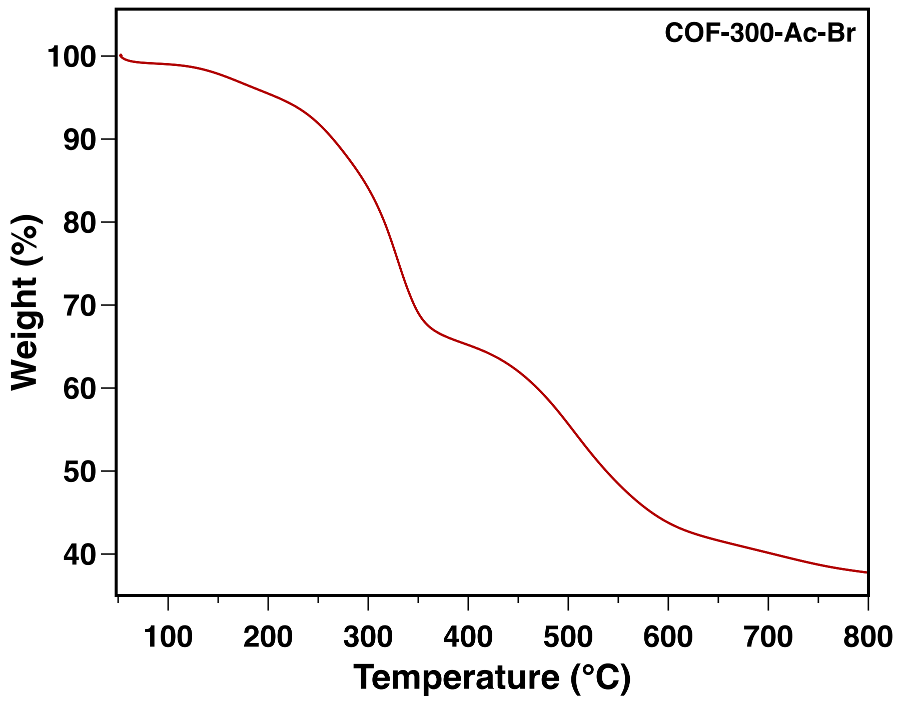


**Figure S21:** TGA of COF-300-Ac-Br.

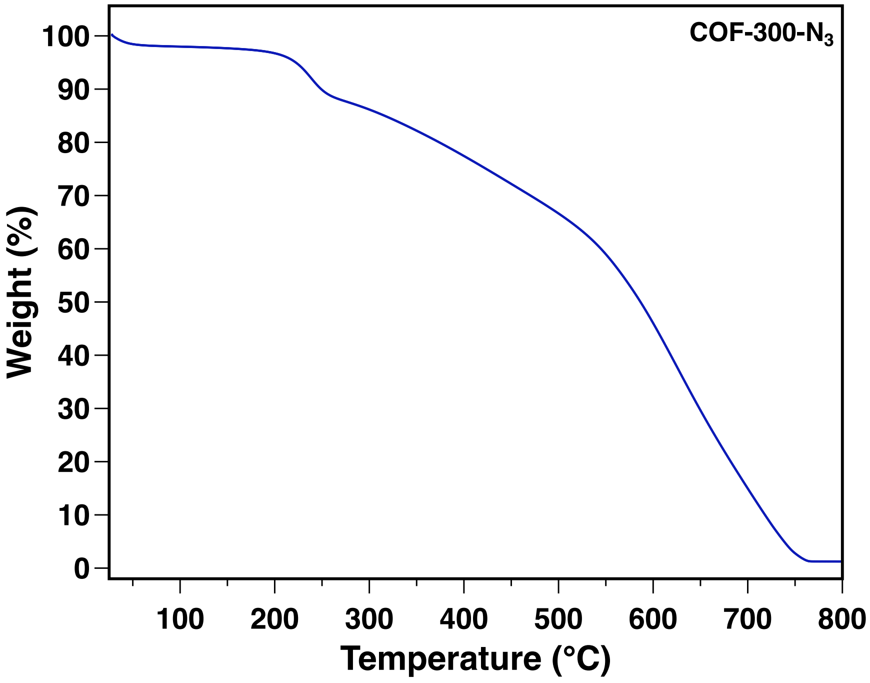


**Figure S22.** TGA of COF-300-$N_3$.

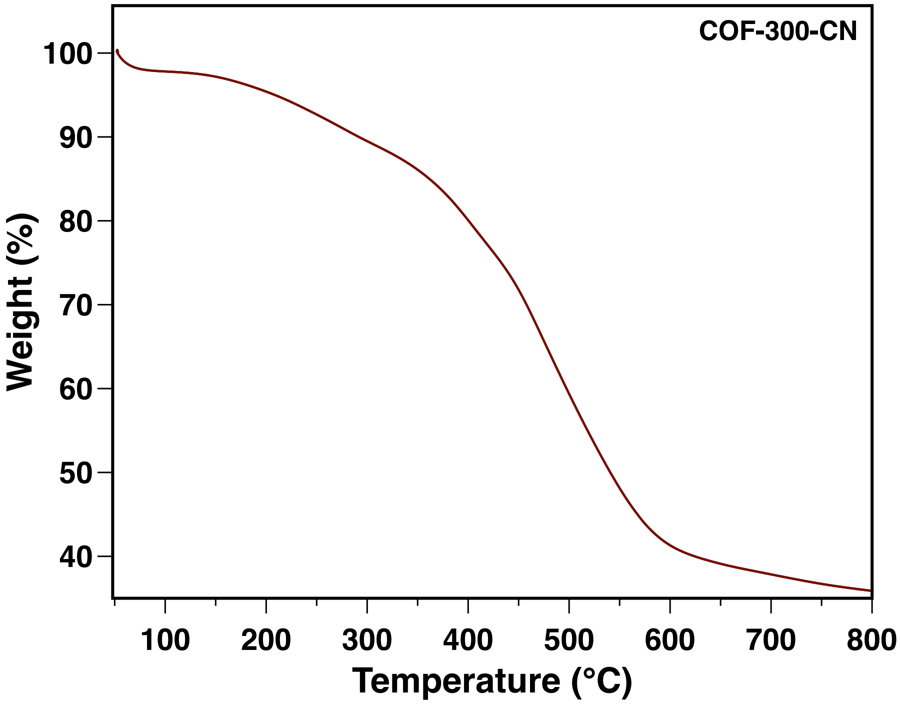


**Figure S23.** TGA of COF-300-CN.

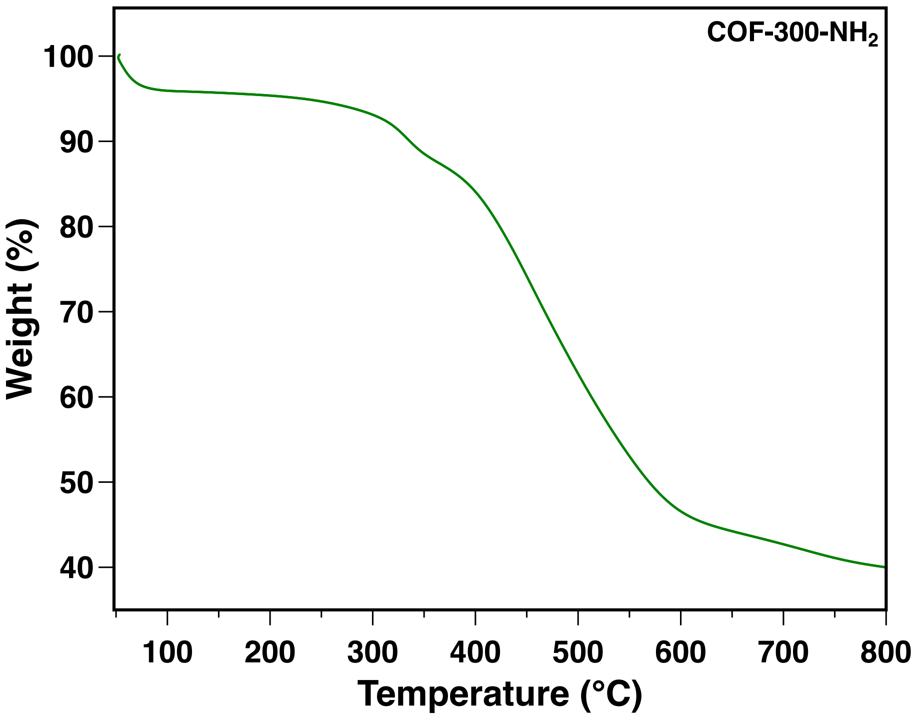


**Figure S24.** TGA of COF-300-$NH_2$.

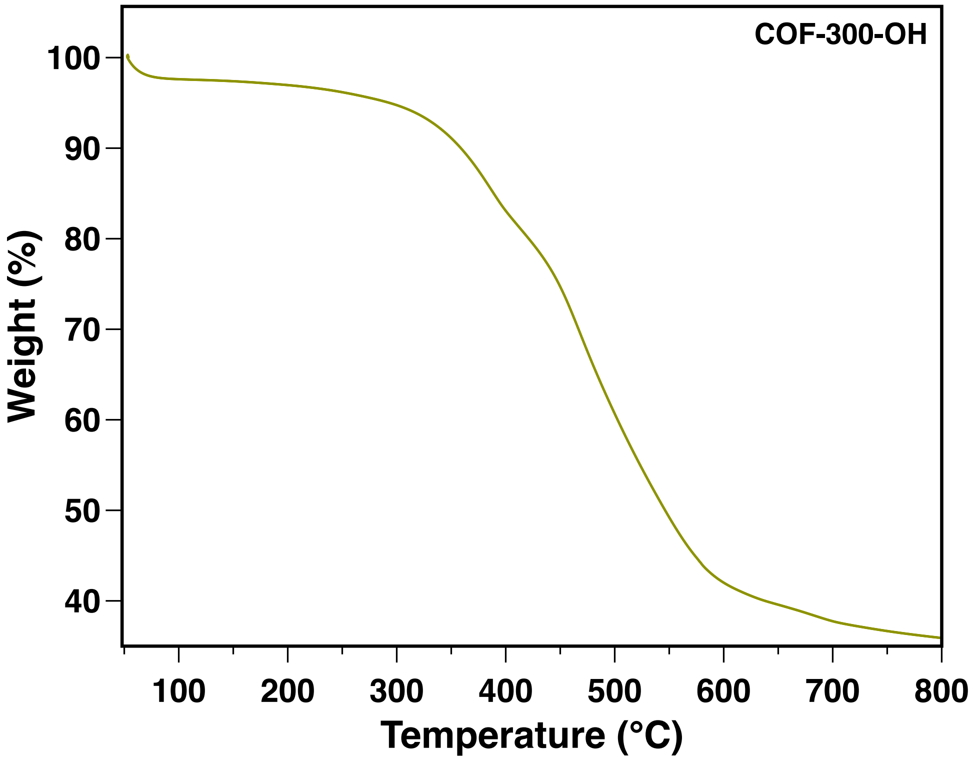


**Figure S25.** TGA of COF-300-OH.

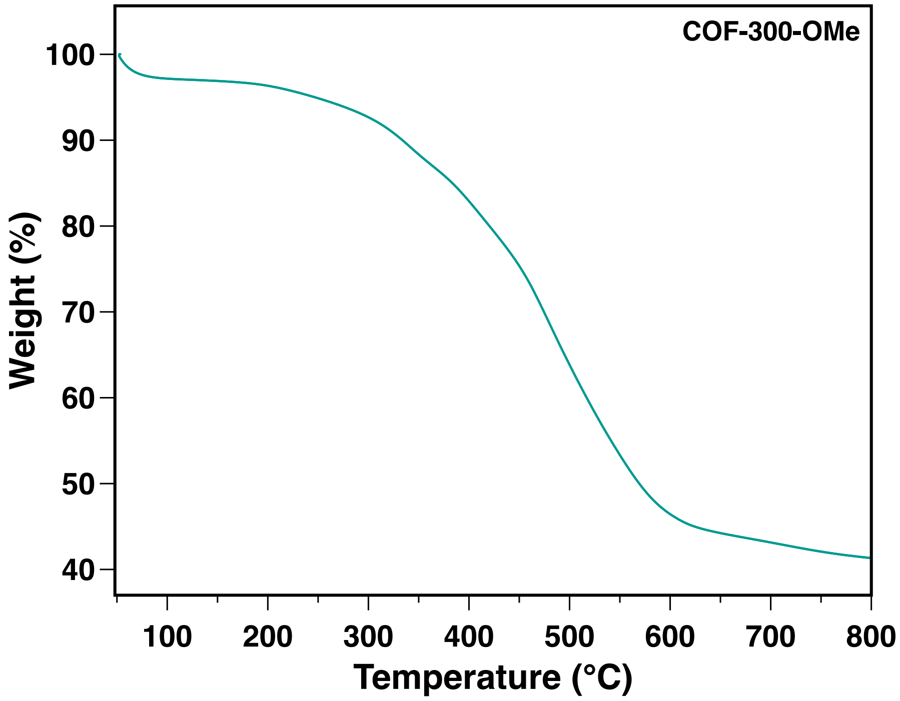


**Figure S26.** TGA of COF-300-OMe.

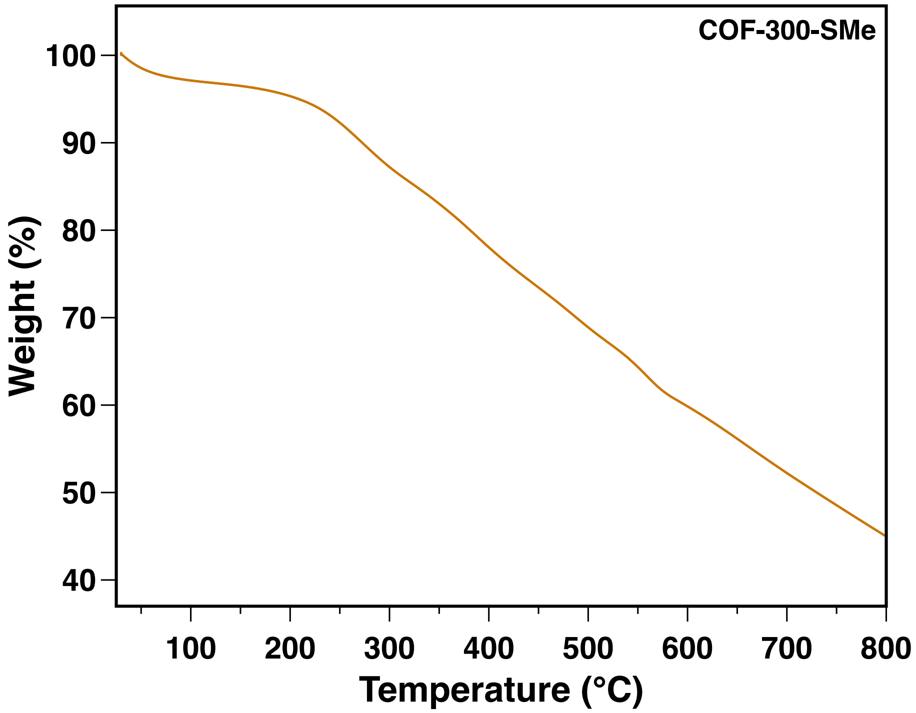


**Figure S27.** TGA of COF-300-SMe.

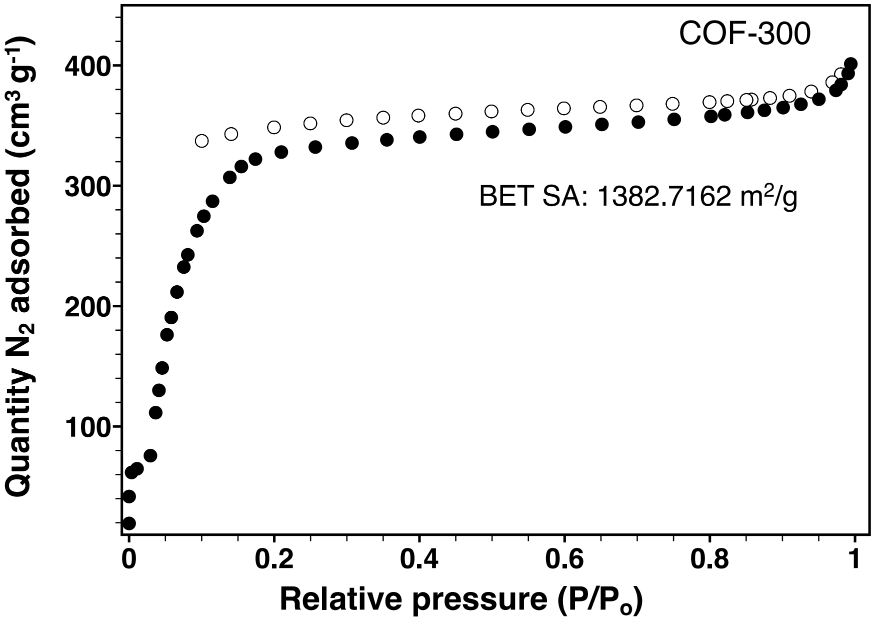


**Figure S28.** $N_2$ sorption isotherm of COF-300 at 77 K. Closed circles are adsorption and open circles are desorption.

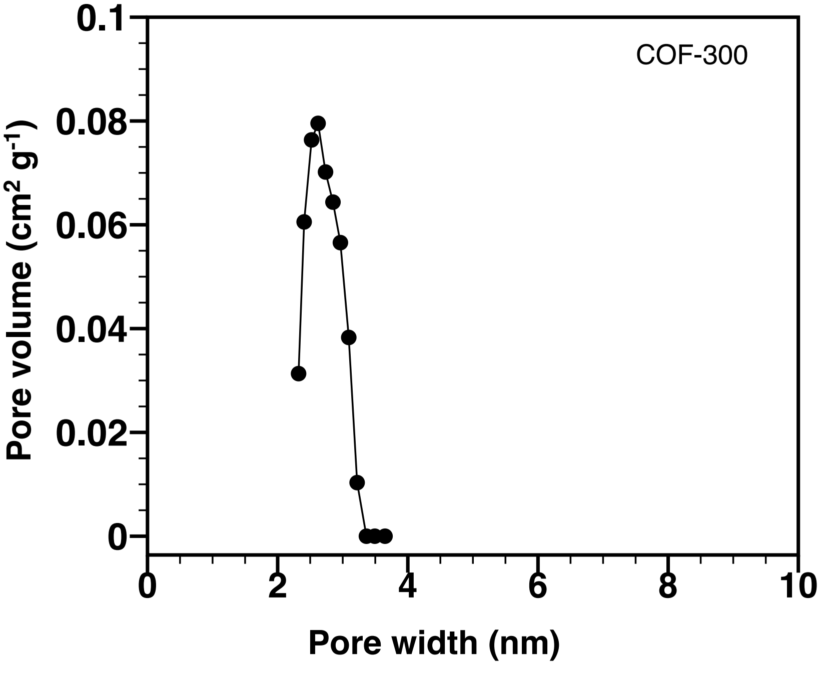


**Figure S29.** Pore size distribution of COF-300.

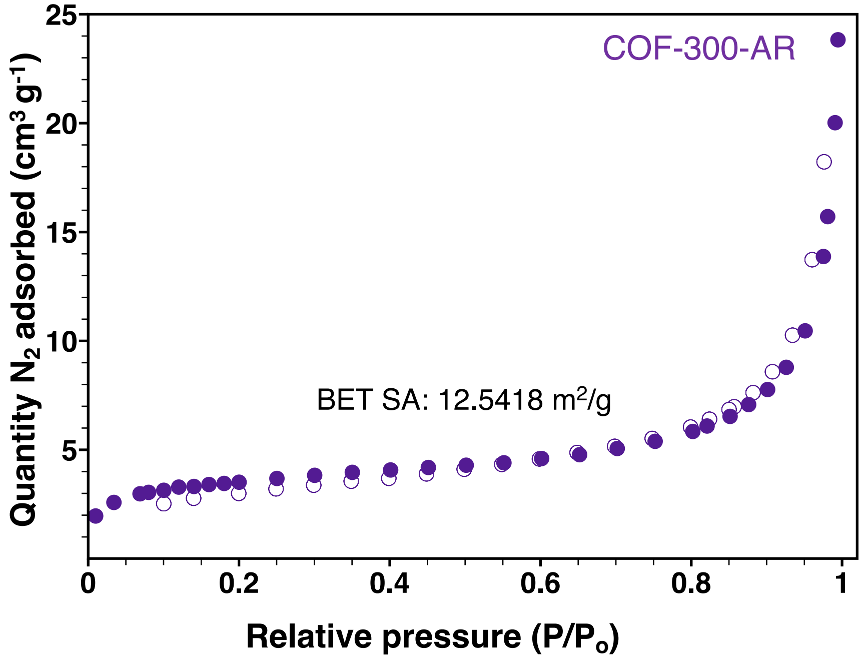


**Figure S30.** $N_2$ sorption isotherm of COF-300-AR at 77 . Closed circles are adsorption and open circles are desorption.

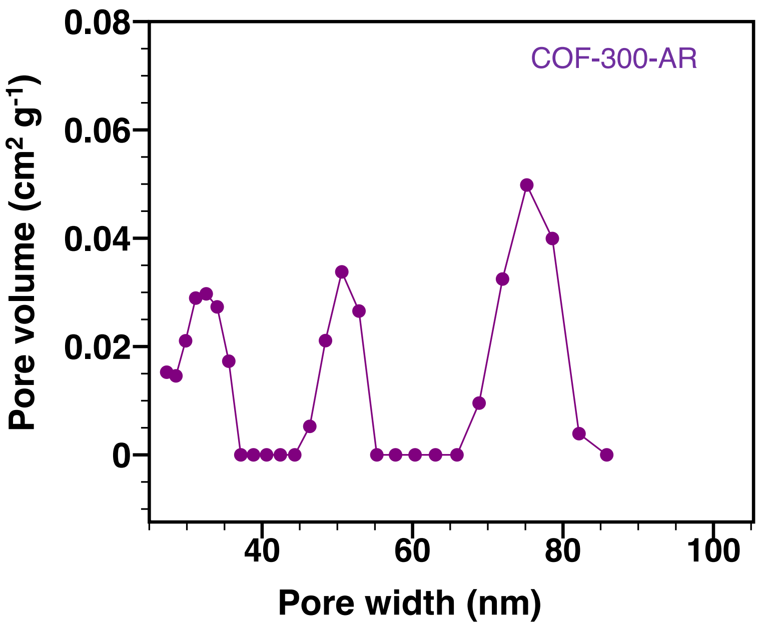


**Figure S31.** Pore size distribution of COF-300-AR.

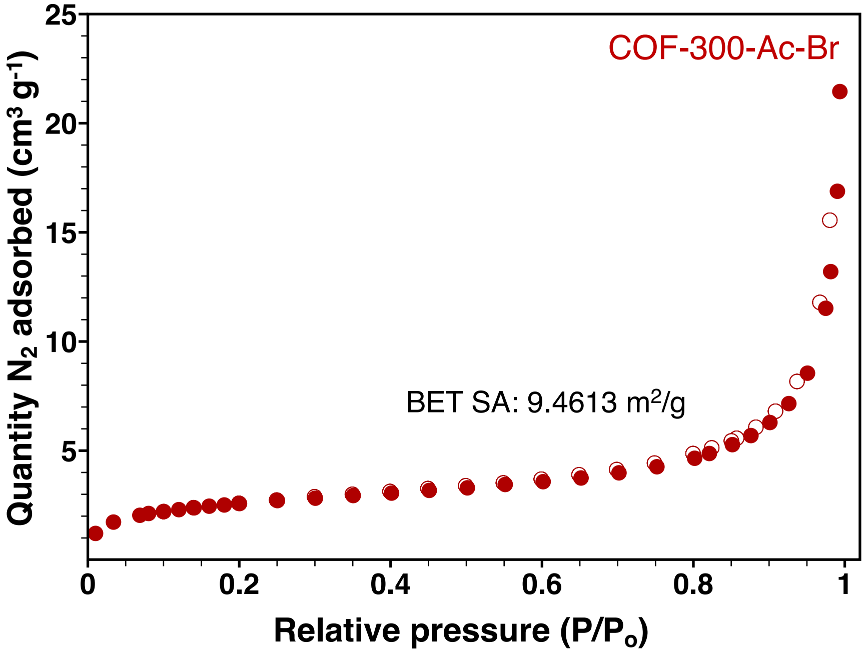


**Figure S32.** $N_2$ sorption isotherm of COF-300-Ac-Br at 77 K. Closed circles are adsorption and open circles are desorption.

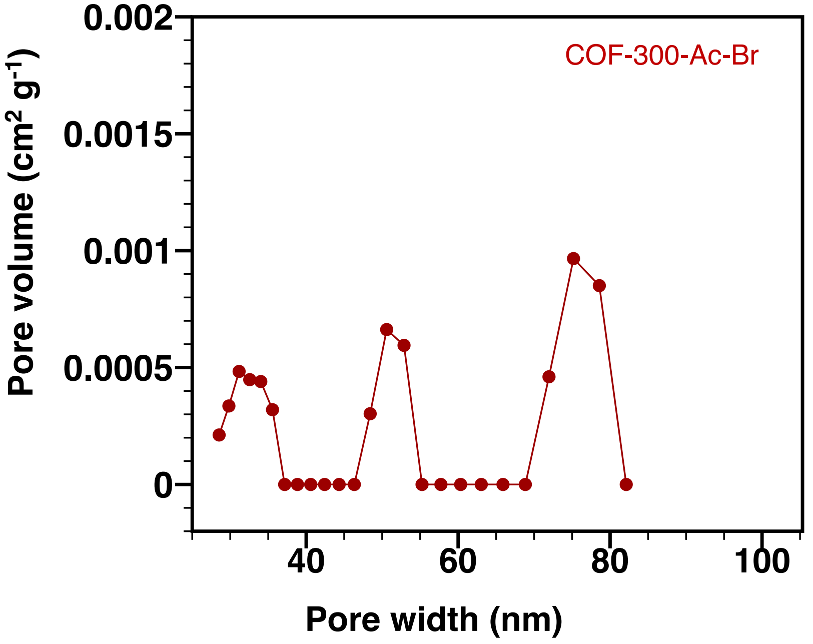


**Figure S33.** Pore size distribution of COF-300-Ac-Br

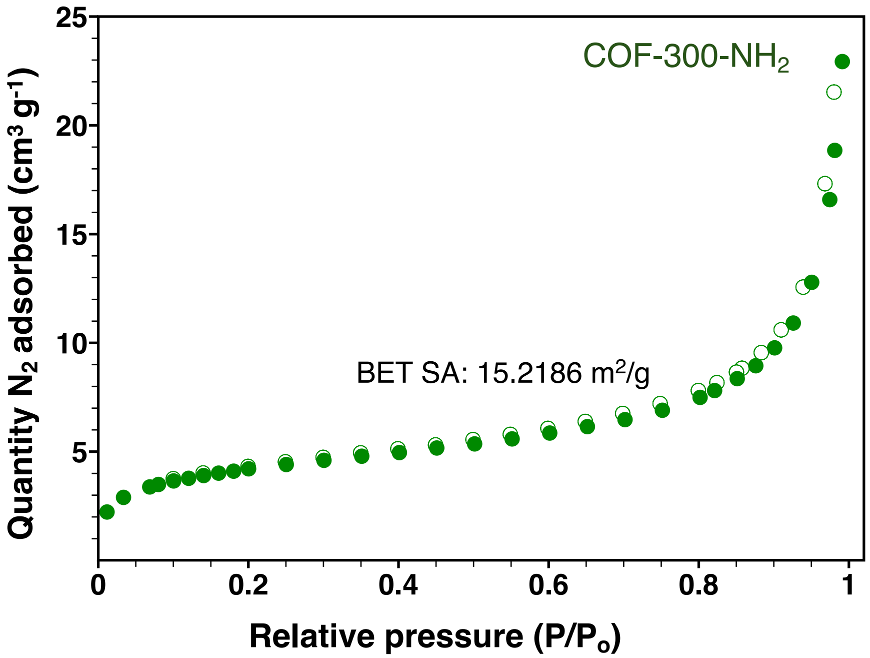


**Figure S34.** $N_2$ sorption isotherm of COF-300-$NH_2$ at 77 K. Closed circles are adsorption and open circles are desorption.

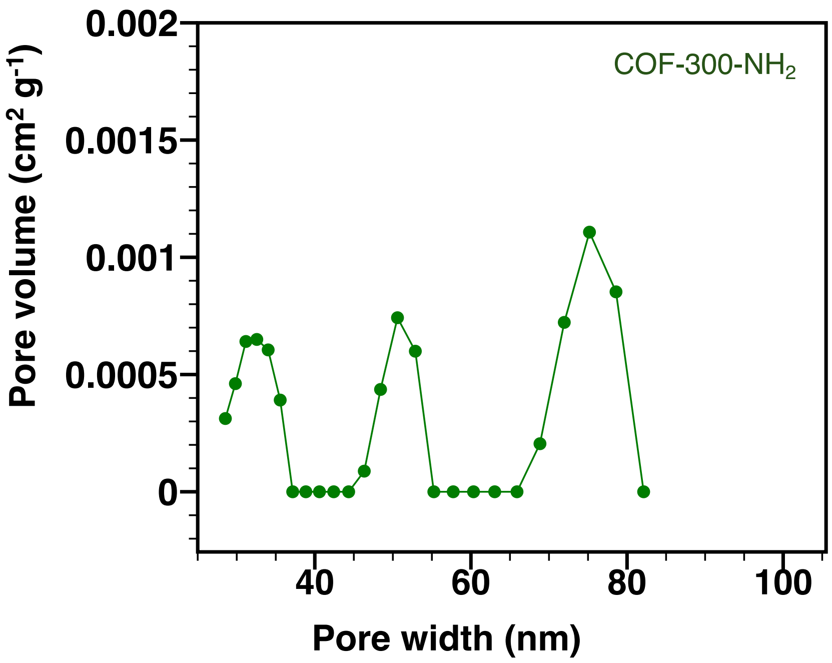


**Figure S35.** Pore size distribution of COF-300-$NH_2$.

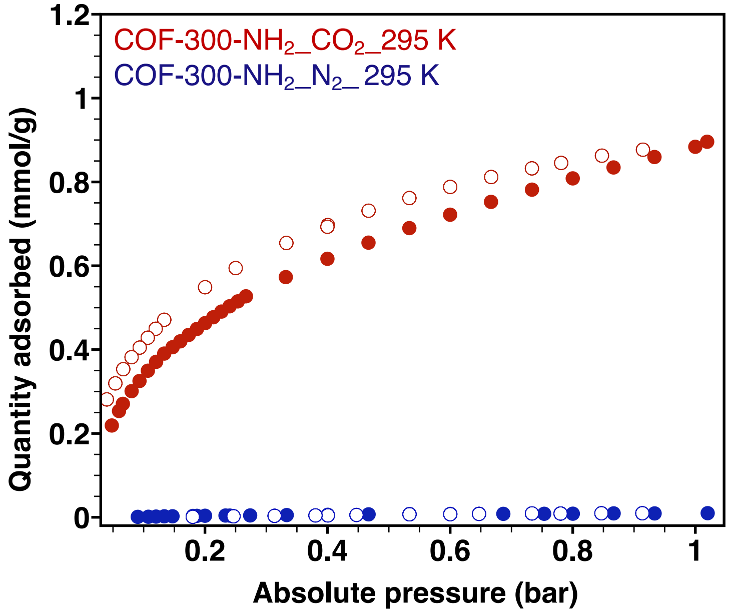


**Figure S36.** $CO_2$ and $N_2$ sorption isotherms at 295 K for COF-300-$NH_2$ showing selective adsorption of $CO_2$ at room temperature.

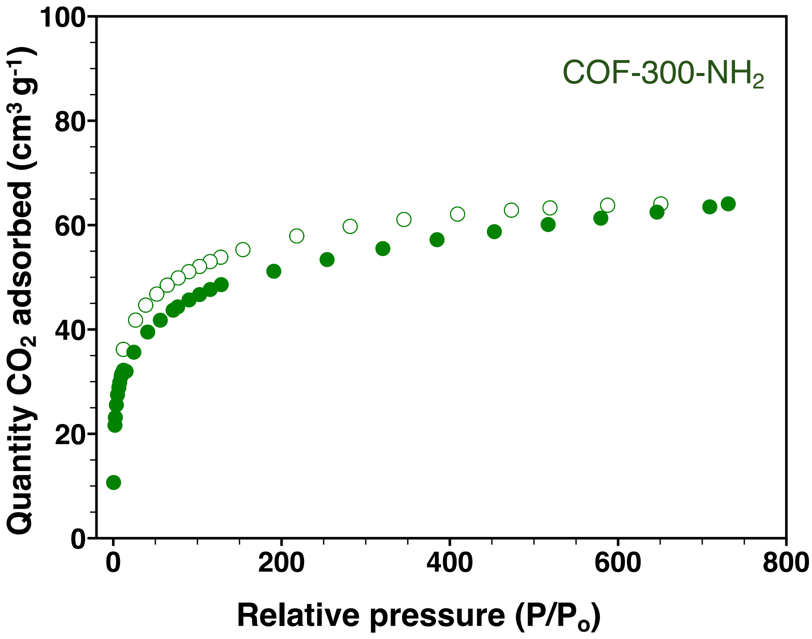


**Figure S37.** $CO_2$ sorption isotherm of COF-300-$NH_2$ at 195 K.

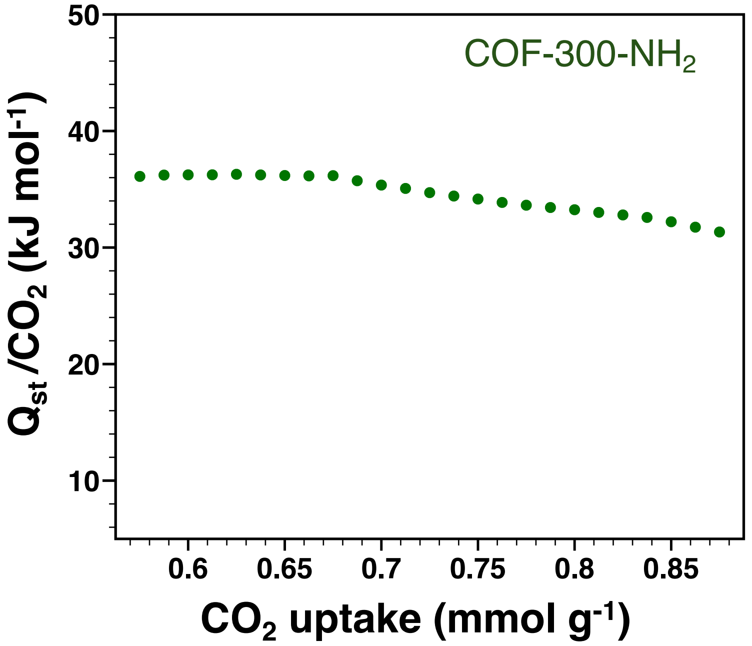


**Figure S38.** Isosteric heat of $CO_2$ adsorption ($Q_{st}$) for COF-300-NH$_2$ derived from 270 K and 295 K isotherms using the Clausius–Clapeyron method. $Q_{st}$ decreases from ~36 to ~31 kJ mol$^{-1}$ with increasing coverage, reflecting a distribution of adsorption sites and primarily physisorption $CO_2$ framework interactions that allows for easy regeneration.

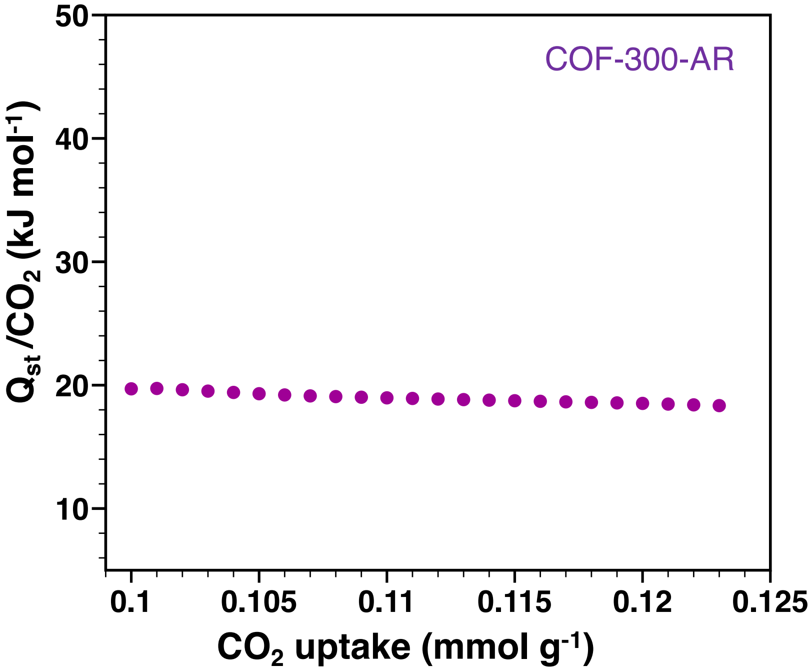


**Figure S39.** Isosteric heat of $CO_2$ adsorption (Qst) for COF-300-AR derived from 270 K and 295 K isotherms using the Clausius–Clapeyron method.

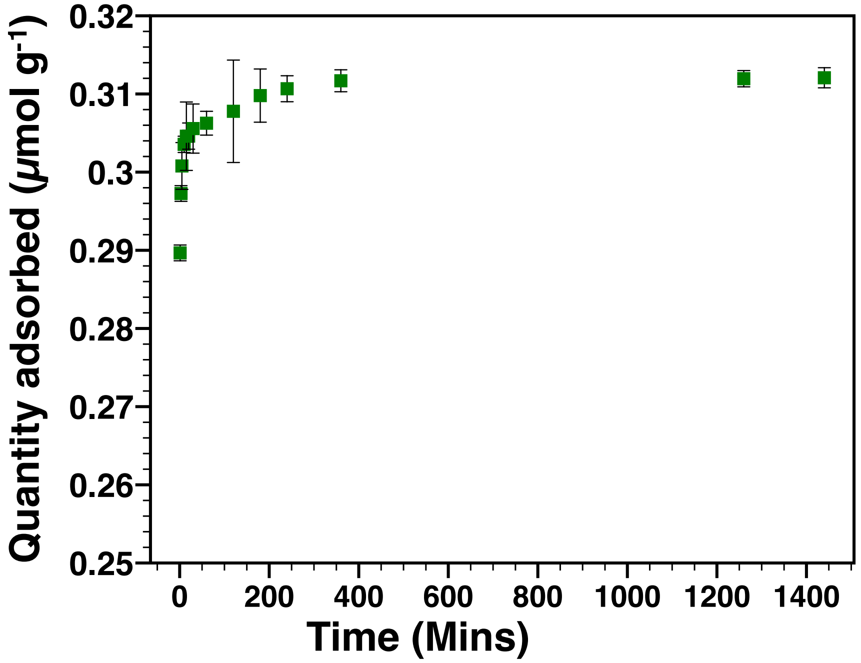


**Figure S40.** Time-dependent adsorption of PFBS by COF-300-$NH_2$. Rapid adsorption is observed within the first 20 min, which is then followed by a slower approach to equilibrium, reaching ~0.31 μmol $g^{-1}$ after 24 h.

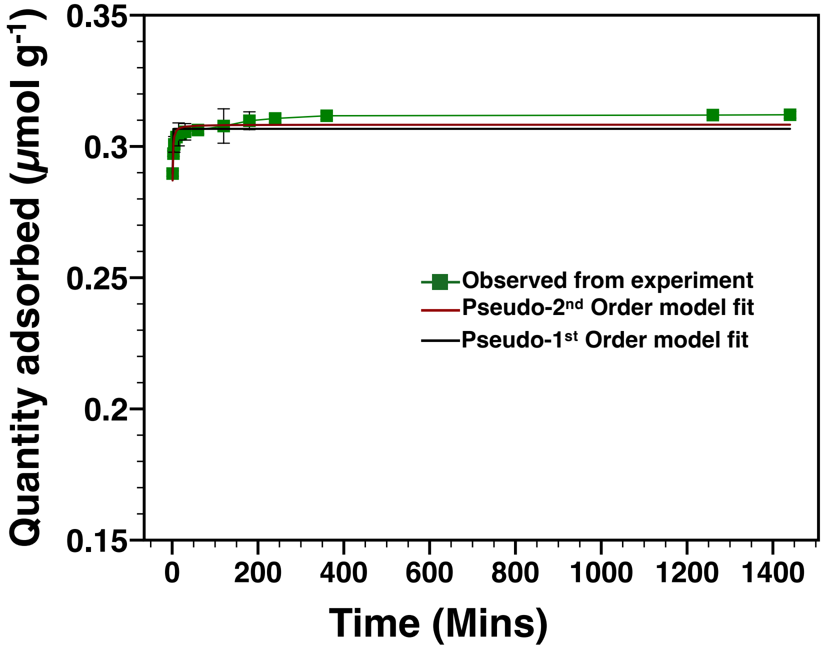


**Figure S41.** Experimental adsorption data of PFBS by COF-300-$NH_2$ fitted with pseudo-first-order and pseudo-second-order kinetic models. The pseudo-first-order model provides a closer match to the experimental curve, indicating better kinetic agreement.

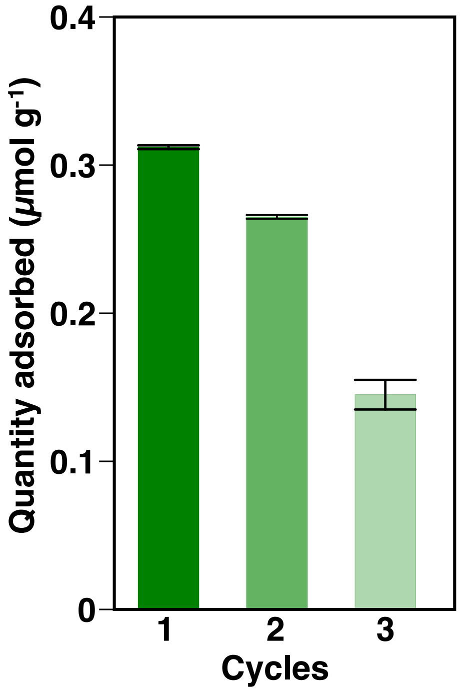


**Figure S42.** Reusability of COF-300-$NH_2$ for PFBS adsorption over three cycles.

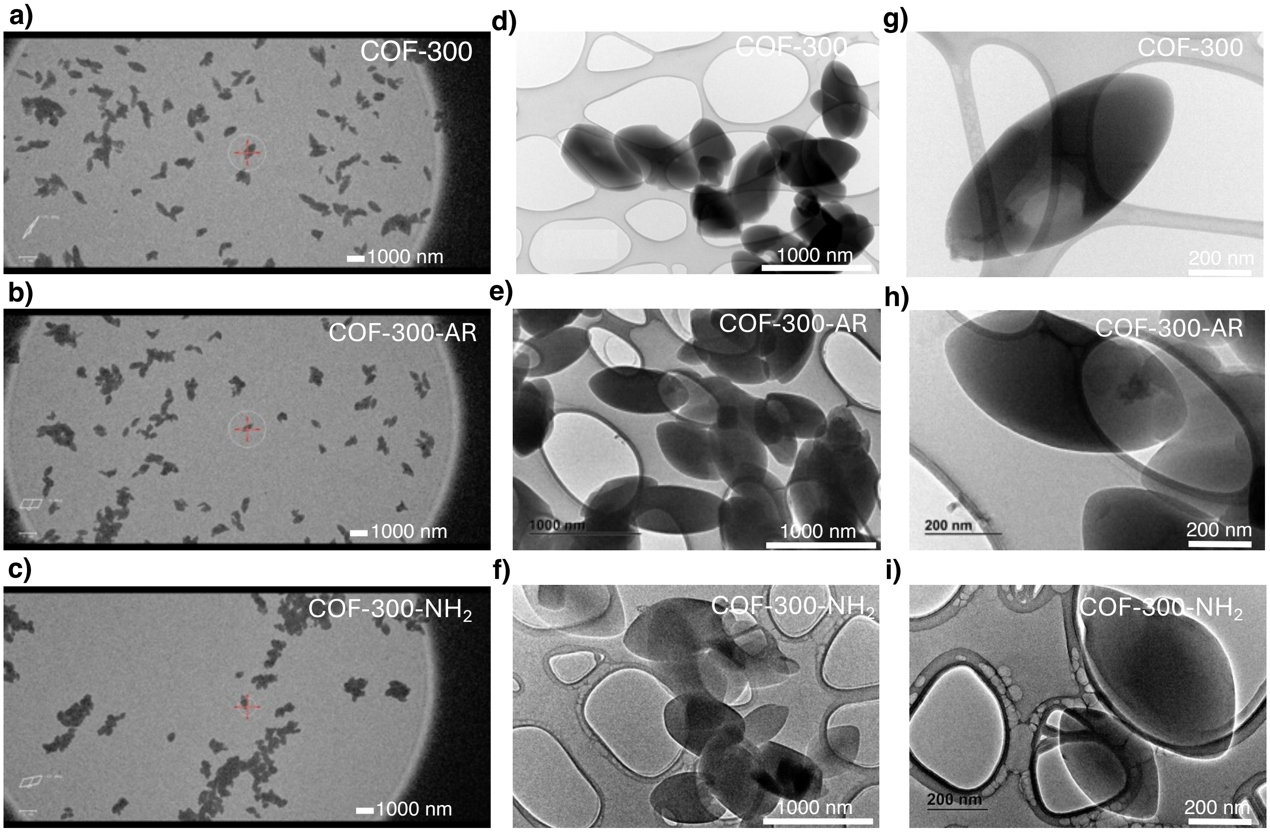


**Figure S43:** 3D micro electron diffraction grain image of COF-300 (a), COF-300-AR (b) and COF-300-$NH_2$ (c). Bright field transmission electron microscopy images of COF-300 (d, g), COF-300-AR (e, h) and COF-300-$NH_2$ (f, i)

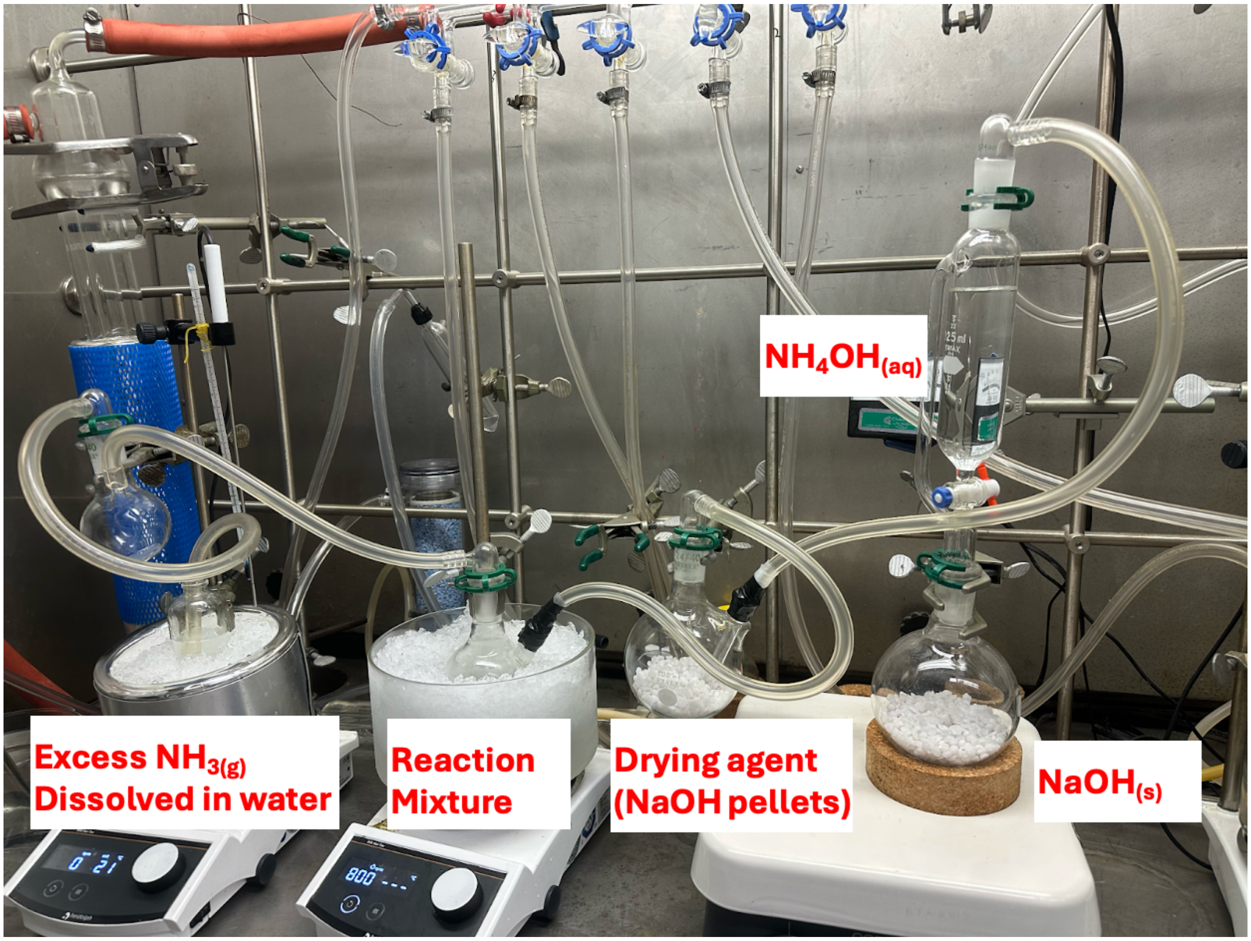


**Figure S44.** Reaction set-up for in situ $NH_{3(g)}$ generation used in the synthesis of COF-300-$NH_2$ and the model compound 2-amino-*N*-benzyl-*N*-phenylacetamide.

## ORTEP-style illustrations and A- and/or B-level Alerts Justifications

**COF-300-AR**
CCDC Number: 2480198
A- and/or B-level Alerts:
Problem: PLAT082_ALERT_2_A High R1 Value .................................. 0.40 Report
Response: The elevated R1 value reflects the limited completeness and the presence of unresolved disorder within the structure, both of which are common diffraction data. The core framework remains well-resolved, and the model is chemically plausible.

Problem: PLAT084_ALERT_3_A High wR2 Value (i.e. > 0.25) ................... 0.78 Report
Response: The high wR2 value arises from the lower resolution and of ED structure Restraints were applied to maintain chemically meaningful geometry. Despite the elevated value, the model accurately represents the underlying framework.

Problem: PLAT411_ALERT_2_A Short Inter H...H Contact H7B ..H7B . 1.69 Ang. -1/2-x,1/2-y,-1/2-z = 13_454 Check
Response: This close contact is due to modeled hydrogen positions within a region which is correlated by symmetry elements. Hydrogen atoms were placed using riding models and are not refined independently. The short contact does not indicate a chemically unrealistic interaction.

Problem: THETM01_ALERT_3_B The value of sine(theta_max)/wavelength is less than 0.575
Calculated sin(theta_max)/wavelength = 0.5563
Response: This structure was determined using three-dimensional electron diffraction data. The resolution limit is inherent to the technique due to constraints such as crystal size, radiation sensitivity, and tilt limitations. The resulting model is chemically reasonable and consistent with the observed electron diffraction data.

Problem: PLAT029_ALERT_3_B _diffrn_measured_fraction_theta_full value Low . 0.946 Why?
Response: The slightly low value is due to the limited goniometer tilt range during electron diffraction data collection. The 94.6% completeness is decent, the dataset was sufficient to solve and refine the structure reliably.

Problem: PLAT340_ALERT_3_B Low Bond Precision on C-C Bonds ............... 0.02686 Ang.
Response: The moderate bond precision is a result of the resolution and completeness limits of the ED dataset. Standard geometric restraints (DFIX, SADI) were applied where appropriate to maintain chemically reasonable bond length.

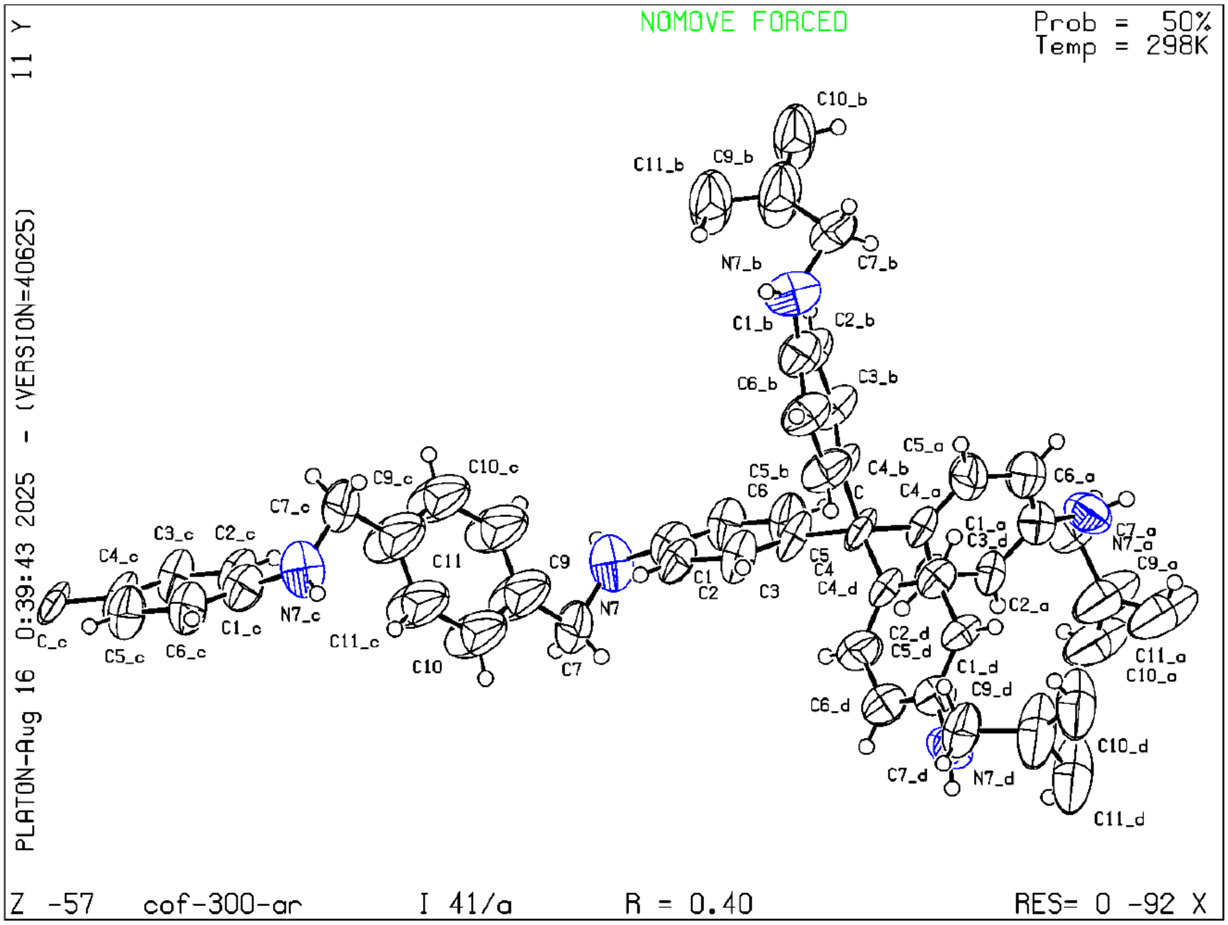


**Figure S45.** ORTEP-style illustration of COF-300-AR.

**COF-300-$NH_2$**
CCDC Number: 2480199
A- and/or B-level Alerts:
Problem: THETM01_ALERT_3_A The value of sine(theta_max)/wavelength is less than 0.550 Calculated sin(theta_max)/wavelength = 0.4513
Response: This structure was refined using three-dimensional electron diffraction (3D ED) data, which is typically limited in resolution due to crystal size, beam sensitivity, and goniometer tilt constraints. The low sine(theta_max)/wavelength value reflects these limitations and is common in ED-based structure determinations. The model remains chemically reasonable and consistent with the observed diffraction data.

Problem: PLAT082_ALERT_2_A High R1 Value .................................. 0.47 Report
Response: The high R1 value is expected for structures refined against 3D electron diffraction data, which are often affected by dynamical scattering, limited completeness, and residual disorder. Despite this, the core framework model is chemically plausible and supports the intended structural conclusions.

Problem: PLAT084_ALERT_3_A High wR2 Value (i.e. > 0.25) ................... 0.89 Report
Response: The elevated wR2 value results from the limited resolution and inherent noise in the electron diffraction dataset. Extensive geometric restraints were applied to maintain chemically meaningful bond lengths and angles. The model reflects the best interpretation of the available data.

Problem: PLAT340_ALERT_3_A Low Bond Precision on C-C Bonds ............... 0.05455 Ang.
Response: The low bond precision is attributed to the moderate resolution and limited number of unique reflections obtained in the ED dataset. Standard restraints such as DFIX and SADI were used to ensure chemically reasonable geometry, particularly in aromatic and disordered regions.

Problem: PLAT026_ALERT_3_B Ratio Observed / Unique Reflections (too) Low .. 38% Check
Response: The low observed-to-unique reflection ratio is a result of limited tilt range and crystal orientation in the electron diffraction experiment. These constraints are common in 3D ED data collection but still allow for a reliable structural model with the available reflections.

Problem: PLAT088_ALERT_3_B Poor Data / Parameter Ratio .................... 6.22 Note
Response: The low data-to-parameter ratio arises from modeling applying anisotropy refinement to non-hydrogen atoms. Restraints were applied to stabilize refinements and ensure chemically reasonable geometry.

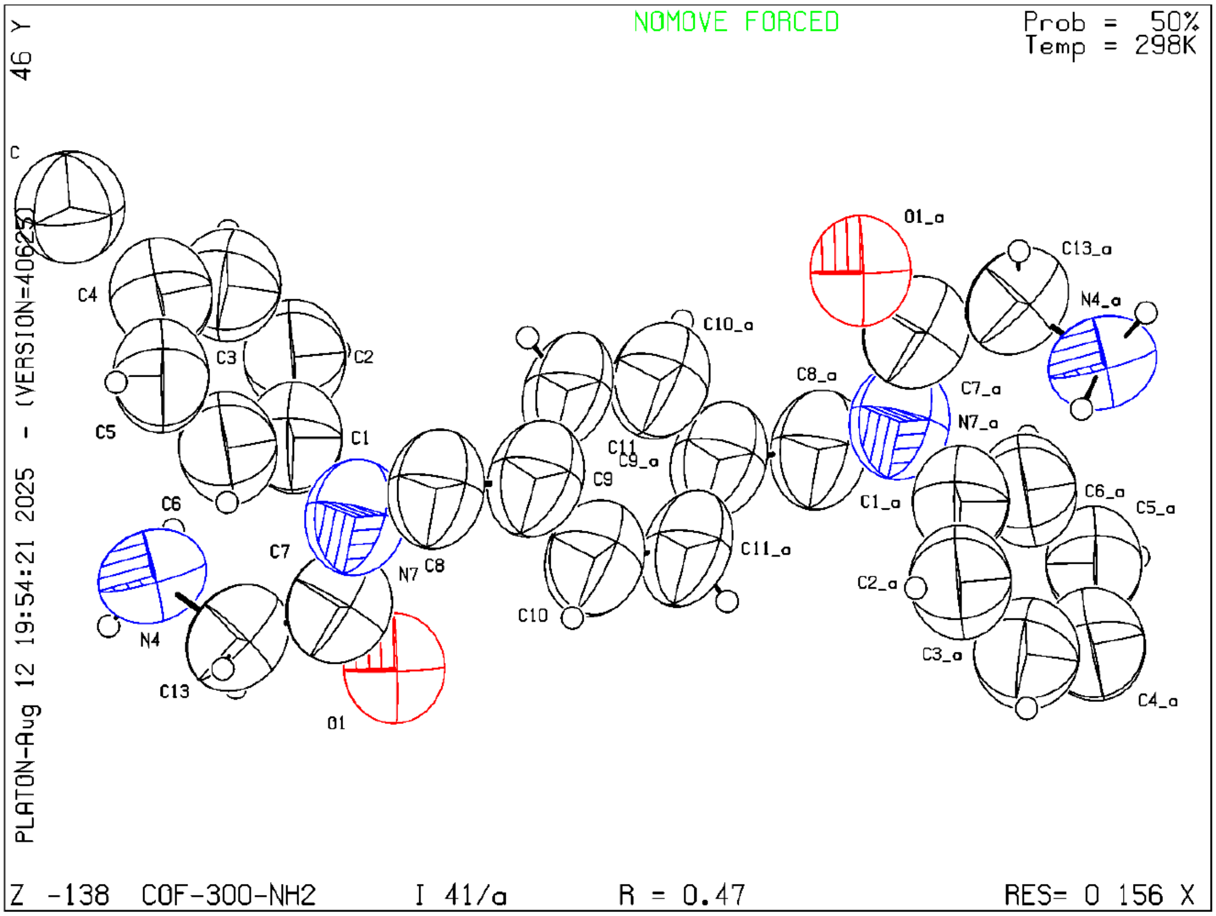


**Figure S46.** ORTEP-style illustration of $COF\text{-}300\text{-}NH_2$.

## Supplementary Tables

**Table S1:** Crystallographic data of COF-300-AR and COF-300-$NH_2$ from microED analysis

| Identification code | COF-300-AR | COF-300-$NH_2$ |
|---|---|---|
| Empirical formula | $C_{41}H_{36}N_4$ | $C_{49}H_{40}N_8O_4$ |
| Formula weight | 714.48 | 804.91 |
| Temperature/K | 298.00(10) | 298.00(10) |
| Crystal system | tetragonal | tetragonal |
| Space group | $I4_1/a$ | $I4_1/a$ |
| a/Å | 20.352(5) | 24.370(13) |
| b/Å | 20.352(5) | 24.370(13) |
| c/Å | 7.3547(17) | 7.002(3) |
| α/° | 90 | 90 |
| β/° | 90 | 90 |
| γ/° | 90 | 90 |
| Volume/$Å^3$ | 3046.4(16) | 4158(5) |
| Z | 4 | 4 |
| $\rho_{calc}$g/$cm^3$ | 1.558 | 1.286 |
| μ/$mm^{-1}$ | 0 | 0 |
| F(000) | 592 | 664 |
| Crystal size/$mm^3$ | 0.0006 × 0.0004 × 0.0003 | 0.0006 × 0.0004 × 0.0003 |
| Radiation | electron (λ = 0.0251) | electron (λ = 0.0251) |
| 2Θ range for data collection/° | 0.142 to 1.6 | 0.118 to 1.298 |
| Index ranges | $-19 \leq h \leq 19, -22 \leq k \leq 22, -8 \leq l \leq 8$ | $-18 \leq h \leq 19, -22 \leq k \leq 22, -6 \leq l \leq 6$ |
| Reflections collected | 4603 | 3104 |
| Independent reflections | 1040 [$R_{int}$ = 0.1449, $R_{sigma}$ = 0.1915] | 790 [$R_{int}$ = 0.1695, $R_{sigma}$ = 0.1648] |
| Data/restraints/parameters | 1040/108/90 | 790/246/127 |
| Goodness-of-fit on $F^2$ | 1.095 | 1.104 |
| Final R indexes [I>=2σ (I)] | $R_1$ = 0.3991, $wR_2$ = 0.7564 | $R_1$ = 0.4691, $wR_2$ = 0.8312 |
| Final R indexes [all data] | $R_1$ = 0.4257, $wR_2$ = 0.7775 | $R_1$ = 0.5391, $wR_2$ = 0.8883 |
| Largest diff. peak/hole / e $Å^{-3}$ | 0.26/-0.28 | 0.22/-0.24 |
| CCDC number | 2480198 | 2480199 |

**Table S2:** Averaged PFAS adsorption values of COFs: Equilibrium removal percentage (%) of PFAS by equal mass concentrations of COF-300, COF-300-AR and COF-300-$NH_2$ after 18 h at 23 °C, pH=7. ( $[PFAS]_0$ = 100 ppb, [COF] = 1 mg/1 g of PFAS solution.

| PFAS | COF-300 | COF-300-AR | COF-300-$NH_2$ |
|---|---|---|---|
| PFBA(%) | 7.5 ± 0.1 | 6.8 ± 1.9 | 19.0 ± 5.1 |
| PFBS(%) | 10.2 ± 1.8 | 0.15 ± 0.1 | 58.7 ± 1.1 |
| PFOA(%) | 13.4 ± 0.9 | 12.5 ± 0.2 | 63.4 ± 1.0 |

**Table S3:** COF-300-$NH_2$ PFBS adsorption measured at different times at 23 °C, pH=7, $[PFBS]_0$ = 100 ppb, [COF] = 1 mg/1 g of solution.

| Time (mins) | Quantity adsorbed (μmol $g^{-1}$) |
|---|---|
| 0 | 0 |
| 1 | 0.290 ± 0.001 |
| 3 | 0.297± 0.001 |
| 5 | 0.301± 0.003 |
| 10 | 0.304± 0.001 |
| 15 | 0.305± 0.004 |
| 20 | 0.305± 0.002 |
| 30 | 0.306± 0.003 |
| 60 | 0.306± 0.002 |
| 120 | 0.308± 0.007 |
| 180 | 0.310± 0.003 |
| 240 | 0.311± 0.002 |
| 360 | 0.312± 0.001 |
| 1260 | 0.312± 0.001 |
| 1440 | 0.312± 0.001 |

* $m_o$= 0.005 mg, $m_{COF}$ = 0.05 g, $MW$(PFBS) = 300.1 $gmol^{-1}$

**Table S4:** COF-300-$NH_2$ PFBS adsorption measured at different cycles at 23 °C, pH=7, $[PFBS]_0$ = 100 ppb, [COF] = 1 mg/1 g of solution.

| Cycles | Quantity adsorbed (μmol $g^{-1}$) |
|---|---|
| 1 | 0.312± 0.001 |
| 2 | 0.265 ± 0.001 |
| 3 | 0.145± 0.010 |

* Each cycle time = 24 hours.

**Table S5:** Rate model parameters for PFBS adsorption with COF-300-$NH_2$.

| Variables | Pseudo 1$^{st}$ Order model | Pseudo 2$^{nd}$ Order model |
|---|---|---|
| $q_e$ | 0.3067 μmol $g^{-1}$ | 0.3083 μmol $g^{-1}$ |
| $k$ | 2.8858 $min^{-1}$ | 43.7581 g $μmol^{-1}$ $min^{-1}$ |
| $R^2$ | 0.9970 | 0.9990 |

## References


[1] K. Korathotage, N. Yamamoto, E. D. Bloch, *Inorganic Chemistry* **2024**, *63*, 23698–23704.

[2] W. Wang, Y. Jia, S. Zhou, S. Deng, *Journal of Hazardous Materials* **2023**, *460*, 132522– 132522.

[3] P. Ganesan, X. Yang, J. Loos, T. J. Savenije, R. D. Abellon, H. Zuilhof, E. J. R. Sudhölter, *J. Am. Chem. Soc.* **2005**, *127*, 14530–14531.

[4] C. Klumpen, M. Breunig, T. Homburg, N. Stock, J. Senker, *Chem. Mater.* **2016**, *28*, 5461–5470.

[5] F. D. Bellamy, K. Ou, *Tetrahedron Lett.* **1984**, *25*, 839–842.

[6] F. J. Uribe-Romo, J. R. Hunt, H. Furukawa, C. Klöck, M. O'Keeffe, O. M. Yaghi, *J. Am. Chem. Soc.* **2009**, *131*, 4570–4571.

[7] H. Liu, J. Chu, Z. Yin, X. Cai, L. Zhuang, H. Deng, *Chem* **2018**, *4*, 1696–1709.
